# Large-scale transcriptional profiling of lignified tissues in *Tectona grandis*

**DOI:** 10.1186/s12870-015-0599-x

**Published:** 2015-09-15

**Authors:** Esteban Galeano, Tarcísio Sales Vasconcelos, Mabel Vidal, Maria Katherine Mejia-Guerra, Helaine Carrer

**Affiliations:** Laboratório de Biotecnologia Agrícola (CEBTEC), Departamento de Ciências Biológicas, Escola Superior de Agricultura “Luiz de Queiroz”, Universidade de São Paulo, Av. Pádua Dias, 11, Piracicaba, São Paulo 13418-900 Brazil; CAPS Computational Biology Laboratory (CCBL), Center for Applied Plant Sciences, Ohio State University, 206 Rightmire Hall, 1060 Carmack Road, Columbus, Ohio 43210 United States

## Abstract

**Background:**

Currently, *Tectona grandis* is one of the most valuable trees in the world and no transcript dataset related to secondary xylem is available. Considering how important the secondary xylem and sapwood transition from young to mature trees is, little is known about the expression differences between those successional processes and which transcription factors could regulate lignin biosynthesis in this tropical tree. Although *MYB* transcription factors are one of the largest superfamilies in plants related to secondary metabolism, it has not yet been characterized in teak. These results will open new perspectives for studies of diversity, ecology, breeding and genomic programs aiming to understand deeply the biology of this species.

**Results:**

We present a widely expressed gene catalog for *T. grandis* using Illumina technology and the de novo assembly. A total of 462,260 transcripts were obtained, with 1,502 and 931 genes differentially expressed for stem and branch secondary xylem, respectively, during age transition. Analysis of stem and branch secondary xylem indicates substantial similarity in gene ontologies including carbohydrate enzymes, response to stress, protein binding, and allowed us to find transcription factors and heat-shock proteins differentially expressed. TgMYB1 displays a MYB domain and a predicted coiled-coil (CC) domain, while TgMYB2, TgMYB3 and TgMYB4 showed R2R3-MYB domain and grouped with MYBs from several gymnosperms and flowering plants. *TgMYB1*, *TgMYB4* and *TgCES* presented higher expression in mature secondary xylem, in contrast with *TgMYB2, TgHsp1, TgHsp2, TgHsp3,* and *TgBi* whose expression is higher in young lignified tissues. *TgMYB3* is expressed at lower level in secondary xylem.

**Conclusions:**

Expression patterns of *MYB* transcription factors and heat-shock proteins in lignified tissues are dissimilar when tree development was evaluated, obtaining more expression of *TgMYB1* and *TgMYB4* in lignified tissues of 60-year-old trees, and more expression in *TgHsp1, TgHsp2, TgHsp3* and *TgBi* in stem secondary xylem of 12-year-old trees. We are opening a door for further functional characterization by reverse genetics and marker-assisted selection with those genes. Investigation of some of the key regulators of lignin biosynthesis in teak, however, could be a valuable step towards understanding how rigidity of teak wood and extractives content are different from most other woods. The obtained transcriptome data represents new sequences of *T. grandis* deposited in public databases, representing an unprecedented opportunity to discover several related-genes associated with secondary xylem such as transcription factors and stress-related genes in a tropical tree.

**Electronic supplementary material:**

The online version of this article (doi:10.1186/s12870-015-0599-x) contains supplementary material, which is available to authorized users.

## Background

Teak (*Tectona grandis* Linn. f.) (Lamiaceae) is the most important and highly valued commercial hardwood timber in the tropics due to its high durability, dimensional stability, heartwood-sapwood proportions, weightlessness and resistance to weathering. Also, it is used for carpentry, floors, shipbuilding and agroforestry, thus becoming a high-class furniture and a standard timber in end-use classification of other tropical timbers [1–3]. It is a deciduous species presenting natural populations in Thailand, Laos, Myanmar, India and Java Islands. Teak grows properly within 25-38 °C, between 1,250 and 2,500 mm/year of rainfall, presenting the best yields under 600 m above sea level and produces better wood quality with long dry periods, from 3 to 5 month long [4–7]. This species is the major component of the forest economies of many tropical countries. It is the only valuable hardwood that constitutes a globally emerging forest resource with a planted area of 4,346 million ha (0,5 million m^3^ of wood) and natural forest of 29,035 million ha (2 million m^3^ of wood) around the world, and Brazil presents the largest teak reforestation in South America [7].

Due to its importance, many efforts have focused on the study of teak population variability [8–13]. However, there are no genetic studies nor next-generation sequencing regarding wood formation in teak. Wood comes from secondary growth, starting with the vascular cambium expansion and cell division in stems of young trees, followed by a differentiation of secondary xylem and several events such as xylem cells expansion, secondary cell wall deposition and programmed cell death [14–16]. In most tropical America, including Brazil, wood harvesting occurs at 20 years, producing small-dimension logs, which are not in demand on the international market [4, 7]. Teak is not a fast growing species but can produce a timber of optimum strength in relatively short rotations of 21 years [17] depending of the sapwood-heartwood percentages. The timber quality produced will be the overriding commercial factor for the near future [18], and usually relates to the amount, color and durability of the heartwood [4].

For that reason, techniques such as ESTs and microarrays have been used extensively to understand wood formation in trees such as *Pinus* [19] and *Populus* [15]. However, today, large-scale studies of biological phenomena are unthinkable without the use of next-generation sequencing technologies (NGS), such as RNA sequencing (RNA-seq), which encourages developmental and genomics research of woody growth in trees [16], especially for species without a sequenced genome and no molecular information available [20, 21] as teak. In tropical trees, the use of next-generation sequencing in order to find differentially expressed unigenes involved in secondary xylem is restricted to some species [22].

Availability of nondestructive wood analysis methods such as core sampling would provide a valuable way to study teak wood in different aspects and avoid depletion of both natural and plantation teak resources [5]. Heartwood and sapwood are complex tissues in which percentages are not easily assessed on standing trees, but they can be determined from a bore core [4]. Also, their study in the area of molecular biology is challenging because of their rigid woody tissues with high contents of polysaccharides, which hinders its maceration and extraction of genetic material. The sapwood is a heterogeneous tissue with a mixture of earlywood and latewood and differing levels of lignification. Sapwood is composed of xylem and other dead as well as living cells, reserves of starch or sugar and lower extractives content [23]. The same author explains that a larger proportion of sapwood is preferred in wood for pulp manufacture and preservative treatment, and heartwood is desirable in construction timber, high quality veneers and joinery because of its resistance to biotic attack and darker color. In a cross-section of logs, sapwood is usually observed as a pale annulus surrounding concentric heartwood [23].

In teak, it is certainly needed to identify genes such as those controlling secondary xylem, vessel formation, sapwood and heartwood differentiation, volume growth and abiotic stress. Those studies have been documented in *Populus tremula* [24], *Populus euphratica* [25], *Populus trichocarpa* [15, 26], eucalyptus [27], conifers [28, 29], and *Fraxinus spp*. [30], but it needs to be done in teak to help improving wood quality, growth speed and environmental adaptability [4]. The expression of several genes has been related to the wood formation processes, including some families of transcription factors [31]. The *MYB* transcription factors have been related to the coordination of genes which drive the lignin biosynthesis, with a great range of regulation and operating at all points of the phenylpropanoid pathway [32]. The R2R3-MYB proteins (characterized by two imperfect conserved repeats of ~50 amino acids) belong to a large family of transcription factors with over 120 members in angiosperms, also defined by an N-terminal DNA- binding domain (DBD), a C-terminal modulator region with regulatory activity; also R2R3-MYB proteins show a potential of binding AC elements (representative of lignin biosynthetic genes), which belong to the most abundant type in plants with essential roles in vascular organization [28, 33].

Therefore, genetic examination of the superior growth of a prized woody plant such as *T. grandis* would provide a collection of expressed genes from several tissues, as it has been done in another forestry species such as eucalyptus, where a digital expression profiling of xylogenic and non-xylogenic tissues was obtained via RNA-seq [27]. A better understanding of secondary xylem formation is essential not only as a fundamental part of plant biology (anatomy, biochemistry and at the genetic level), but also because it is crucial to obtain solutions for problems in forest conservation, improving the offerings of woody products [16]. Also, it is hoped that through genetic selection and plant transformation, the non-durable core could be reduced or eliminated, the growth could be increased and the epicormic branches could be controlled, making the so-called “juvenile wood” problem a thing of the past [6]. Sapwood/hardwood characteristics are reliable predictors of overall genetic improvement of timber strength [17]. Therefore, this is the first RNA sequencing in this tropical woody plant. Firstly, the aim of this study was to unveil the transcriptome of teak at a large-scale to later compare the transition of young (12 years old) to mature (60 years old) trees in order to reveal differentially expressed transcripts since this transition gives wood strength, endurance, color differences, natural chemicals and biotic and abiotic resistance to older trees, important features in the teak market. We detected 48,633 transcripts in stem secondary xylem and found that more than 2000 unigenes were differentially expressed in a temporal and tissue specific fashion. We also supplied several heat-shock proteins and analyzed the expression of some *MYB*-related transcription factors differentially expressed in teak secondary xylem, including sapwood tissue.

## Results

### Quality of the RNA and the reads

**Fig. 1:**
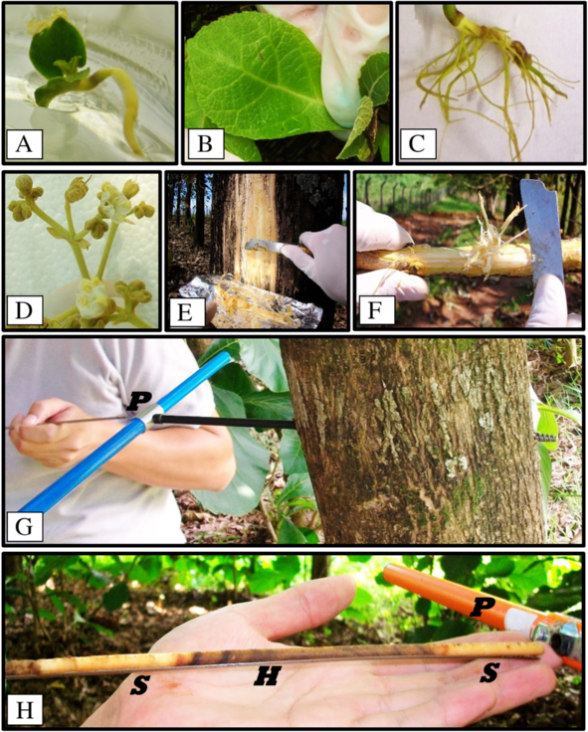
Teak tissue and organ sample set. **a** *In vitro* seedling. **b**) *In vitro* leaf. **c**) *In vitro* root. **d**) Flower. **e**) Stem secondary xylem. **f**) Branch secondary xylem. **g**) Use of pressler core barrel (“*P*”) at Diameter Breast High (DBH). **h**) Core sample containing “*S*” (sapwood) and “*H*” (heartwood). All samples were immediately placed on aluminum foil and transported in liquid nitrogen for a subsequent RNA extraction

Based on the bioanalyzer results (Additional file 1), all samples (Figure 1a-f) showed appropriate RIN factor. The libraries had a size of 280 bp, approximately. We generated almost 193 million paired-end reads, covering 38.6 Gigabases of sequence data with a sequence length of 100 bp (Table 1). The dataset of raw reads was deposited in NCBI SRA database under SRA study number SRP059970. After cleaning the data with the “trimmed” procedure [34], the “per base quality”, “per base sequence content”, “per sequence GC content”, “per sequence quality”, “duplication levels” and “sequence length distribution” were improved (Additional file 2). Then, 9.5 % of the reads (Table 1), and between 3.8 % (branch of 60-year-old teak trees) and 11.14 % (seedling) (Additional file 3) were lost after cleaning. More than 174 million sequence reads with a size of 34.9 Gigabases (Table 1) were obtained. Consequently, with this quality it was possible to continue the subsequent analyses (Additional file 2).

**Table 1:** Overview of sequencing, assembly, differential expressed genes and annotations

Raw data	
Total number of reads without cleaning	192,841,634
Size without cleaning approx. (Gigabases)	38.6
Sequence length without cleaning (bp)	100
Total number of reads after cleaning	174,528,668
Size after cleaning approx. (Gigabases)	34.9
Sequence length after cleaning (bp)	75-100
% Erased reads	9.5
Assembly with Trinity # transcripts	Contig N50 (bp)
Stem secondary xylem Transcriptome 48,633	2,291
Branch secondary xylem Transcriptome 59,771	2,365
Flower, leaf, root, seedling Transcriptome 65,592	2,178
Differential expression	
Number of most expressed transcripts in Stem second. Xyl.	1,502
Number of most expressed transcripts in Branch second. Xyl.	931
Annotations by Blast2Go	
Number of predicted CDS (partial/complete) in Stem second. Xyl.	669
Number of predicted CDS (partial/complete) in Branch second. Xyl.	603

### De novo assembly

The assembly of the transcriptome from the leaf, root, seedling, flower, secondary xylem of teak branch and stem was performed using the Trinity assembler [35]. For lignified tissues such as branch secondary xylem of both tree ages (12- and 60-year-old trees), we used between 9,622,608 and 16,324,986 reads, and for stem secondary xylem of both tree ages (12- and 60-years-old) we used between 9,417,573 and 10,963,888 reads (Additional file 3). Flower, leaf, root and seedling were 10,080,256, 12,955,867, 11,564,402 and 13,241,021 reads, respectively. Unpaired reads were from 1,508,503 (branch) to 3,699,463 (stem) in all samples. Using those reads as input for Trinity [35], we obtained 112,850, 139,535, 129,126 and 80,749 contigs for stem secondary xylem, branch secondary xylem, non-lignified tissues (root, flower, seedling, leaf) and unpaired reads, respectively, (Additional file 3), with a mean for N50 length of 2,140 bp. Contigs coming from lignified samples were subsequently used for differential expression analyses.

### Unigenes differentially expressed in lignified tissues between 12- and 60-year-old trees

**Fig. 2:**
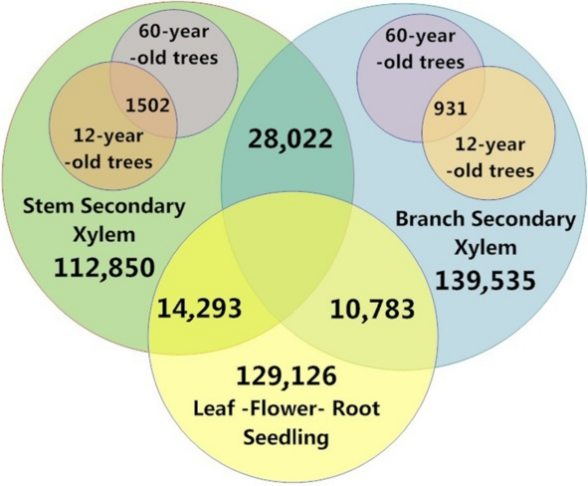
Venn Diagram showing number of differentially expressed genes in the different tissues and ages. For the diagram, we used leaf, flower, root, seedling, stem and branch secondary xylem, comparing young (12-years-old) and mature (60-years-old) trees for the last two tissues

Differentially expressed transcripts in all the comparison groups with DESeq program were obtained with a false discovery rate of 0.05 (Additional file 4, Fig. 2). In the case of the branch secondary xylem transcripts differentially expressed from both 12- and 60-year-old teak trees with repetitions, the dispersion plot (Additional file 4a) showed the presence of significant genes differentially expressed between both ages, showing a normalized grouping tendency in most of the transcripts with the fitted curve. Also, in Additional file 4b all the differentially expressed transcripts are exposed in red dots. The dispersion plot (Additional file 4c) of stem secondary xylem transcripts differentially expressed from both 12- and 60-year-old teak trees (with repetitions) showed a normalized grouping tendency with a fitted curve. Several differentially expressed transcripts in stem secondary xylem were also obtained (red dots, Additional file 4d). Additionally, looking for differentially expressed genes between all branch and stem samples (Additional file 5), the contrast between both tissues is clear. As well, Additional file 4 exhibited almost the same quantity of differentially expressed and shared genes between both tissues. When plotting stem and branch against non-lignified tissues (flower, seedling, leaf and root) (Additional file 3e-f), still stem exhibited more genes differentially expressed compared to branch. Finally, with DESeq, we obtained 1,502 and 931 differentially expressed genes for stem and branch secondary xylem, respectively, when comparing 12- and 60-year-old trees (Table 1, Fig. 2). The dataset of differentially expressed genes was deposited in NCBI TSA database under TSA study number GDLT00000000. Also, differential expression between branch and stem secondary xylem, stem secondary xylem against non-lignified tissues (leaf, flower, root and seedling) and branch secondary xylem against non-lignified tissues provided 28,022, 14,293 and 10,783 genes, respectively (Fig. 2).

### Functional annotations of unigenes differentially expressed in lignified tissues

**Fig. 3:**
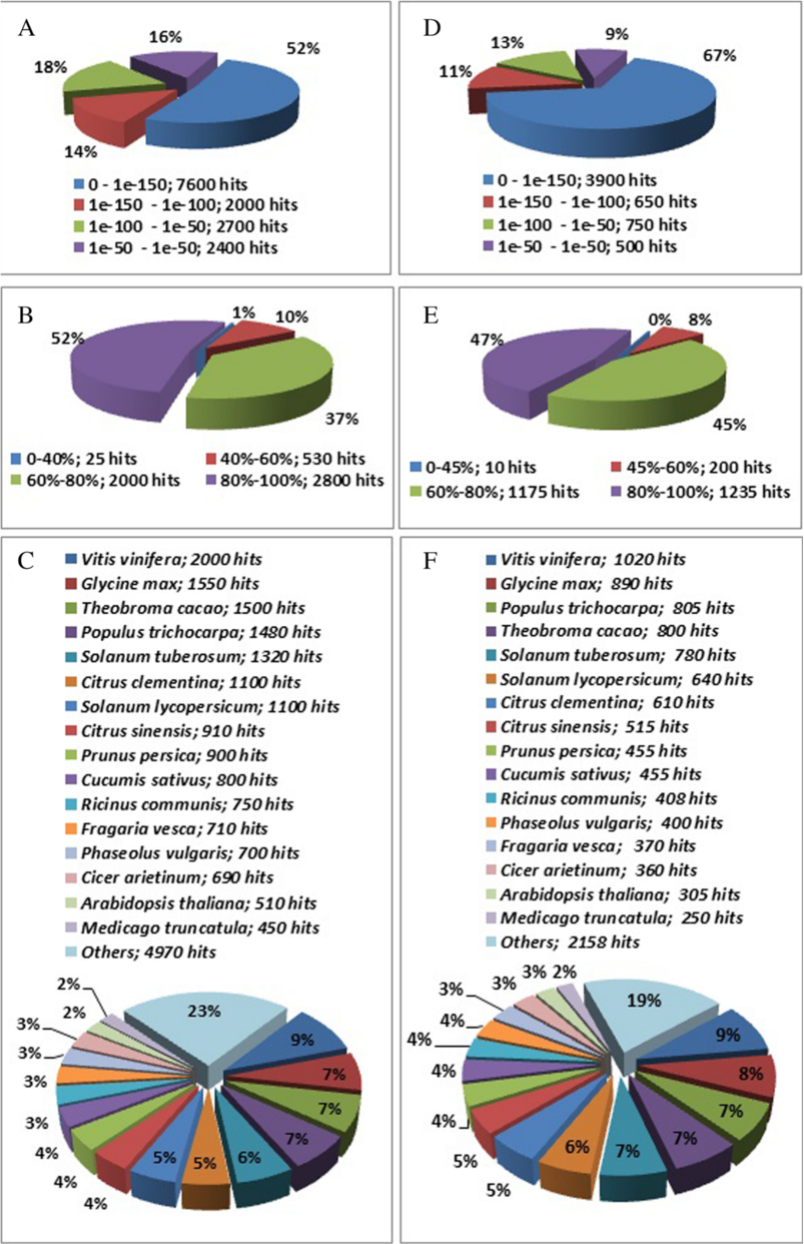
Homology analysis of *T. grandis* differentially expressed unigenes. Branch secondary xylem : **a** E-value distribution. **b**) Similarity distribution. **c**) Species distribution. Stem secondary xylem: **d**) E-value distribution. **e**) Similarity distribution. **f**) Species distribution

**Fig. 4:**
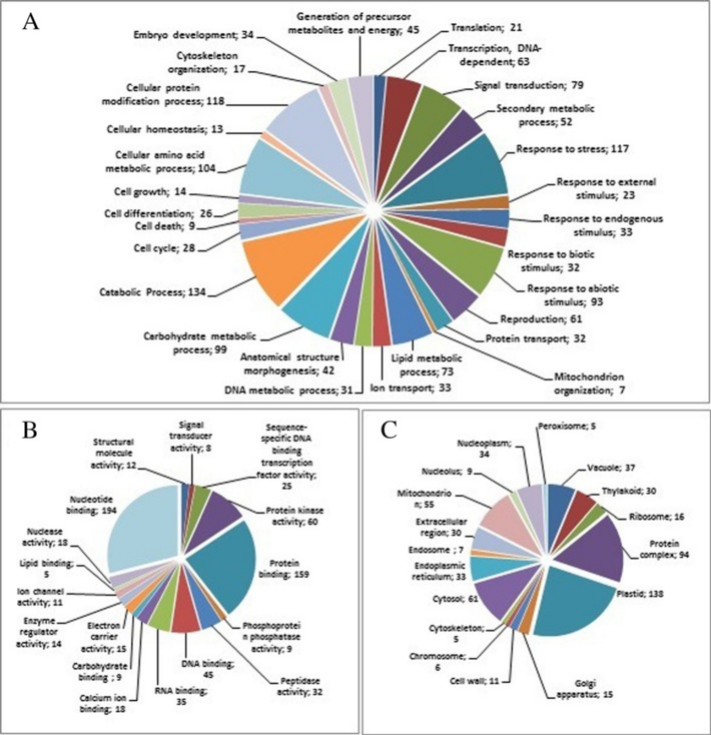
Gene ontology (GO) assignment for the unigenes differentially expressed of *T. grandis* stem secondary xylem. GO assignments (multilevel pie chart with term filter value 5) as predicted for **a** biological process, (**b**) molecular function and (**c**) cellular components. The number of unigenes assigned to each GO term is shown behind semicolon

From the 1,502 and 931 differentially expressed transcripts for stem and branch secondary xylem, respectively (Fig. 2), an annotation of 669 (44.5 %) and 603 (65 %) genes was achieved with a known function by Blast2Go, respectively (Table 1). Among the 669 genes annotated for stem secondary xylem, 48 % (Fig. 3a) exhibited strong homology (E-value smaller than 1e-50). Also, for the same tissue, the similarity distribution showed that 89 % of the genes have more than 60 % identity with other plants (Fig. 3b) and for the species distribution, *T. grandis* had the greatest number of matches with *Vitis vinifera*, followed by *Glycine max*, *Theobroma cacao* and *Populus trichocarpa* (Fig. 3c and 3f). On the other hand, from the 603 genes annotated for branch secondary xylem, 33 % (Fig. 3d) revealed an homology with e-value smaller than 1e-50, and in the identity comparison showed that 92 % of the genes have more than 60 % identity with other plants (Fig. 3e). Most of the differentially expressed genes had a size between 1,000 and 4,000 bp (Additional file 6 and Additional file 7). Gene ontology (GO) tool classified the unigenes in several sub-categories for biological process, cellular component and molecular function. In stem secondary xylem (Fig. 4), catabolic process (9 %), cellular protein modification process (8 %), response to stress (8 %) and carbohydrate metabolic process represented the most abundant sub-categories in the biological process category (Fig. 4a), indicating the expression of genes related to catabolic activities and stress, where several heat-shock proteins were found. Under the molecular function category, the top 2 sub-categories were nucleotide and protein binding (29 % and 24 %, respectively) (Fig. 4b), where three *R2R3-MYBs* and one *CC*-*MYB* transcription factors were found and used for subsequent analysis. In the cellular component category, plastid (21 %) and protein complex (14 %) were the most abundant (Fig. 4c). In branch secondary xylem (Additional file 8), all categories showed similar results to stem secondary xylem (Additional file 9), except for the protein transport through plasma membrane function. Catabolic process and response to stress (biological process), nucleotide and protein binding (molecular function), plastid and plasma membrane (cellular component) are the main categories for both tissues (Additional file 9). Further, three heat-shock proteins (*TgHsp1, TgHsp2* and *TgHsp3*), one *carboxylesterase* (*TgCES*) and one *bax inhibitor* (*TgBi*) with significant up-regulation were found in stem secondary xylem (Additional file 10, Additional file 11), and subsequent expression analyzes of these genes were performed.

### Metabolic pathways of unigenes

**Fig. 5:**
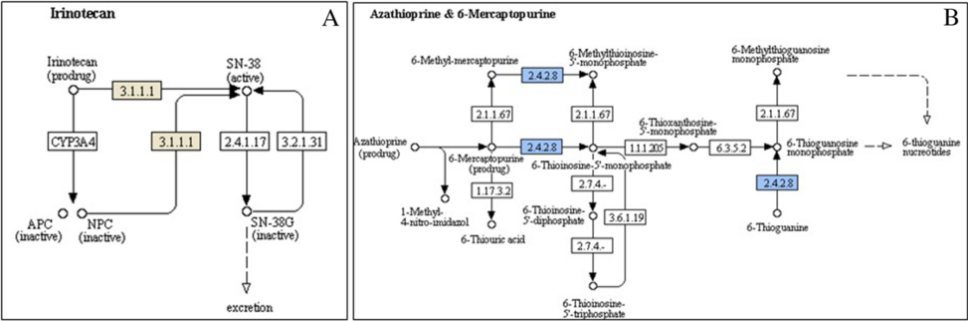
**a** Irinotecan metabolism with the teak ali-esterase enzyme in brown (EC 3.1.1.1). (**b**) Azathioprine-mercaptopurine metabolism with the teak phosphoribosyltransferase enzyme in blue (EC 2.4.2.8)

Beyond finding transcription factors, heat-shock proteins and annotating genes from secondary xylem from teak, we searched for pathways related to those differentially expressed genes. For branch secondary xylem between both ages, 57 paths were identified in the annotated genes (Additional file 12), the most relevant of which, due to number of sequences, were starch and sucrose, amino sugar and purine metabolism. In the case of stem secondary xylem between both ages, 88 metabolic pathways were identified for all annotated differentially expressed genes (Additional file 13). Starch and sucrose, glycerol lipid and purine metabolism presented the highest number of sequences. Also, some relevant metabolisms were found (Additional file 14), such as irinotecan (Fig. 5a) and azathioprine-mercaptopurine metabolisms (Fig. 5b), with the genes located inside the pathway. The *ali-esterase* (Fig. 5a) (which produces the irinotecan) has 3,050 bp. Another relevant gene obtained from the gene ontologies and metabolic pathways is the *beta-galactosidase 17-like* involved in glycan degradation (4,591 bp) (Additional file 14).

### Clustering analysis of the teak R2R3-MYB gene family members

**Fig. 6:**
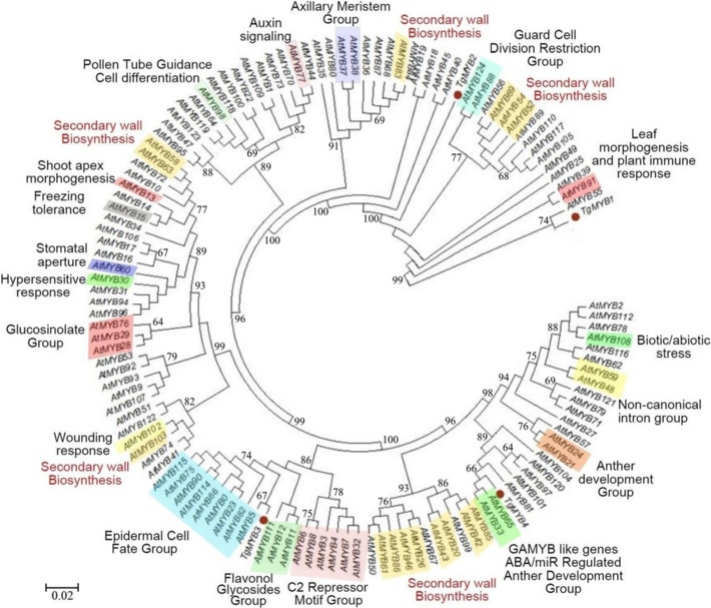
Integrated dendrogram of the 126 *Arabidopsis* R2R3 MYB proteins with teak MYB proteins. Consensus circular tree was conducted by neighbor-joining method and 10000 bootstraps using Mega6 software. Teak MYB proteins are denoted with red dots. Each functional group is colored. References for *MYB* gene functions are defined by previous reports [31, 32, 37]

**Fig. 7:**
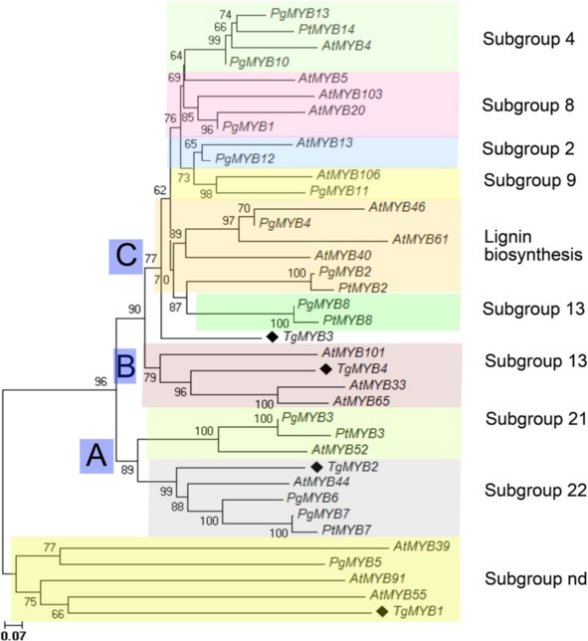
Dendrogram of gymnosperm and angiosperm R2R3-MYB proteins. The neighbor-joining method was used using 10000 bootstraps with several spruce, pine, *Arabidopsis* and teak protein MYB sequences. Teak MYB proteins are denoted with a diamond. The bar indicates the evolutionary distance of 0.2 %. *Arabidopsis* proteins were chosen as landmarks indicating the three main groups (circles **a**, **b** and **c**) and subgroups (Sg next to bracket; nd, not determined) defined by [28]

In order to find phylogenetic relationships between R2R3-MYB members of different plant species and teak, we performed clustering analysis. Indeed, TgMYB1 protein showed a predicted coiled-coil (CC) domain (*MYB-CC* family) (Additional file 15), a subtype within the *MYB* superfamily, as defined by [36]. TgMYB2*,* TgMYB3 and TgMYB4 were consistent with the consensus DNA-binding domain sequences (DBDs) defined for *R2R3-MYB* family, finding R2R3 motifs similar to those found in Arabidopsis, gymnosperm and angiosperm plants [28]. TgMYB2, TgMYB3 and TgMYB4 presented the WTx1EEDx2Lx3Vx4Gx6W and the Rx4Cx1LRWx3Lx1P conserved motifs within the R2 region (Additional file 15). TgMYB2 and TgMYB4 presented the Tx2EEx2LIx2Hx3GNKW motif, TgMYB3 presented the bHLH protein-binding motif ([DE]Lx2[RK]x3Lx6Lx3R) and TgMYB2, TgMYB3 and TgMYB4 presented the PGRx2Nx1IKx2WN motif, all in the R3 region (Additional file 15). Using the complete R2R3-MYB family from Arabidopsis, a dendrogram was obtained to elucidate functional grouping which could also be present in the teak MYB family (Fig. 6). TgMYB3 is located in the epidermal cell fate group, and closely-related to the flavonol glycosides group and C2 repressor motif group, the members of which participate in bHLH interactions and promoter repression [37]. TgMYB4 is inside the GAMYB-like genes group, which are microRNA-regulated genes that facilitate anther development [38]. Additionally, TgMYB1 seems to share a common ancestor with AtMYB55, which do not have related function yet. However, it is unclear how both proteins are grouped, one being CC-MYB (TgMYB1) and R2R3-MYB (TgMYB55) type. Furthermore, using gymnosperm and angiosperm protein sequences to characterize teak MYBs transcription factors, we schemed the three major groups (A, B, C) and subgroups (2, 4, 8, 9, 13, 21, 22) of R2R3-MYBs as described by Bedon et al. (2007). Therefore, TgMYB2 fell into group A, subgroup 22 (pine and spruce MYB7, pine MYB6, MYB9, and AtMYB44) (Fig. 7), which presents motifs involved in protein or DNA interactions. Also, TgMYB2 is close to subgroup 21 (PgMYB3, PtMYB3, and secondary wall biosynthesis AtMYB52), consistent with Fig. 6. Indeed, TgMYB2 could be related with cell wall formation. TgMYB4 is found in group B, subgroup 13 (AtMYB33, AtMYB65, and AtMYB101) (Fig. 7), similar clustering when using all Arabidopsis MYB transcription factors (Fig. 6). Group B was previously described as being present only in angiosperms [28]. TgMYB3 is presented as a separate unit and located inside group C. Group C is also composed by subgroups 2, 4, 8, 9, 13 and lignin biosynthesis sequences AtMYB40, AtMYB46, AtMYB61, PgMYB4, PgMYB2, and PtMYB2. TgMYB1 is still apart from the R2R3 MYB proteins, being clustered with AtMYB55, AtMYB91 and AtMYB39 (Fig. 7), as found in the Arabidopsis grouping (Fig. 6), as expected. Altogether, although R2R3 motifs have several differences in *T. grandis* sequences, they grouped closely to secondary wall biosynthesis genes from other species.

### Gene expression of *MYB* transcription factors in teak

**Fig. 8:**
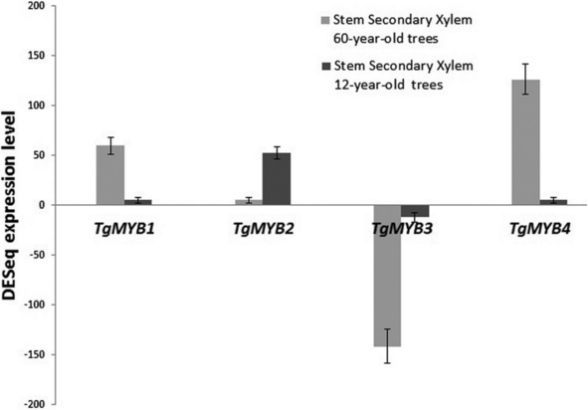
Expression patterns of four *MYB* transcription factors with the DESeq method. We chose four *MYB* transcription factors from the differentially expressed unigenes obtained when comparing stem secondary xylem from mature and young trees. ± means SE of two biological replicate samples were included. The fold changes of the genes were calculated as the log2 value

**Fig. 9:**
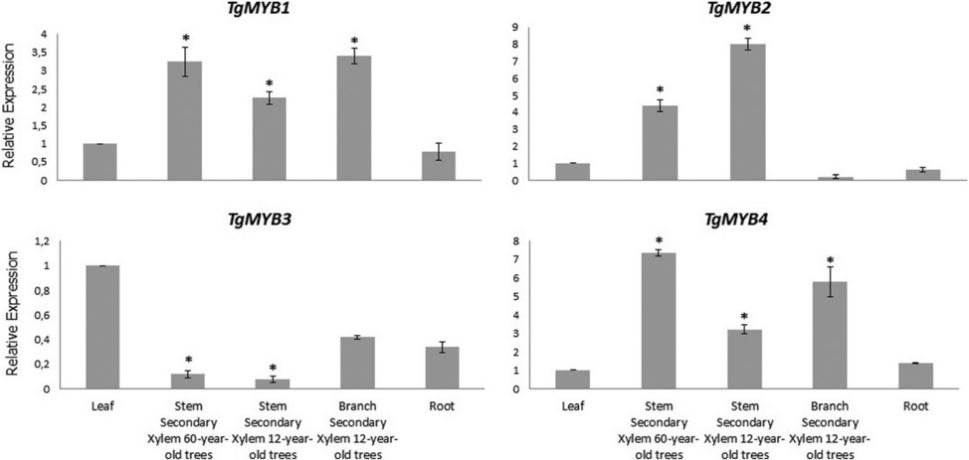
Expression of teak *MYB* genes with the qRT-PCR method. Relative quantification of expression was examined in different tissues (leaf, root, stem and branch secondary xylem from different ages). The name of each gene is indicated at the top of each histogram. Tissues considered are shown at the bottom of the diagrams. ± means SE of three biological replicate samples. *p < 0.05 according to F-test. Y-axis indicates the relative expression level of each gene compared to the control tissue (leaves). *EF1α* was the endogenous control used according to [95]

**Fig. 10:**
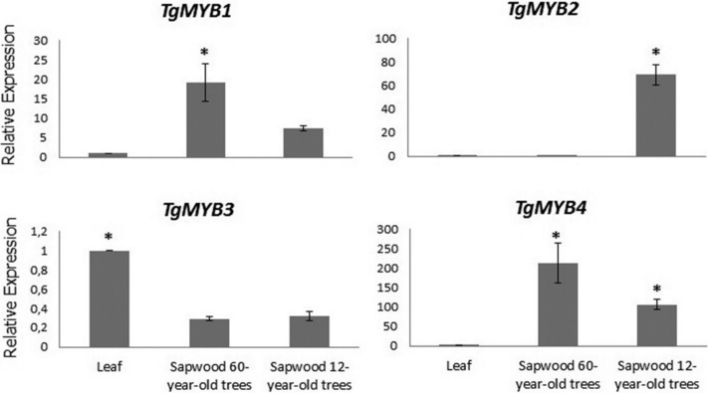
Relative expression levels of teak *MYB* genes in sapwood with the qRT-PCR method. The name of each gene is indicated at the top of each histogram. Tissues considered are shown at the bottom of the diagrams. ± means SE of three biological replicate samples. *p < 0.05 according to F-test. Y-axis indicates the relative expression level of each gene compared to the control tissue (leaves). *EF1α* was the endogenous control used according to [95]

Quantitative real-time PCR analysis showed that four teak *MYBs* are differentially expressed in lignified tissues, being *TgMYB1*, *TgMYB2, TgMYB4* up-regulated and *TgMYB3* down-regulated (Figs. 8–9). In leaves and roots, *TgMYB1*, *TgMYB2* and *TgMYB4* showed almost no expression levels compared to lignified tissues. *TgMYB3* was expressed much higher in leaves than the other tissues, and stem secondary xylem of both ages is shown as down-regulated. The up-regulated genes *TgMYB1* and *TgMYB4* showed comparatively higher expression in stem secondary xylem and sapwood (3-fold and 2-fold, respectively) (Figs. 9–10) in mature (60-years-old) compared to young (12-years-old) trees. Inversely, *TgMYB2* expression is 2-fold higher (Fig. 9) and 60-fold higher (Fig. 10) in stem secondary xylem and sapwood, respectively, of young teak trees. The down-regulated gene *TgMYB3* showed similar expression pattern in stem secondary xylem and sapwood of trees from both ages (Figs. 9–10), although in the DESeq expression level stem secondary xylem from 60-year-old trees showed almost 150-fold less expression compared to 12-year-old trees. Branch secondary xylem of 12-year-old trees seems to have considerable expression levels in *TgMYB1* and *TgMYB4* genes compared to leaves (3- and 6- fold, respectively), but similar expression compared to stem secondary xylem at both ages, with a 95 % statistical confidence level. These results confirm that the unigenes obtained from the transcriptome assembly were differentially expressed, with differences between both ages (Fig. 9). Moreover, the real-time PCR is in agreement with DESeq results (Fig. 8) for *TgMYB1*, *TgMYB2* and *TgMYB4*. Although *TgMYB3* displayed a down-regulated expression in both methods for all tissues when compared with leaf, this gene showed a discrepancy for secondary xylem down-regulated expression at both ages due to the differences of the methods. Overall, the RNA-seq data was biologically validated by the quantitative real-time PCR analysis.

### Gene expression of heat-shock proteins, *carboxylesterase* and *bax inhibitor* transcripts in teak

**Fig. 11:**
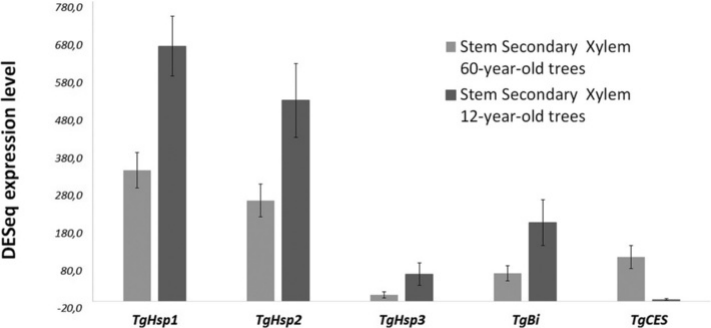
Expression patterns of three heat-shock proteins and two enzymatic genes with the DESeq method. We chose three heat-shock proteins (*TgHsp1, TgHsp2, TgHsp3)*, a *carboxylesterase* (*TgCES*) and a *bax inhibitor* (*TgBi*) from the differentially expressed unigenes obtained when comparing stem secondary xylem from mature and young trees. ± means SE of two biological replicate samples were included. The fold changes of the genes were calculated as the log2 value

**Fig. 12:**
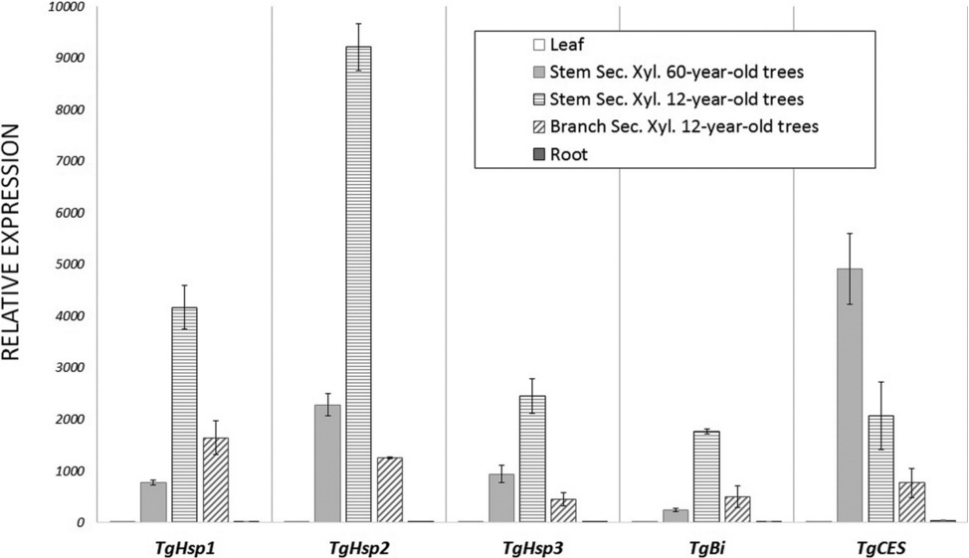
Expression of *TgHsp1, TgHsp2, TgHsp3, TgCES, TgBi* genes with the qRT-PCR method. Relative quantification of expression was examined in different tissues (leaf, root, stem and branch secondary xylem from different ages). The name of each gene, values and tissues considered are shown at the bottom of the diagrams. ± means SE of three biological replicate samples. Y-axis indicates the relative expression level of each gene compared to the control tissue (leaves). *EF1α* was the endogenous control used according to [95]

Expression analysis (by DEseq and quantitative real-time PCR) presented *TgHsp1, TgHsp2, TgHsp3, TgBi* and *TgCES* as differentially expressed transcripts, being up-regulated in lignified tissues (Figs. 11–12). All five genes presented almost null expression in leaves and roots compared to secondary xylem of stem and branch, and all the genes presented more expression in stem compared to branch secondary xylem (Fig. 12). *TgHsp1, TgHsp2, TgHsp3* and *TgBi* showed higher expression in stem secondary xylem of 12-year-old trees compared to 60-year-old trees, with 2-fold, 2-fold, 4-fold and 3-fold more transcripts by DESeq method, respectively (Fig. 11), and 5-fold, 4-fold, 3-fold and 7-fold more expression by qRT-PCR method, respectively (Fig. 12). In contrast to these results, *TgCES* exposed more gene expression in mature teaks (60-years-old) compared to young trees. Again, the quantitative real-time PCR results are similar to the DESeq expression tendencies.

## Discussion

### *T. grandis* transcriptome

The high sensitivity of sequencing technologies presents the RNA-Seq as the preferred choice for transcriptome studies [39], widely replacing the microarray-based gene expression technology [40, 41], the sequencing of cDNA libraries, the SAGE and SuperSAGE analysis [20]. Despite the forestry and economic importance of *T. grandis* around the world, it is very poorly characterized, with only 134 gene sequences deposited in Genbank (access 31/03/2015), most of them being alleles used for molecular markers [8–10, 12, 42–44]. Also, previous genetic studies have focused on proteomic analysis and kinetics of *T. grandis* [45–48]. In this study, we have generated more than 192 million sequence reads (100 bp) corresponding to 38.6 Gigabases of raw sequence data from several tissues (Table 1). *T. grandis* without a sequenced genome and a lack of a sequenced genome in the Lamiales order makes analysis of the teak RNAseq dataset more difficult. *Tectona grandis* is a diploid species with 2n = 36 chromosomes [49]. Ohri & Kumar (1986) [50] estimated the size of its genome by cytogenetic studies, finding about 465 Mbp (1C = 0.48 pg), which is about the same and 2-fold larger than the genome of *Populus trichocarpa* and *Arabidopsis thaliana*, respectively. *A. thaliana* has at least 1,533 transcription factor genes (approximately 6 % of the coding capacity of its genome) [51]. Assuming a similar proportion of transcription for *T. grandis*, all the transcription factors could be estimated in 27.9 Mbp. Comparatively, 270 million reads were obtained from *Phaseolus vulgaris* [52], 71 million reads were generated from stem-root of *Piper nigrum* [20], 59 million reads were generated from *Vitis vinifera* [39], 42 million reads were obtained in *Camellia sinensis* [53] and close to 20 million reads were obtained from *Petroselinum crispum* [54] and *Isatis indigotica* [55]. In eucalyptus, pyrosequencing gave 1.1 million reads [56]. In that sense, Trinity appears as a good choice to assemble de novo full-length transcripts for species without reference genome [57] because it corrects almost 99 % of the sequencing errors. Trinity is a strategy which assembles a set of unique sequences from reads aided by the creation of independent de Bruijn graphs, each representing one group of sequences and assembles isoforms within the groups, running in parallel in a computational cluster [58, 59]. We obtained four different transcriptomes from all tissues using the Trinity platform (Table 1, Additional file 3). Recent studies found 33,238 unigenes in *Isatis indigotica* [55], 62,828 unigenes from *Phaseolus vulgaris* representing 49 Mb [52], 50,161 unigenes from *Petroselinum crispum* [54] and 60,000 unigenes in *Camellia sinensis* [53]. Several trees have generated significantly higher numbers of genes, such as *Salix matsudana* with 106,403 unigenes [60], *Populus trichocarpa* with 36,000 unigenes [15], *Populus euphratica* with 86,777 unigenes [25] and *Fraxinus spp.* with 58,673 unigenes [30].

### RNAseq provided several useful unigenes differentially expressed in lignified tissues of *T. grandis*

From the transcriptome obtained, we were able to identify differentially expressed genes with DESeq program, obtaining an invaluable gene dataset of lignified tissues of teak. DESeq method is a parametric approach which works with technical replicates, with the variance and mean linked by local regression, and uses the negative binomial distribution (a natural extension of the Poisson distribution) to visualize the intensity-dependent ratio of expression data [61–64]. Our analysis for differentially expressed genes is based in biological replicates, which allow a solid biological interpretation. We found 1,502 and 931 differentially expressed genes in stem and branch secondary xylem, respectively, between young and mature teak trees. Recent studies have shown substantial differences obtaining differentially expressed genes. [20] obtained 22,363 transcripts from stem-root of *Piper nigrum*. In stem, almost 3,000, 8,266 and 1,042 differentially expressed genes were obtained in *Populus trichocarpa* [15], alfalfa [65] and *Brassica juncea* [66], respectively. In eucalyptus, 50,000 contigs were obtained [56] and in *Salix matsudana* 292 miRNA stress-related differentially expressed genes [60]. It is common to find in some treatments no more than 1,000 differentially expressed genes, as the case of *Camellia sinensis* [53]. To compare between two general tissue types that are of interest for woody biomass production [27] such as stem and branch, along with the comparison between young (12-years-old) and mature (60-years-old) trees, we properly performed the differential expression procedure with DESeq program (Additional file 4, Fig. 2). All the differentially expressed genes in both tissues presented high homology (by lower p-values), matched with lignified plants and presented sizes between 1,000-4,000 bp (Fig. 3). After annotations, the catabolic processes, response to stress, carbohydrate metabolism, protein binding, transport and plastid localization were the most abundant sub-categories. These annotations are consistent with biopolymers production, transport, storage and xylogenic-related genes as were found in the transcriptome of *E. grandis* × *E. urophylla* hybrid clone [27], *Picea glauca* [29] and *Populus trichocarpa* [15]. Several differentially expressed genes in the transition between young to mature trees in secondary xylem include glycan degradation cell wall carbohydrate (galactose, starch, sucrose) metabolic genes (Additional file 12 and Additional file 13), *diacylglycerol kinase*, *ali-esterase*, *pectin*-related genes and *galactosyl transferase* (Additional file 10) likely involved in cell wall synthesis and extension, plant defense, cellulose, hemicellulose, lignin and pectin formation were found. In *Pinus taeda* [19, 67] and in aspen [15], several pectin esterases, carbohydrate genes and transcription factors highly expressed in woody tissues were found. Additionally, studies with drought have found differentially expressed genes from cell wall and carbohydrate biosynthetic processes which respond greatly to drought stress and enhance mechanical resistance of drought-exposed cells [52]. Also, several kind of stress in different plants have shown up- and down-regulation of metabolic pathways such as carbon metabolism, sucrose and starch synthesis in maize with drought stress [68]. Both, stem and branch secondary xylem indicated a high proportion of predicted genes localized in plastids and plasma membrane in *T. grandis*, as was found in *P. nigrum* stem [20].

### Relevant biochemical pathways in secondary xylem in *Tectona grandis*

Starch and sucrose metabolism showed highest number of sequences for branch and stem secondary xylem (Additional file 12 and Additional file 13). Traditionally, biomass production has been related with carbon partitioning and source-sink relationships within storage organs when generating sugars and increase ATP for starch synthesis [69]. Understanding the aspects that control the assimilates distribution in plants is still a challenge, but the storage contribution of starch and sucrose from source (leaves) to sink tissues such as secondary xylem [69] is essential for plant support and defense. In the same way, galactosidases in glycan degradation were found in teak secondary xylem (Additional file 14). Galactosidases catalyze carbohydrates, glycolipids and glycoproteins residues in plants, animals and microorganisms [70]. Particularly, *Beta-galactosidase* gene has the ability to degrade cell wall fractions and act on small polysaccharide arrangements which hold galactose [70]. Additionally, stem secondary xylem presented irinotecan and azathioprine metabolisms (Fig. 5), considered important plant derivatives in medical application. Irinotecan is a camptothecin-type metabolite, a plant alkaloid with antitumor properties in human gastrointestinal tract [71]. Azathioprine is an immunosuppressive drug used to treat autoimmune human diseases such as rheumatoid arthritis [72] and to avoid organ rejection after transplant surgeries [73].

### Stimulus response genes and heat-shock proteins

Differentially expressed genes included several stimulus response genes, cell death-associated genes and phenylpropanoid biosynthetic genes (Additional file 12, Additional file 13, Additional file 14). Consequently, three heat-shock proteins (*TgHsp1, TgHsp2* and *TgHsp3*), a *bax inhibitor* (*TgBi*) and a *carboxylesterase* (*TgCES*) genes were found in stem secondary xylem with a noticeable expression by DESeq (Additional file 10, Additional file 11). Then, quantitative real-time PCR confirmed the DESeq analysis, indicating that *TgHsp1, TgHsp2*, *TgHsp3* and *TgBi* are expressed more in stem secondary xylem of 12-year-old trees compared to 60-year-old trees (Figs. 11–12). Particularly, plant *carboxylesterase* gene has been related with fruit ripening [74], but this gene could probably be related with several environmental stimulus in teak and other plants, being necessary to be more elucidated in future studies. In addition, the *bax inhibitor* homologs exist in multiple eukaryotic species and translate a multi-membrane-spanning protein to provide cytoprotection against diverse stimuli and stresses, especially with H_2_O_2−_ induced cell death downstream of reactive oxygen species (ROS) signaling [75, 76]. Given that *bax inhibitor* gene in plants is related with enhanced stress tolerance and cell death suppression, it may be linked to cell death regulation in lignified tissues of *Tectona grandis*. In *Capsicum annum*, *bax inhibitor* gene expression was induced by drought, ABA, high salinity, flooding, heavy metal stresses and high or low temperatures [77], which means a substantial role of tolerance to several types of environmental stresses. Also, transgenic cells overexpressing *AtBI-1* showed enhanced tolerance to cell death induced by various oxidative stress, such as H_2_O_2,_ salicylic acid and pathogen elicitor [76]. Similar to our results, during ecodormancy of *Quercus petraea* several stress-related genes were found, including one heat shock protein (HSP18.2), as one of the most expressed genes among all, which is regulated by ABA [22]. Ecodormancy state occurs when temperatures rise from late winter to early spring to prevent bud burst, so heat shock proteins show chaperone activity in order to maintain the proteins in their functional conformation and prevent degradation and damage during heat stress [22]. Curiously, genes encoding enzymes related to heat stress and heat-shock proteins showed differential expression between climacteric treatments in *Pyrus ussuriensis* fruits [78]. Also, [24] compared regulatory networks between primary and secondary meristems, finding common regulatory mechanisms between both stages. The same authors described several stress-related genes playing a role in protecting the secondary xylem under stress conditions. Occasionally, sucrose synthases and glycosylases show a connection with stress-related genes, playing a role in reconverting sugars with a further transport into the cambial zone [19, 24]. One heat-shock protein acting with cell-wall related genes were reported in *Pinus taeda* [19]. Particularly, *TgHsp1, TgHsp2*, *TgHsp3* and *TgBi* showed in teak young secondary xylem more expression than mature ones (Additional file 11, Figs. 11–12). This suggests elevated rates of protein turnover in younger stages of this tree, as might be expected for actively dividing cells compared to mature tissues (60-years-old).

### *MYB* transcription factors revealed clustering and distinct expression during maturity

Differentially expressed transcription factors during vascular development and secondary growth are of high interest due to the wood’s economic value. Also, they play roles as regulators, controlling response networks and modifying wood and fiber qualities [15]. *MYB* transcription factor family plays a fundamental role in xylem development in different plant species and it is a critical regulator of phenylpropanoid pathway [15] such as *Arabidopsis thaliana* [37, 79–82], maize [83], wheat [84], and trees such as *Picea glauca* [28], *Pinus taeda* [85, 86], Eucalyptus genera [87, 88] and populus genera [15, 67, 89, 90]. GO process annotation in the differentially expressed genes from stem secondary xylem followed by an individual examination and verification of the transcription factors annotated, led to finding four tissue-specific *MYB* transcription factors whose function is linked to teak maturation. To classify and predict the biological role of the four differentially expressed *MYB* transcription factors found in the stem secondary xylem, domain protein sequence was analyzed (Additional file 15) and clustering distances were calculated comparatively with all *MYB* transcription factors from *Arabidopis thaliana* and other trees (Figs. 6–7). In that sense, *TgMYB1* is part of the *MYB-CC* family; *TgMYB2, TgMYB3* and *TgMYB4* are part of the *R2R3-MYB* family with *TgMYB3* displaying the bHLH motif (Additional file 15). Our data show that the DNA-binding domains (DBDs) of *T. grandis* are conserved. However, *TgMYB3* was found in the arabidopsis *MYB* group which participates in bHLH interactions, promoter repression and lignin biosynthesis genes (77 % of bootstrap, Fig. 7), while *TgMYB4* is in the GAMMYB-like group and inside the group “B” which is only present in angiosperms (94 % of bootstrap, Fig. 7). Also, *TgMYB2* is close to secondary wall biosynthesis function and protein or DNA interactions (99 and 100 % of bootstraps, Figs. 6–7). *TgMYB1* is outside the groups and need to be more elucidated. This diversity between *T. grandis*, Arabidopsis and some trees might give different roles in the secondary xylem formation. It has been identified in poplar 297 *MYB* members [15] and 126 *R2R3-MYB* transcription factors in Arabidopsis [37]. But, with the transcript expression levels by DESeq (log2-ratio) and through qRT-PCR analysis of four of the *MYB* transcription factors in *T. grandis,* it was found that *TgMYB1* and *TgMYB4* showed more expression in secondary xylem and sapwood of mature trees than young ones, *TgMYB2* less expression levels in lignified tissues of mature than young trees and *TgMYB3* a down-regulation in secondary xylem and sapwood at both ages. High expression of the Arabidopsis *AtMYB103, AtMYB85, AtMYB52, AtMYB54, AtMYB69, AtMYB42, AtMYB43, AtMYB20*, *AtMYB58, AtMYB63*, *AtMYB75,* as a simplified example, has been associated with secondary wall thickening [31, 32]. In *Picea glauca*, *PgMYB2*, *PgMYB4* and *PgMYB8,* which are proteins inside group C by the clustering analysis (Fig. 7), were expressed in stem and root [28], curiously expressed preferentially in the secondary differentiating xylem of both juvenile and mature trees. The same authors described that some *MYB* genes were highly expressed in apical stem, such as *PgMYB6* and *PgMYB7,* being subgrouped with *TgMYB2* with high statistical support of 99 % (Subgroup 22, Fig. 7). The species used for the cluster analysis obtained in Fig. 7 (*Arabidopsis thaliana, Picea glauca, Populus trichocarpa*) are grouped separately from teak due to a bias in the specimen sampling, using 10.000 repetitions (see Materials and Methods). Indeed, TgMYB3 remains as an orphan unity. In terms of distances, groups A and B present high statistical supports (bootstraps higher than 79 %). In group C is present TgMYB3 with a boostrap value of 77 % and separates this teak protein with the rest of the cluster. Nevertheless, the lignin biosynthesis subgroup shows a bootstrap value of 62 % (Fig. 7), which reflects an unproportional taxon sample density. Indeed, a unique protein group with different functions can be considered. To conclude, the *T. grandis MYB* family structure and expression is not all that divergent from the gymnosperm and small flowering plants, such as *Arabidopsis thaliana.* Even though there is only a 5 % increase in wood density going from 50- to 51-year-old trees compared to trees going from 8- to 9-year-old trees (when teak responds to fertilization and cultural operations in the initial years), [4] speculated that much of the growth characteristics and biological changes related to wood traits (noticed in early ages) should be absent in later years when sapwood gives way to the comparatively stable heartwood. In our results, *TgMYB1* and *TgMYB4* are differentially expressed in secondary xylem, and highly expressed in sapwood of 60-year-old trees compared to young ones, presumably because they are key in conferring some woody properties that 12-year-old sapwood does not have. Likely, *TgMYB1* and *TgMYB4* could explain the transition from sapwood (usually called "baby teak”) to heartwood and they could be clues in enhancing the heartwood content and natural resistance as a genetic character, something desirable for teak producers.

### Implications and perspectives of this study

These results, the first dataset of sequences of the Lamiales order and *Tectona* genus, will open new perspectives for studies of diversity, ecology, breeding and genomic programs aiming to understand deeply the biology of this species. In tropical zones, woody plants go through seasonal cycles with two stages: a growing period when environmental conditions are favorable and a period of non-growth in winter, and these phenological cycles have been shown to be strongly affected by an increase in the temperature, which has an impact on the biological processes [22]. Heat-shock proteins have a crucial role in maintaining the proteins in their functional conformation when temperatures rise, preventing degradation and damage during heat stress, from late winter to early spring [22]. Indeed, heat-shock proteins aid defending *T. grandis* against those environmental changes in the region sampled and need to be studied more, and in different seasons. Similarly, the molecular mechanism underlying regulation of wood formation in tropical forest trees remains poorly understood. Our transcriptomic study reported changes in the accumulation of up-and down-regulated genes through the maturation of *T. grandis*. Among all these genes, nine were chosen, quantified and validated by qRT-PCR. The up-regulation of *TgMYB1*, *TgMYB2* and *TgMYB4* in teak secondary xylem (*TgMYB1* and *TgMYB4* in mature and *TgMYB2* in young trees) may also be triggered by other transcription factors, especially NAC master regulators [29], in response to cell wall thickening, regulation of phenylpropanoid genes, changing environmental conditions prevailing between winter and spring and as a possible response to other biotic and abiotic stimuli. It is important to take into account how the maturation of teak can influence the expression of the *TgMYB1* and *TgMYB4* transcription factors and a decrease of *TgMYB2*, once they are selectively expressed in mature sapwood. The drastic differences in wood quality comparing young to mature trees are well known, and heartwood and sapwood are considered high heritability characters, so they seem to be important features to be included in breeding programs [4], particularly when short rotations, such as the Brazilian ones (20 years) are targeted. Also, the quality of the juvenile wood itself will be an important target for improvement, and this can be assessed at an earlier stage, along with seeking trees that keep up fast juvenile growth speed for more years reducing the rotation age and yielding higher percentage of heartwood [4]. Globally, the current study provides several novel observations: (i) it contributes an extensive transcriptome analysis for a tropical wood with respects to secondary growth; (ii) we achieved transcription (gene expression) disparity from a gradient of young to mature secondary xylem and sapwood, identifying several tissue- and developmental stage-specific genes; (iii) the secondary growth has unique molecular biology processes, which includes DNA interacting proteins, regulators of lignin pathway, multitude of stress-related proteins, peptide transporters, carbohydrate metabolic genes and pectin formation; (iv) our results provide for the first time differentially expressed heat-shock proteins and *MYB* transcription factors in teak (MYB-CC and R2R3-MYB types), contributing to the understanding of the molecular mechanisms in tropical wood, incentives to conduct reverse genetics and plant transformation in *T. grandis*, and they will aid in understanding regulatory networks of wood formation.

## Conclusion

The transcriptome of *T. grandis* was assembled using about 192 million reads without a reference genome. More than 2,000 differentially expressed genes, including highly expressed heat-shock proteins, carbohydrate metabolic genes and *MYB* transcription factors were obtained, with two biological replicates of 12 and 60-year-old trees. Analyses using DESeq revealed that there are transcriptome changes in maturation of teak secondary xylem from 12- to 60-year-old trees, while enriched GO groups for branch and stem secondary xylem were found similar. In addition, this is the first attempt to assemble transcripts and characterize *MYB* transcription factors from secondary xylem of *T. grandis*. Four *MYB* transcription factors were classified and characterized, finding three of them with high expression and one down-regulated in lignified tissues. Expression patterns of three heat-shock proteins, one *carboxylesterase* and a *bax inhibitor* were also obtained, with significant correlation between DESeq and qRT-PCR expression analysis. The understanding of gene function of woody tissues in forest tree species is highly challenging due to the lack of standard tree transformation, also, due to plant size, slow growth and long generation time, which make breeding programs a very long process. In order to contribute to assist selection of highly productive trees, next-generation sequencing has become the closest technology to identify target genes among thousands of candidates. In conclusion, the data obtained can be used in applied and basic science along with biotechnological approaches to improve tropical trees.

## Methods

### Plant material

Removal and discarding of the *T. grandis* bark of the trunk and the outer suberized layer (secondary phloem and vascular cambium) of approximately 1.5 cm thickness was performed, with a subsequent collection of a blade of 5 mm located after removal, taking a heterogeneous tissue which includes priority secondary xylem (Fig. 1e). Usually, cells of the cambial zone have thin cell walls and can be easily removed from the stem [16]. Branch (from the base and recent ones) (Fig. 1f) and secondary xylem on the main stem at DBH (Diameter at Breast Height) (Fig. 1e) were sampled from twelve-years-old and sixty-years-old *T. grandis* trees from an experimental field (lat. 22°42'23''S, long. 47°37'7''W, 650 m above sea level) at “Luiz de Queiroz” College of Agriculture (ESALQ), University of São Paulo, located in Piracicaba, São Paulo State, Brazil. Additionally, seedlings after two weeks of seed germination (Fig. 1a), leaves (Fig. 1b) and roots (Fig. 1c) from two month-old *in vitro* teaks were sampled. Flowers at different stages were collected from the twelve year-old teak trees (Fig. 1d). All tissues/organs were harvested in ten randomized trees (joining five samples as one replicate), immediately frozen by immersion in liquid nitrogen and stored at −80 °C until RNA extraction. For quantitative Real-Time PCR, sapwood from 12- and 60-year-old trees were also collected at the same location, with three replicates, each one coming from five trees, using an increment borer at DBH [91] (Fig. 1g-h), followed by immediate nitrogen immersion and RNA extraction.

### Total RNA extraction and Illumina sequencing

Frozen tissue samples of 1.0 g were weighed and ground into fine powder in liquid nitrogen using a sterilized mortar and pestle. Total RNA was extracted following the protocol standardized by Salzman et al. (1999) [92]. 2 μg of total RNA from each sample were treated with DNAse I (Promega), and the treated samples were analyzed in agarose gels to ensure absence of DNA and no degradation. In addition, PCR control reactions to examine for genomic DNA contamination were performed using total RNA without reverse transcription as template, and negative results (absence of bands) were assessed by electrophoresis on a 1 % (w/v) agarose gel with ethidium bromide staining. The Agilent RNA 6000 n kit (Agilent, Santa Clara, CA) was used to verify the total RNA quality by the RIN factor in a 2100 Bioanalyzer (Agilent, Santa Clara, CA). Then, the TruSeq RNA Sample Prep Kit v2 (Illumina, San Diego, CA) was used to prepare the libraries of all tissues from 1 μg of total RNA, with replicates for stem and branch secondary xylem at both ages. For clustering the libraries, the TruSeq PE Cluster Kit v3-cBot-HS (Illumina, San Diego, CA) was used. To verify the size of the libraries, the Agilent DNA 1000 kit (Illumina, San Diego, CA) was used. For sequencing, the TruSeq SBS Kit v3-HS (Illumina, San Diego, CA) was used, with 200 cycles, using the Illumina HiSeq 1000 (Illumina, San Diego, CA) located at “Luiz de Queiroz” College of Agriculture (ESALQ), University of São Paulo (Brazil).

### Cleaning and de novo assembly

Raw reads of the twelve samples were “trimmed” to increase the quality and further be used in the de novo assembly [34]. The de novo assembly was performed for the twelve samples with the cleaned reads using the Trinity program, version 2013 [35, 57] at the “Ohio Super Computer Center” (OSC), Ohio State University (USA). Then, the reference transcriptome was prepared and RSEM tool was used to estimate abundance of reads for subsequent differential expression.

### Detection and annotations of differentially expressed unigenes between twelve and sixty year-old trees

We used DESeq, an R Bioconductor package [61], to perform the differential expression of unigenes between lignified tissues and the different ages at the “Ohio Super Computer Center” (OSC), Ohio State University, USA. Abundance estimation and FPKM value was obtained using RSEM [35]. Next, two matrixes were generated, one containing the counts of RNA-seq fragments and used for differential expression by DESeq and the other one performing the TMM normalization in order to generate graphics. The lignified groups for comparison were: (1) Branch secondary xylem of 12-year-old trees against Branch secondary xylem of 60-year-old trees, (2) Stem secondary xylem of 12-year-old trees against stem secondary xylem of 60-year-old trees, (3) Branch vs. Stem secondary xylem, (4) Other tissues (flower, leaf, root, seedling) vs. Branch secondary xylem (5) Other tissues (flower, leaf, root, seedling) vs. Stem secondary xylem. The results were represented in “MA” and “volcano” plots from pairwise comparisons using both replicates for branch and stem secondary xylem and a cutoff of false discovery rate (FDR) < =0.05. Subsequently, differentially expressed unigenes were exported with the “cdbfasta” tool (http://compbio.dfci.harvard.edu/) with the contig name from assemblies of Trinity database in .fasta format. The differentially expressed unigenes were annotated using Blast2Go [93]. The parameters in the “GO annotation” were an “E-value hit filter” of 1.0E-6, an “Annotation Cut-Off” of 55 and a “GO-Weight” of 5. Finally, KEGG metabolic pathways were obtained in an organized workflow within the Blast2Go.

### Clustering of *MYB* transcription factors differentially expressed in teak

*MYB* transcription factors with complete coding sequence were selected from the annotated differentially expressed genes of stem secondary xylem. The dendrograms were built with Clustal W amino acid alignments and following the neighbor joining tree method in Mega 6 [94], using 10,000 bootstrap replication for the tree nodes, poisson model, amino acid substitution type, uniform rates and pairwise deletion. The first dendrogram was built using sequences of all 126 *Arabidopsis* R2R3 MYB proteins downloaded from the TAIR *Arabidopsis* genome annotation [31, 32, 37]. The second dendrogram was constructed with several predicted MYB protein sequences from white spruce, loblolly pine and diverse *Arabidopsis* MYB sequences [28].

### Gene expression of *MYBs*, heat-shock proteins, *carboxylesterase* and *bax inhibitor* transcripts along the lignified teak tissues by qRT-PCR

Three cDNA samples were synthesized (using an oligo dT primer) from each tissue (branch, stem secondary xylem and sapwood from twelve- and sixty-years-old *T. grandis* trees, leaves and roots from two month-old *in vitro* teaks). Each replicate came from five trees (see Plant Material), using 1,0 μg of the treated RNA using the SuperScript^TM^ III First-Strand Synthesis System for RT-PCR (Invitrogen) according to the manufacturer’s instructions. cDNA concentration was determined with the Ultrospec 2100 PRO Spectrophotometer (Amersham Biosciences, USA). The primers for qRT-PCR were designed flanking *TgMYB1, TgMYB2, TgMYB3, TgMYB4, TgHsp1, TgHsp2, TgHsp3, TgCES,* and *TgBi* teak sequences (Additional file 16), followed by determining the standard curve with several cDNA dilutions and the melting curve (Additional file 17). The qRT-PCR mixture contained 125 ng of cDNA from each sample, primers to a final concentration of 50 μM each, 12.5 μl of the SYBR Green PCR Master Mix (Applied Biosystems, USA) and PCR-grade water up to a total volume of 25 μl. Each gene reaction was performed in technical replicate. PCR reactions without template were also done as negative controls for each primer pair. The quantitative real-time PCRs were performed employing the StepOnePlus™ System (Applied Biosystems, USA). All PCR reactions were performed under the following conditions: 2 min at 50 °C, 2 min at 95 °C, and 45 cycles of 15 s at 95 °C and 1 min at 65 °C in 96-well optical reaction plates (Applied Biosystems, USA). Leaf sample was used as calibrator to normalize the values between different plates and *EF1α* as control gene, following previous studies in teak [95]. All statistically significant differences between the means were performed in SAS program at 95 % confidence level with the F-test, and the pair comparison procedure was performed with LSD at 95 % confidence level.

### Availability of supporting data

The raw reads were deposited in the “Short Read Archive” (SRA) database at NCBI under accession number SRP059970. The differentially expressed genes were deposited in the “Transcriptome Shotgun Assembly” (TSA) database at NCBI under accession GDLT00000000. The version described in this paper is the first version, GDLT010000. Both raw reads and differentially expressed genes are associated to the Bioproject PRJNA287604 at NCBI. Dendrograms I (Fig. 6) and II (Fig. 7) are available in TreeBASE with the links http://purl.org/phylo/treebase/phylows/study/TB2:S18133 and http://purl.org/phylo/treebase/phylows/study/TB2:S18139, respectively. All selected genes and accession numbers are found in Additional file 18.

## Additional files

Additional file 1:
**RIN factor of all samples used for Illumina sequencing.** (PDF 225 kb) Additional file 2:
**FASTQC reports of each sample from the RNAseq of** ***Tectona grandis. (PDF 2000 kb)***
Additional file 3:
**Raw data, cleaning data and assembly.** (PDF 93 kb) Additional file 4:
**Differential expression of log2 ratio (fold change) versus mean between different conditions with DESeq program.** a) Dispersion plot for branch secondary xylem transcripts. b) Significantly differentially expressed transcripts scatterplot for branch secondary xylem transcripts. c) Dispersion plot for stem secondary xylem transcripts. d) Significantly differentially expressed transcripts scatterplot for stem secondary xylem transcripts. e) Significantly differentially expressed transcripts scatterplot for branch secondary xylem against flower, seedling, leaf and root. f) Significantly differentially expressed transcripts scatterplot for stem secondary xylem against flower, seedling, leaf and root. Fitted curve of the spots is in red. Red dots indicate transcripts differentially expressed at 10 % false discovery rate and black spots transcripts are expressed in common [61]. (PDF 132 kb) Additional file 5:
**Significantly differentially expressed transcripts plot for stem-branch genes.** Red plots indicate transcripts differentially expressed and black spots transcripts expressed in common. (PDF 144 kb) Additional file 6:
**Length and number of sequences for stem differentially expressed genes.** (PDF 122 kb) Additional file 7:
**Length and number of sequences for branch differentially expressed genes.** (PDF 125 kb) Additional file 8:
**Gene ontology (GO) assignment for the unigenes differentially expressed of** ***T. grandis*** **branch secondary xylem.** GO assignments (multilevel pie chart with term filter value 5) as predicted for (a) biological process, (b) molecular function and (c) cellular components. The number of unigenes assigned to each GO term is shown behind semicolon. (PDF 143 kb) Additional file 9:
**GO frequencies for differentially expressed (DE) transcripts.** Stem and Branch secondary xylem were the tissues presented in the table. DE transcripts were obtained when comparing 12- and 60-year-old teak trees in both tissues. The first column for GO frequencies is organized from lowest to highest. (PDF 119 kb) Additional file 10:
**43 genes highly differentially expressed between stem secondary xylem from 12- and 60-year-old trees. **(PDF 106 kb) Additional file 11:
**Other relevant differentially expressed genes from secondary xylem.** We chose other genes with the highest expression between young (12-years-old) and mature (60-years-old) trees, and performed a transformation of root square in order to visualize their values. (PDF 178 kb) Additional file 12:
**Branch secondary xylem pathways found by Kegg.** (PDF 116 kb) Additional file 13:
**Stem secondary xylem pathways found by Kegg.** (PDF 129 kb) Additional file 14:
**Relevant enzymes found for differentially expressed genes in stem.** In Blue, sequences higher than 3000 bp. (PDF 114 kb) Additional file 15:
**Predicted MYB domain protein sequences from** ***Tectona grandis*** **.** Amino acid sequences of the four *MYB* transcription factors were obtained with ExPASy Translate tool (http://web.expasy.org/translate/). Grey shading indicates identical amino acid residues that agree with the motifs referenced by Bedon et al. (2007). MYB-CC type transfactor domain (*TgMYB1*) and R2R-MYB DNA-binding domains (MYBR2R3-DBDs) (*TgMYB2, TgMYB3, TgMYB4*) are indicated. bHLH motif ([DE]L × 2 [RK] × 3 L × 6 L × 3R) is indicated in *TgMYB3. (PDF 131 kb)*
Additional file 16:
**Primers for quantitative real-time PCR.** (PDF 90 kb) Additional file 17:
**Melting curves and efficiencies of primers for quantitative real- time PCR.** (PDF 192 kb) Additional file 18:
**Some transcripts obtained from RNA-seq in** ***Tectona grandis*** **and used for subsequent analysis.** Four *MYB* transcription factors: *TgMYB1* (NCBI Accession number KR092428), *TgMYB2* (NCBI Accession number KR092429), *TgMYB3* (NCBI Accession number KR092430), *TgMYB4* (NCBI Accession number KR092431), three heat-shock proteins*: TgHsp1* (NCBI Accession number KR092432), *TgHsp2* (NCBI Accession number KR092433), *TgHsp3* (NCBI Accession number KR092434), *carboxylesterase*: *TgCES* (NCBI Accession number KR092436), *bax inhibitor*: *TgBi* (NCBI Accession number KR092435). In yellow, the methionine. In green, the stop codon. In grey, the coding sequence. In blue, the real-time PCR primers. (PDF 125 kb)

## Abbreviations

*Tg*: *Tectona grandis*
GB: Gigabases
*MYB*: *MYB* transcription factors
CC: Coiled-coil domain
RIN: RNA integrity number
NCBI: National center for biotechnology information
SRA: Sequence read archive
Bp: Base pairs
Mbp: Mega base pairs
GO: Gene ontology
mm: Millimeters
DBD: DNA-binding domain
bHLH: Basic helix-loop-helix
SAGE: Serial analysis of gene expression
HSP: Heat shock proteins
ABA: Abscisic acid
*Ef-1α*: *Elongation factor 1-α*
DBH: Diameter at breast height
cDNA: Complementary DNA
mRNA: Messenger RNA
PCR: Polymerase chain reaction
qRT-PCR: Quantitative real-time reverse transcription PCR
OSC: Ohio Super computer center
CEBTEC: Centro de biotecnologia agrícola
FPKM: Fragments per kilobase of exon per million fragments mapped
TMM: Trimmed mean of *M*-values
DESeq: R package to analyze count data from RNA-Seq assays and test for differential expression
RSEM: Software package for estimating gene and isoform expression levels from RNA-Seq data
MA plot: An application of a Bland-Altman plot for visual representation of gene expression data transformed onto the M (Log ratios) and A (mean average) scale
FDR: False discovery rate
SAS: Statistical analysis software
LSD: Least significant difference
F-test: Fisher test
