# Supplementary figures and images for: Large-scale transcriptional profiling of lignified tissues in Tectona grandis
Source: BMC Plant Biol. 2015 Sep 15;15:221. doi: 10.1186/s12870-015-0599-x (PMC4570228; doi:10.1186/s12870-015-0599-x)

Additional File 1. RIN factor of all samples used for Illumina sequencing.

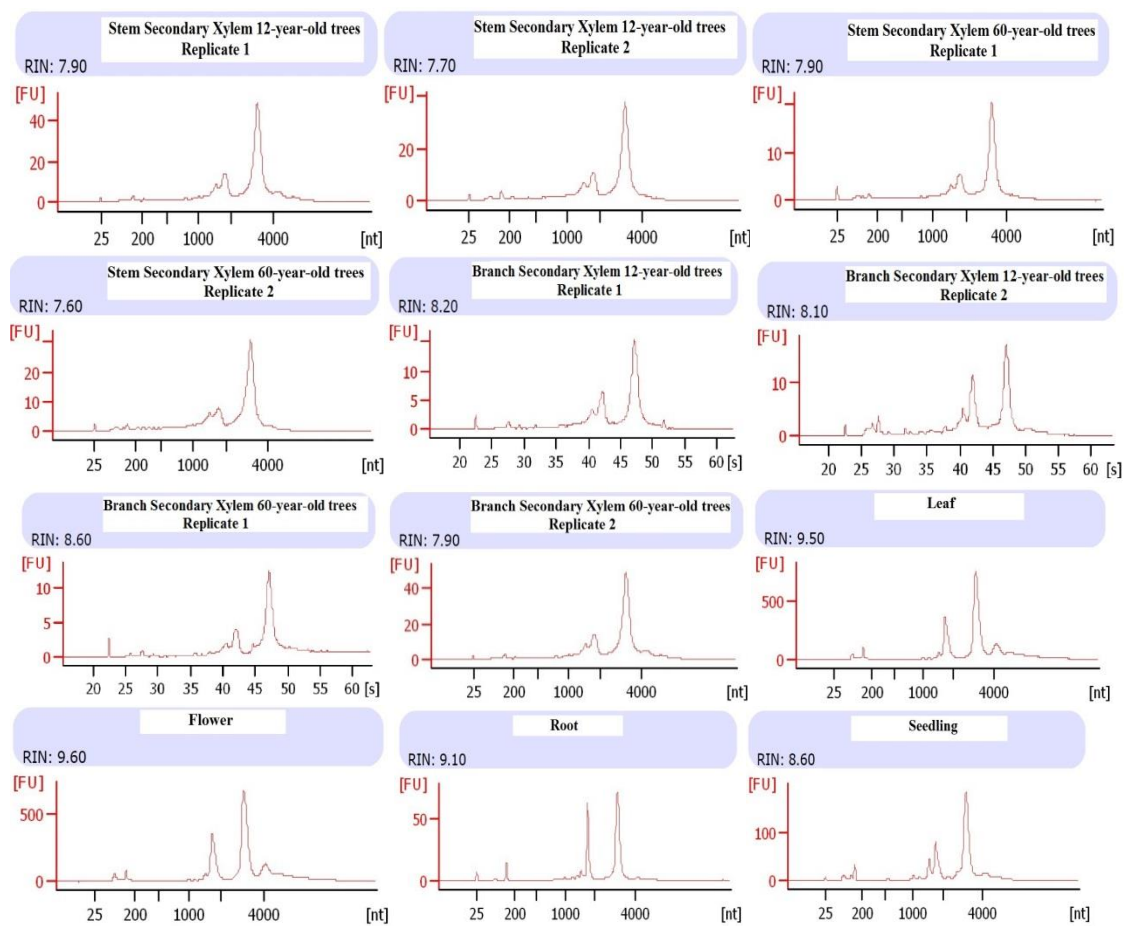

Supplement: Additional file 1: — RIN factor of all samples used for Illumina sequencing. (PDF 225 kb) [file 12870_2015_599_MOESM1_ESM.pdf]

Additional File 6. Length and number of sequences for stem differentially expressed genes.

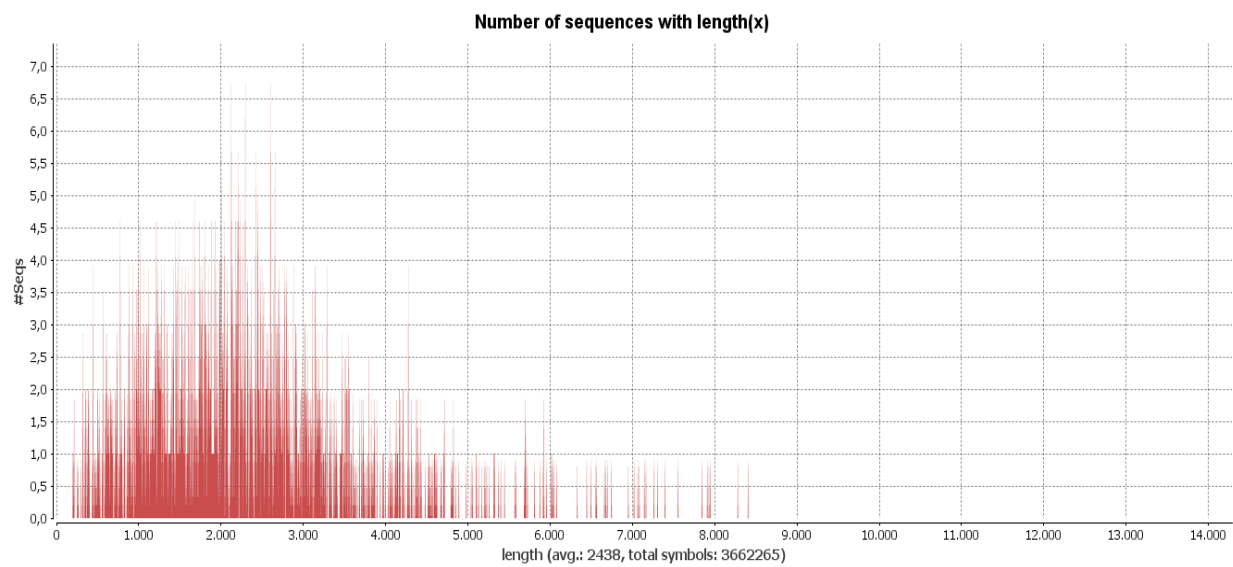

Supplement: Additional file 6: — Length and number of sequences for stem differentially expressed genes. (PDF 122 kb) [file 12870_2015_599_MOESM6_ESM.pdf]

Additional File 7. Length and number of sequences for branch differentially expressed genes.

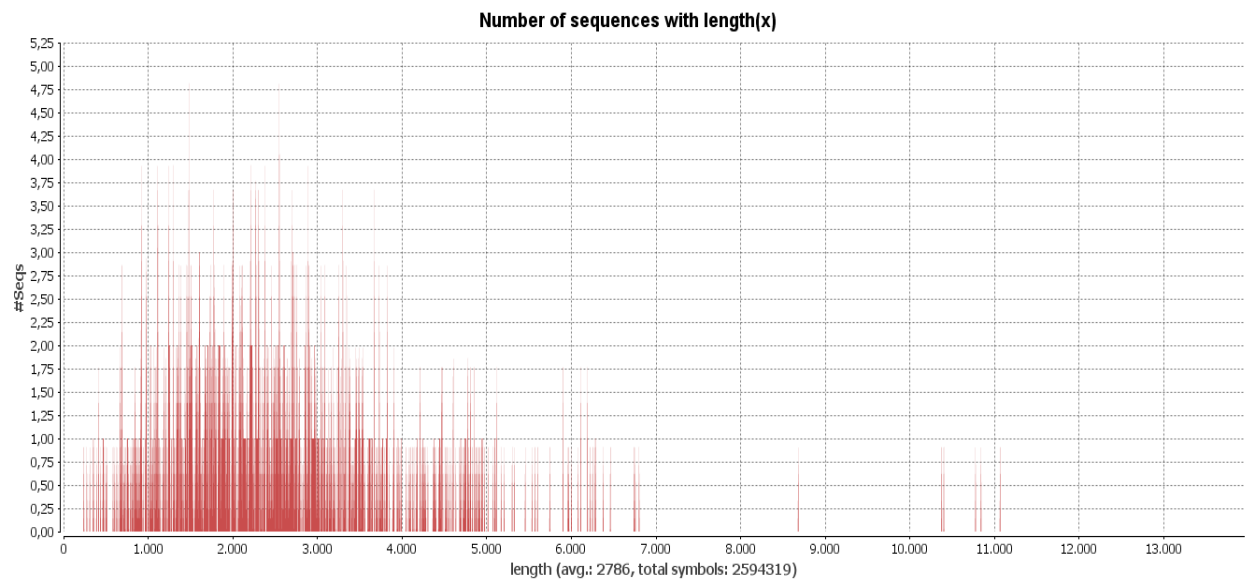

Supplement: Additional file 7: — Length and number of sequences for branch differentially expressed genes. (PDF 125 kb) [file 12870_2015_599_MOESM7_ESM.pdf]

Additional File 17. Melting curves and efficiencies of primers for quantitative real-time PCR.

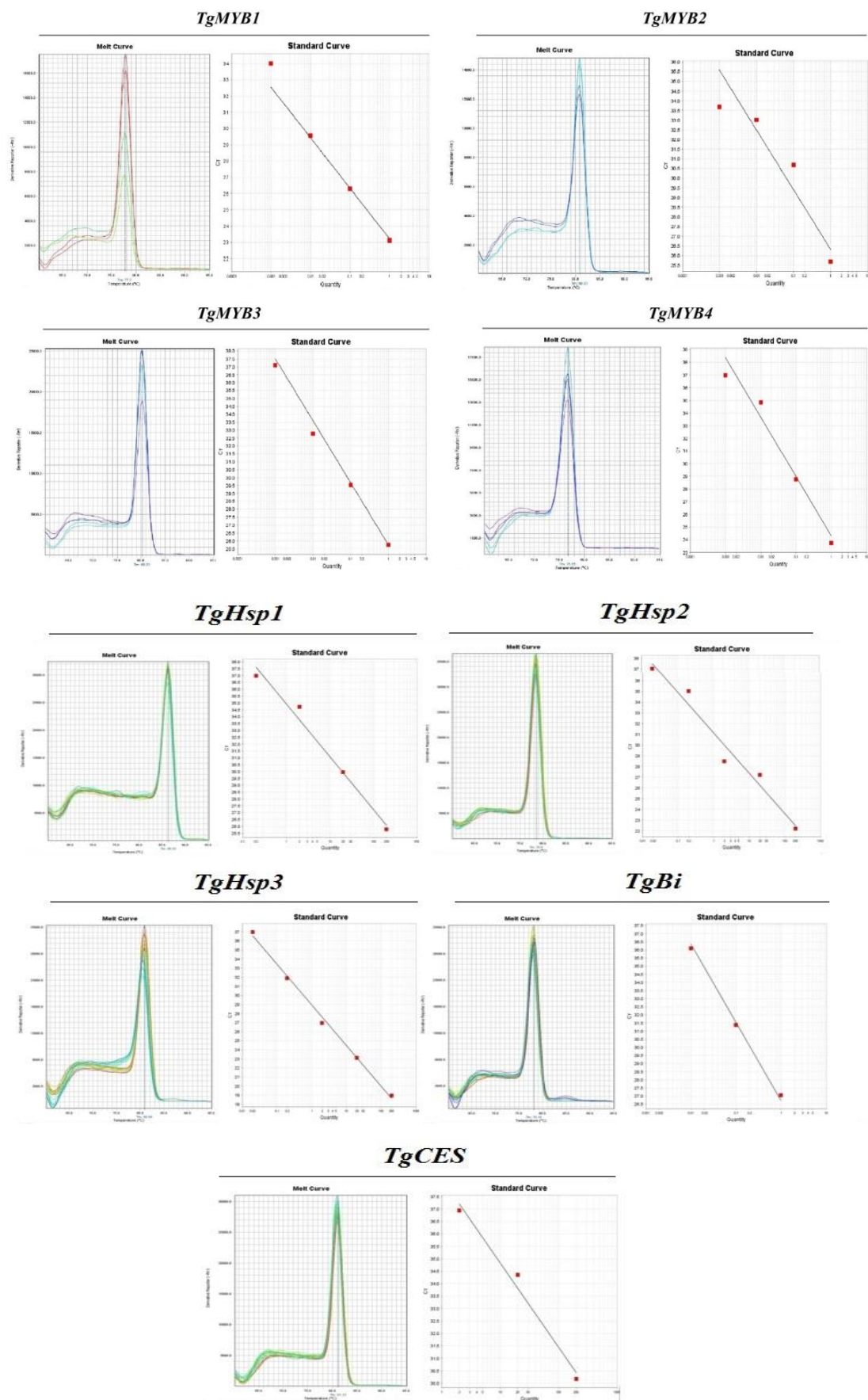

Supplement: Additional file 17: — Melting curves and efficiencies of primers for quantitative real- time PCR. (PDF 192 kb) [file 12870_2015_599_MOESM17_ESM.pdf]
