# Supplementary material for: Large-scale transcriptional profiling of lignified tissues in Tectona grandis
Source: BMC Plant Biol. 2015 Sep 15;15:221. doi: 10.1186/s12870-015-0599-x (PMC4570228; doi:10.1186/s12870-015-0599-x)

Additional File 2. FASTQC reports of each sample from the RNAseq of *Tectona grandis*.

Branch Secondary xylem 12-year-old trees. Repetition 1

Per base quality

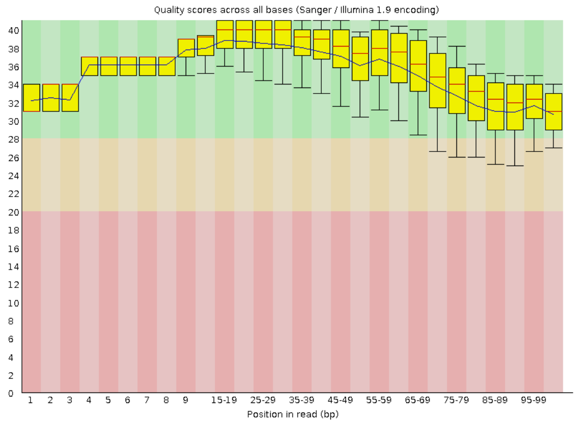

Per base sequence content

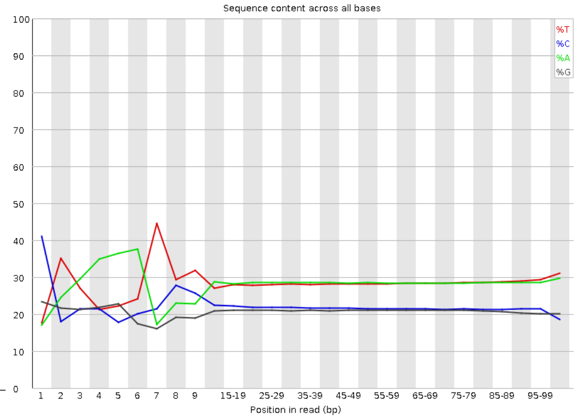

Per sequence GC content

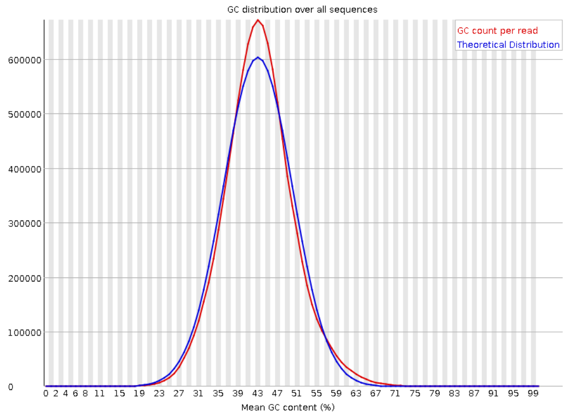

Per sequence quality

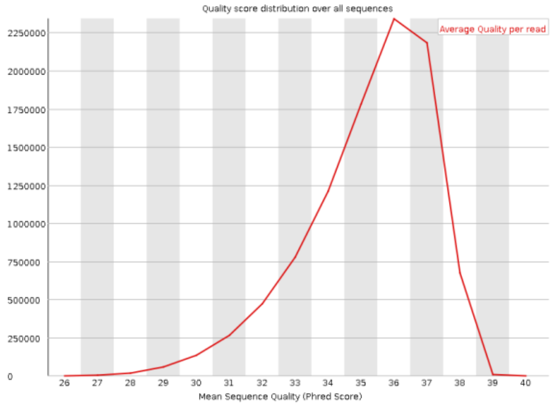

Duplication levels

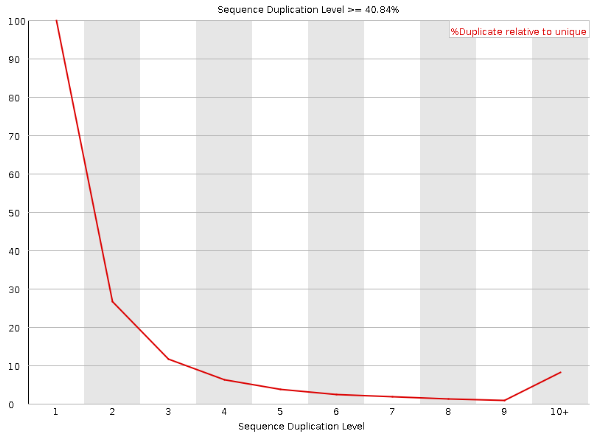

Sequence length distribution

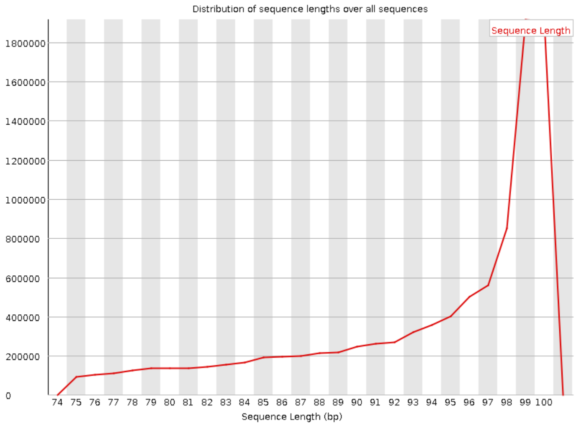

Branch Secondary xylem 12-year-old trees. Repetition 2

Per base quality

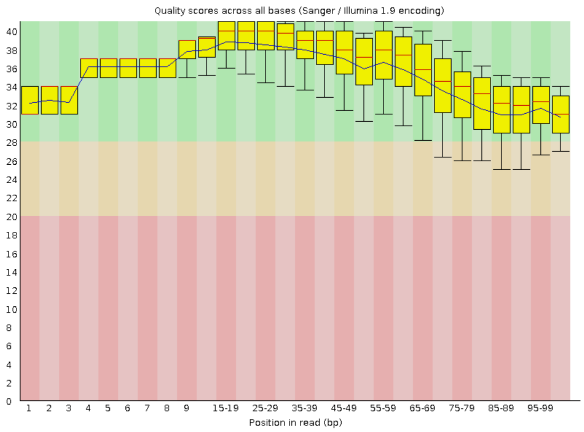

Per base sequence content

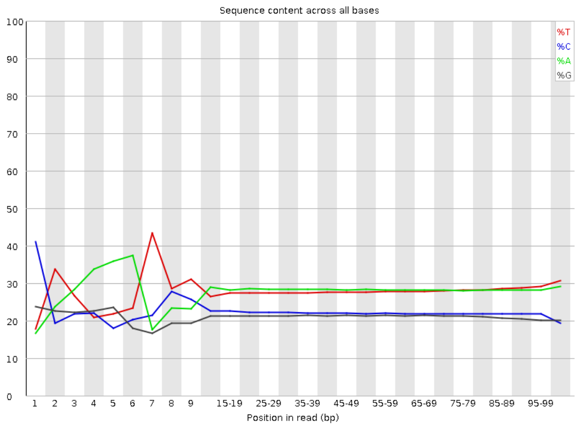

Per sequence GC content

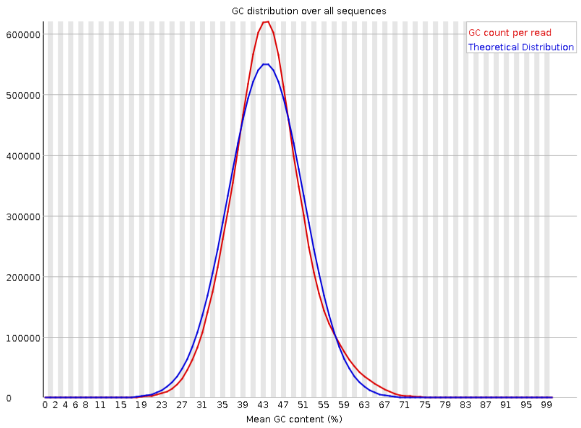

Per sequence quality

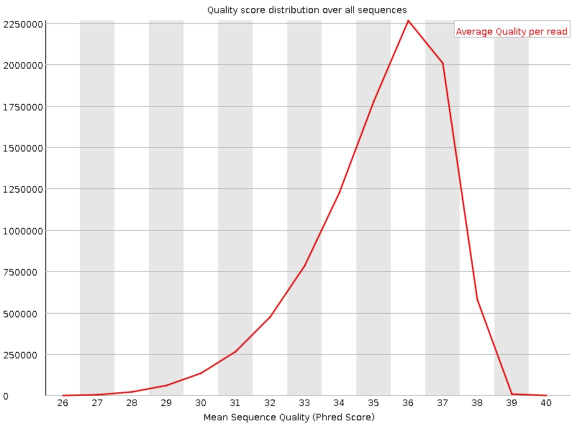

Duplication levels

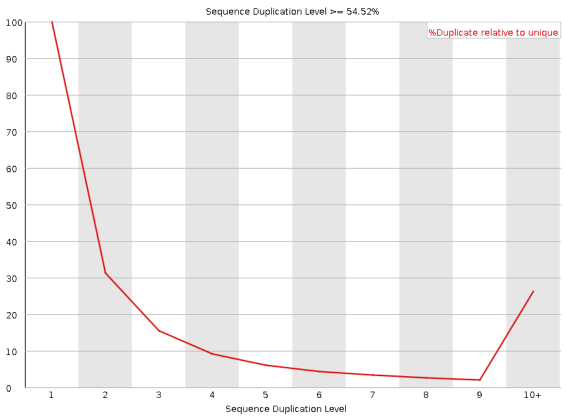

Sequence length distribution

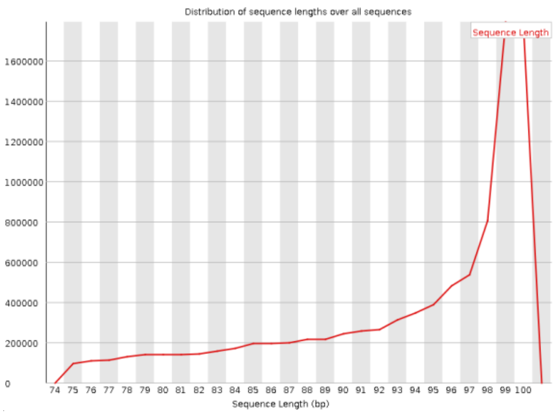

Branch Secondary xylem 60-year-old trees. Repetition 1

Per base quality

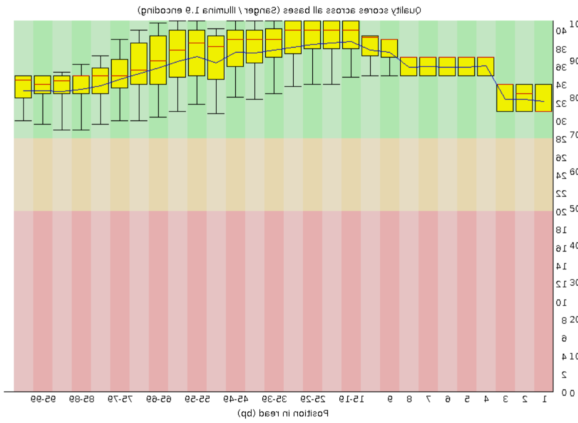

Per base sequence content

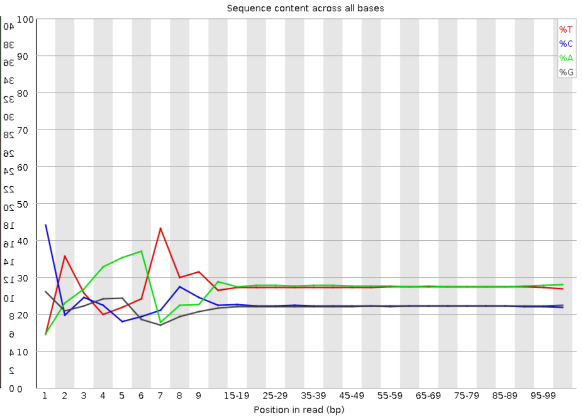

Per sequence GC content

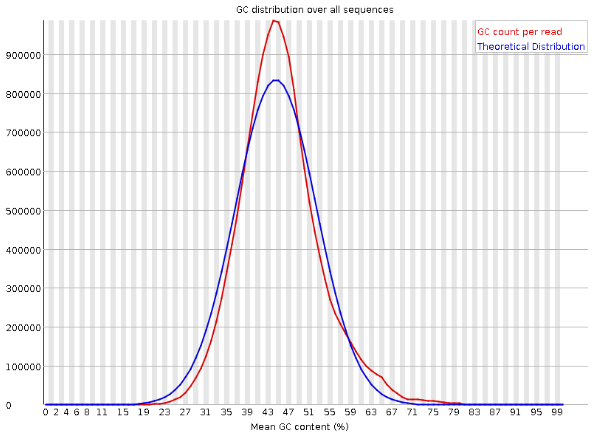

Per sequence quality

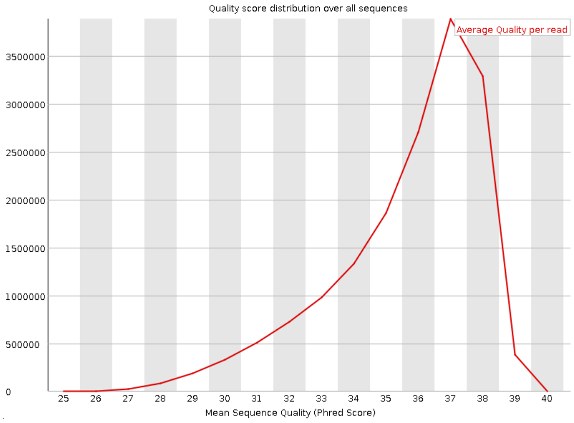

Duplication levels

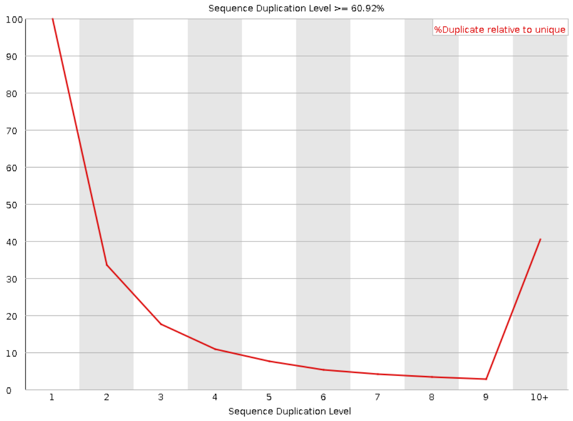

Sequence length distribution

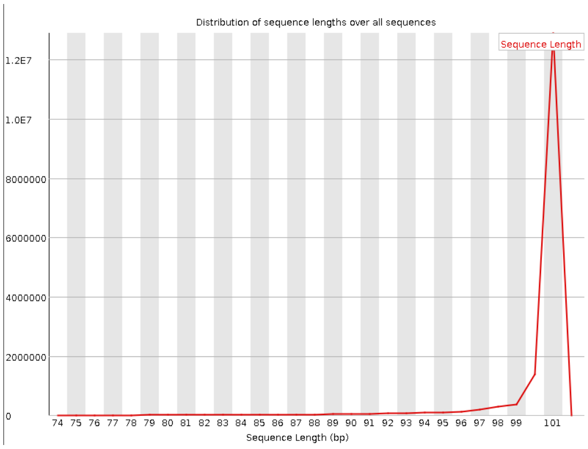

Branch Secondary xylem 60-year-old trees. Repetition 2

Per base quality

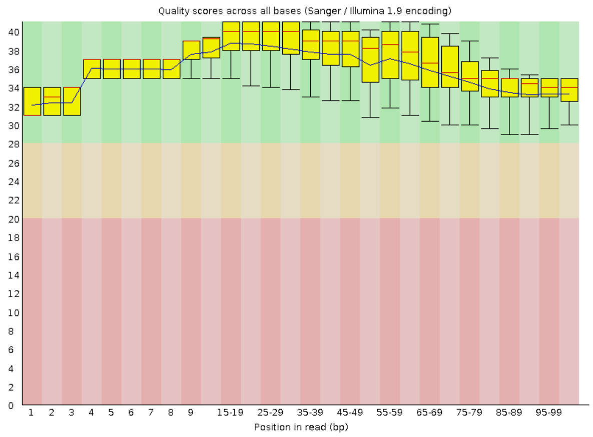

Per base sequence content

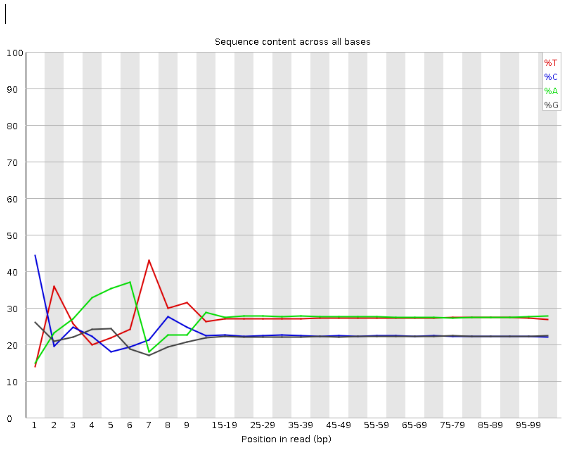

Per sequence GC content

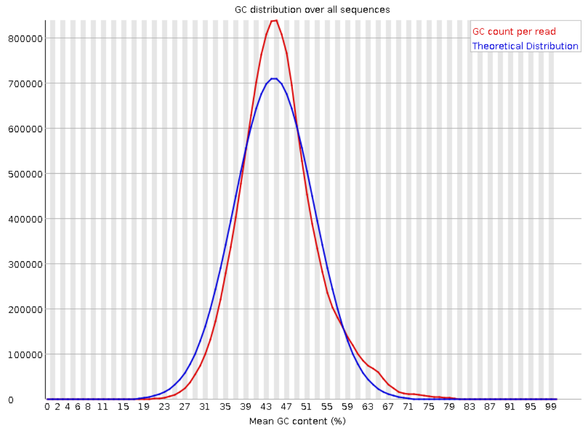

Per sequence quality

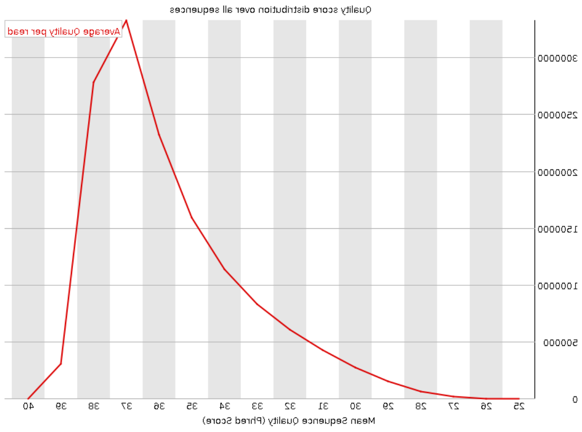

Duplication levels

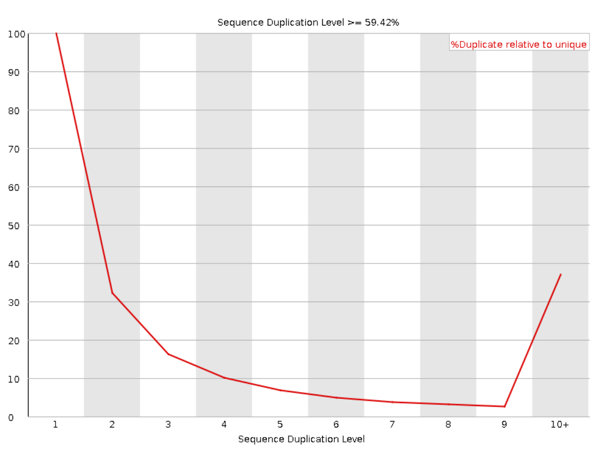

Sequence length distribution

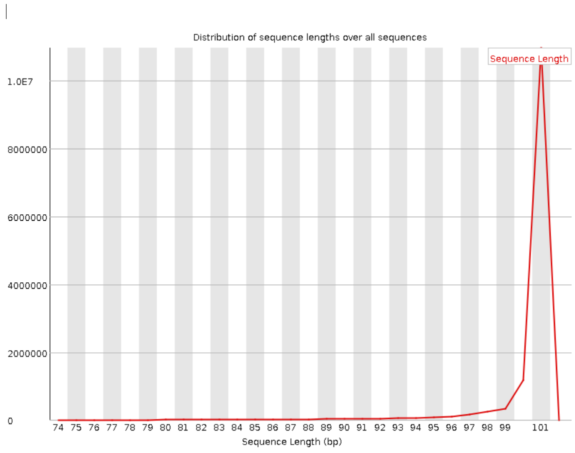

Stem Secondary xylem 12-year-old trees. Repetition 1

Per base quality

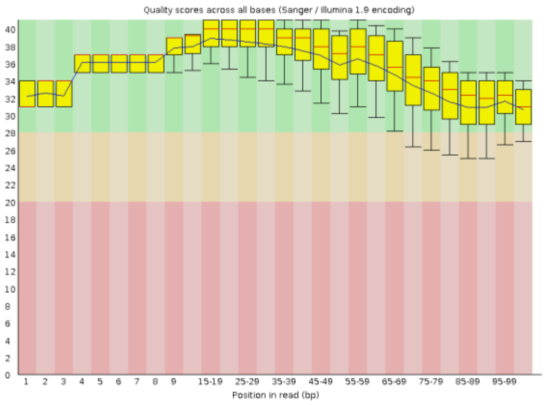

Per base sequence content

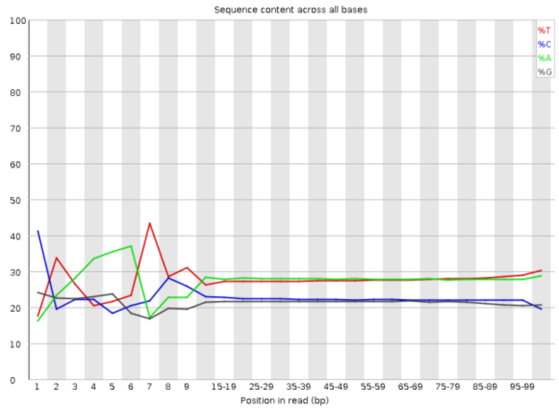

Per sequence GC content

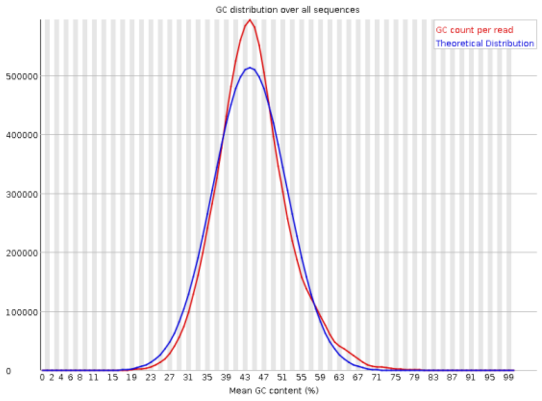

Per sequence quality

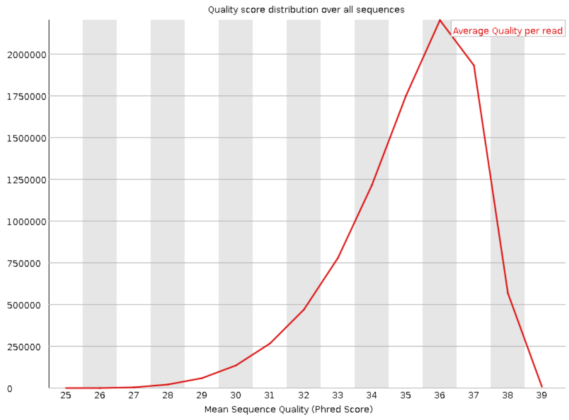

Duplication levels

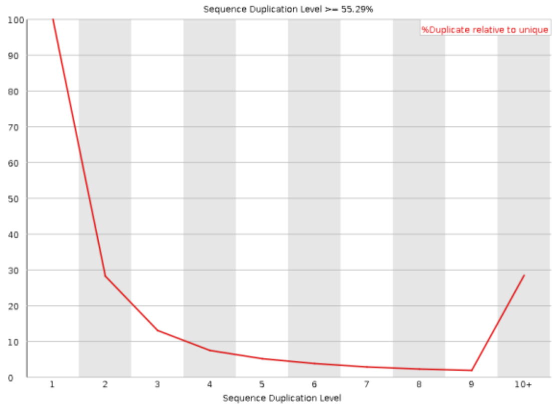

Sequence length distribution

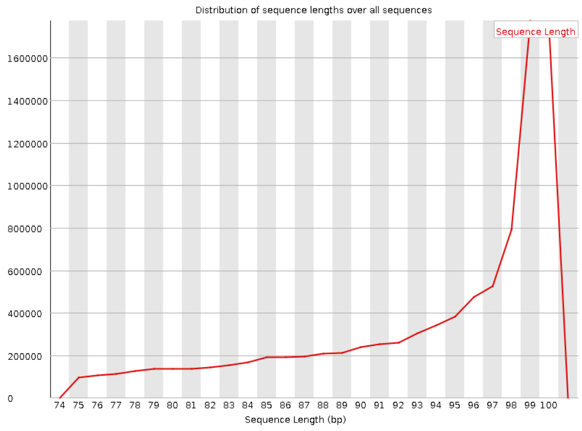

Stem Secondary xylem 12-year-old trees. Repetition 2

Per base quality

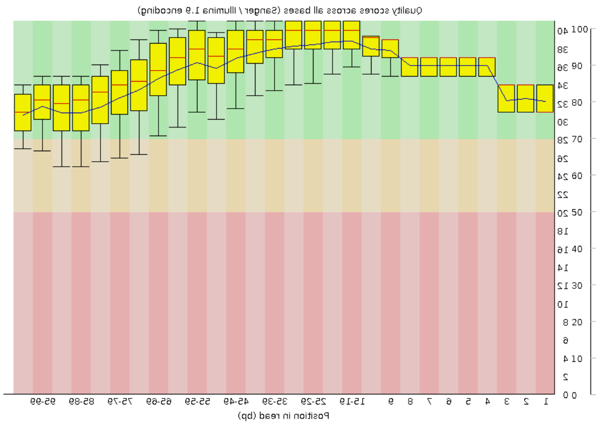

Per base sequence content

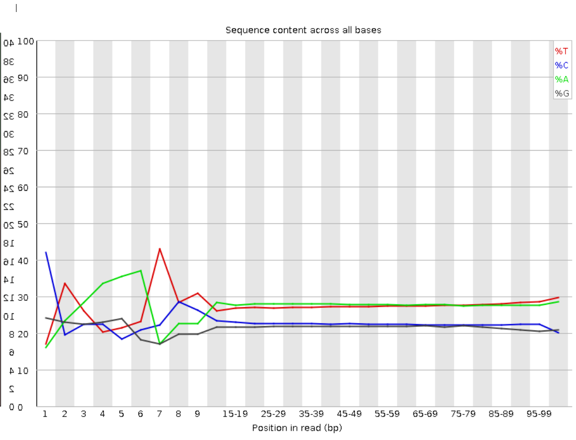

Per sequence GC content

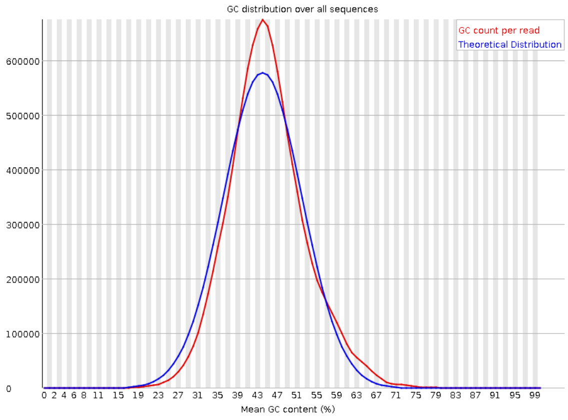

Per sequence quality

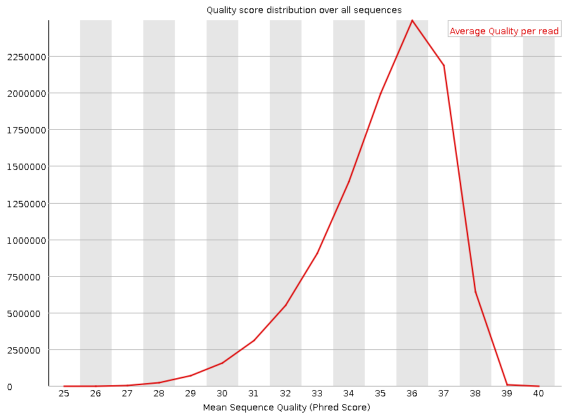

Duplication levels

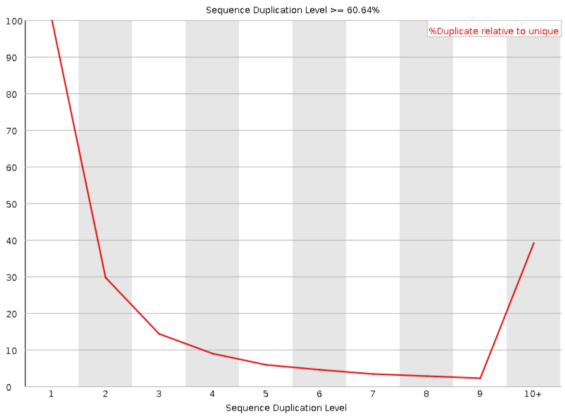

Sequence length distribution

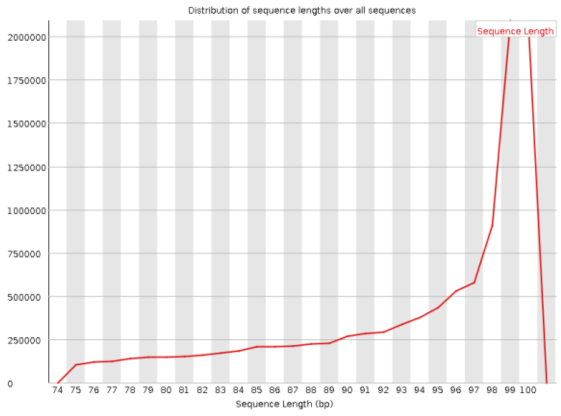

Stem Secondary xylem 60-year-old trees. Repetition 1

Per base quality

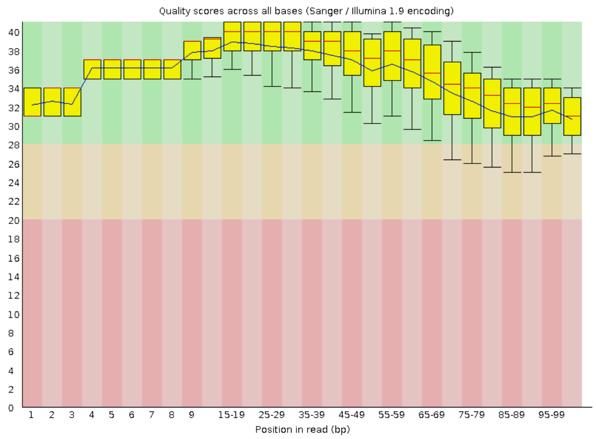

Per base sequence content

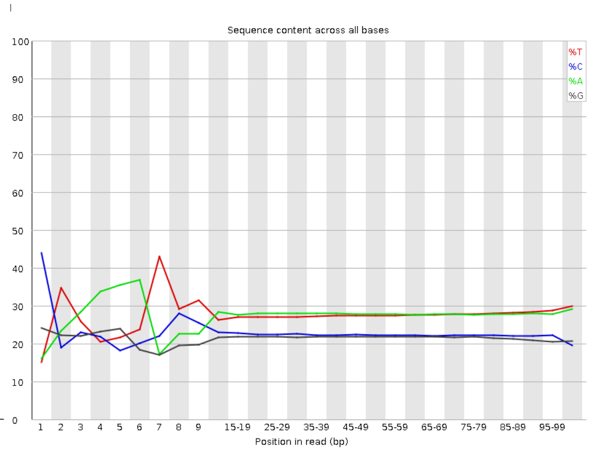

Per sequence GC content

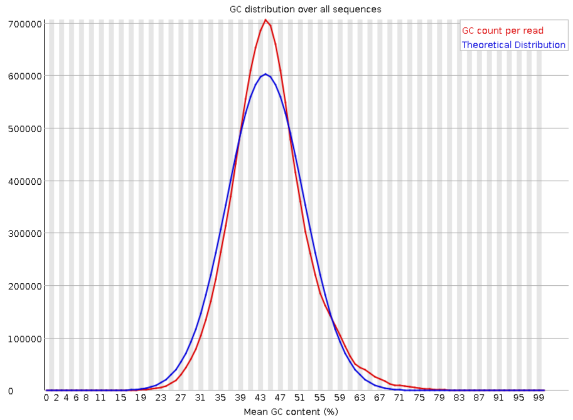

Per sequence quality

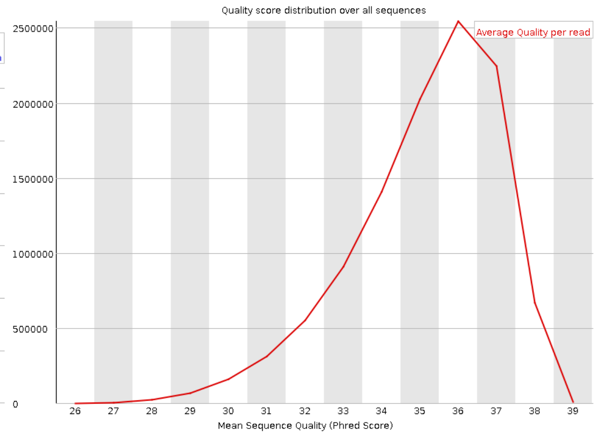

Duplication levels

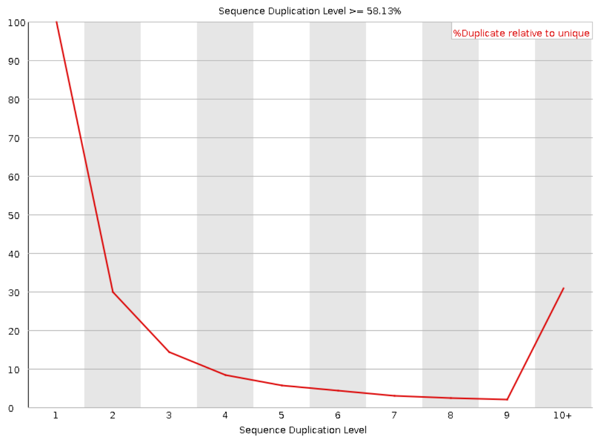

Sequence length distribution

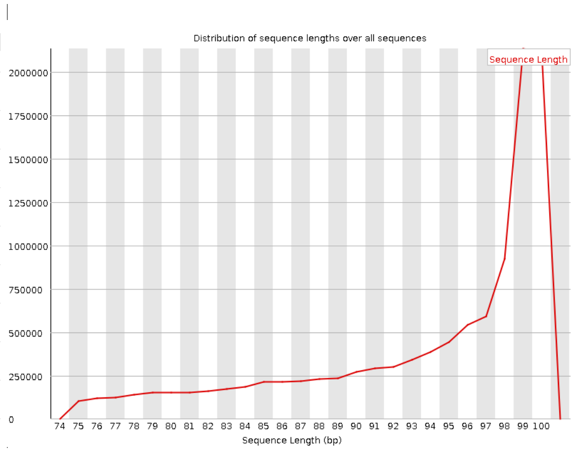

Stem Secondary xylem 60-year-old trees. Repetition 2

Per base quality

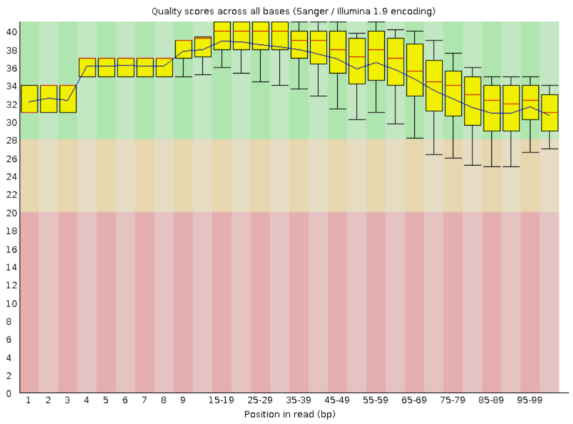

Per base sequence content

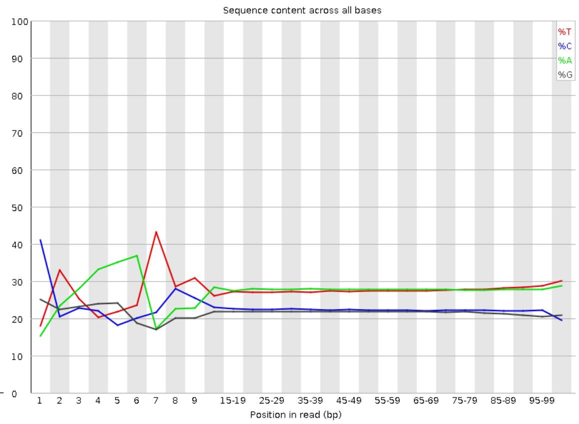

Per sequence GC content

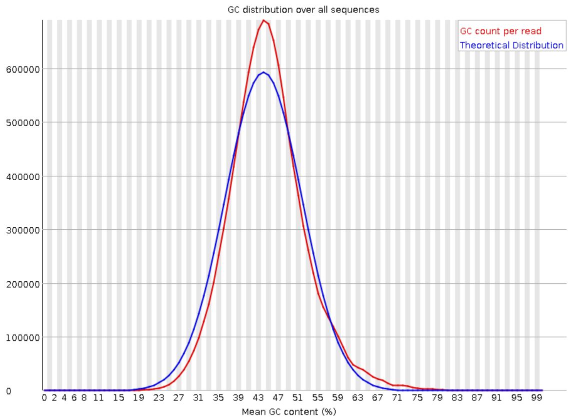

Per sequence quality

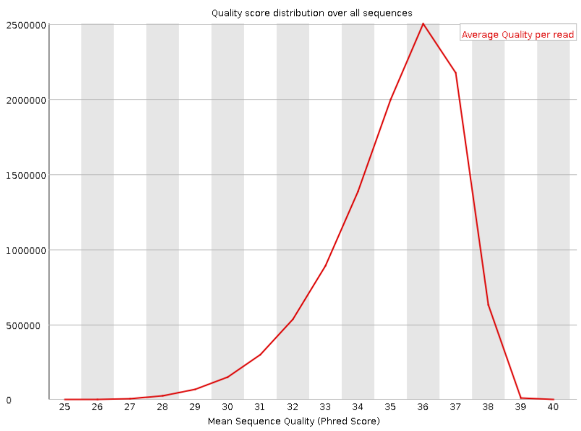

Duplication levels

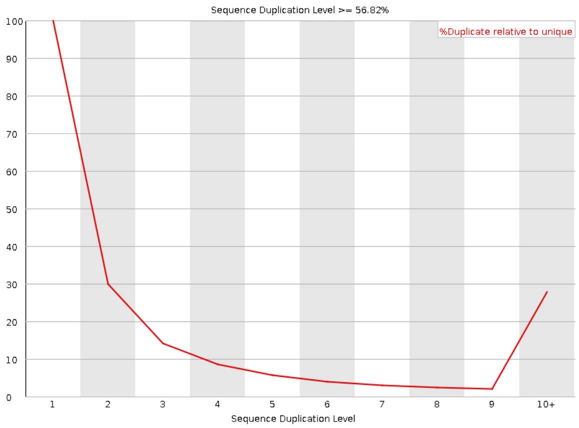

Sequence length distribution

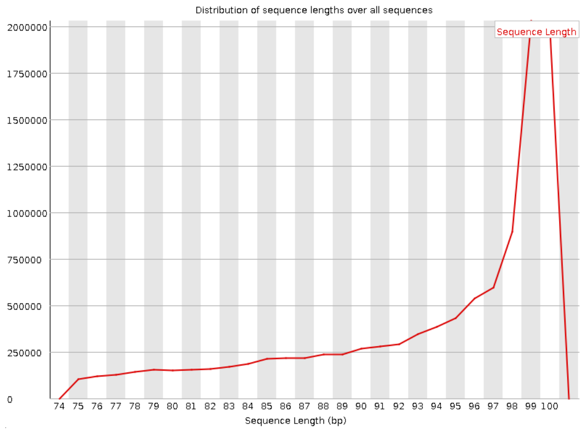

Flower

Per base quality

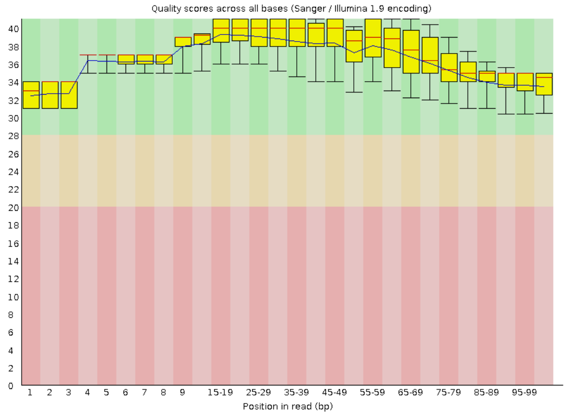

Per base sequence content

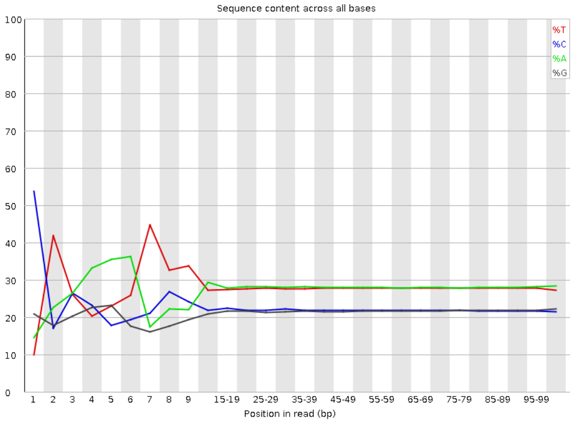

Per sequence GC content

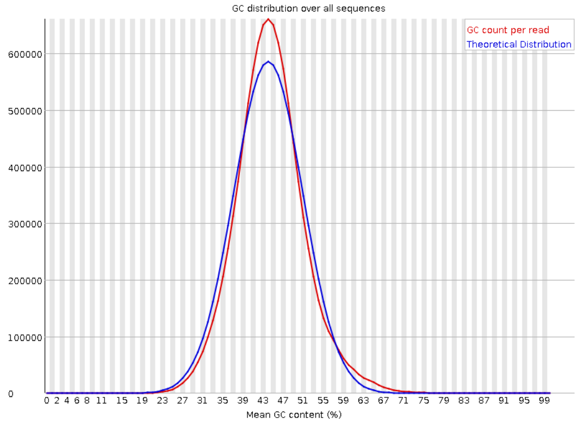

Per sequence quality

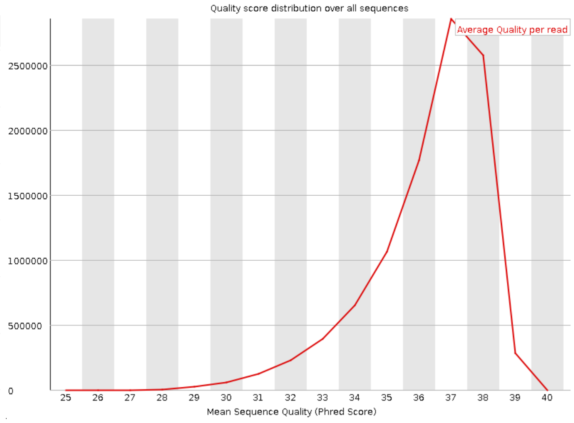

Duplication levels

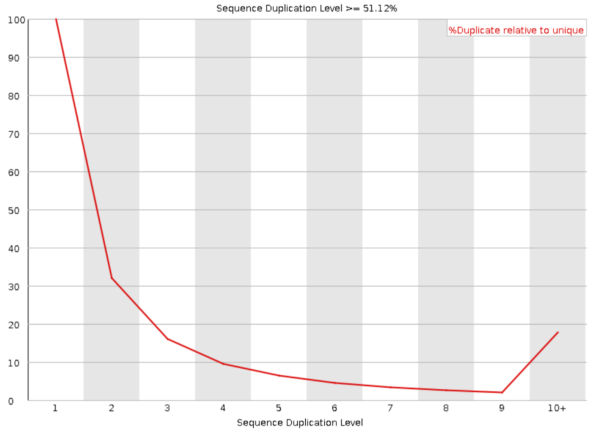

Sequence length distribution

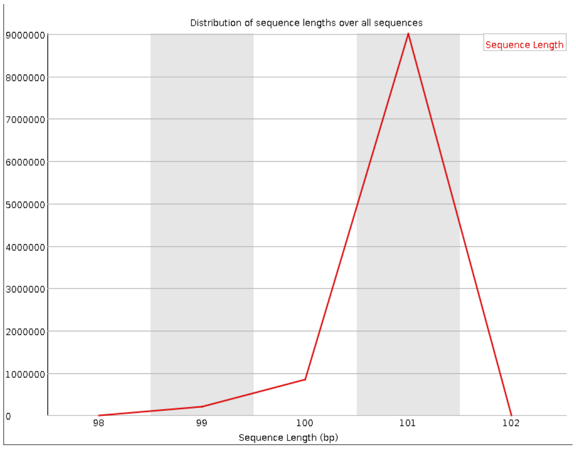

Leaf

Per base quality

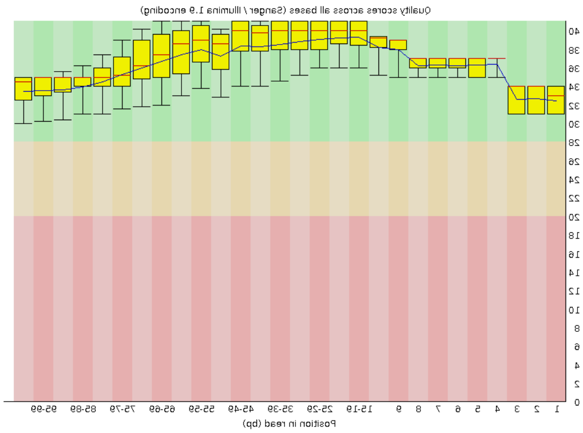

Per base sequence content

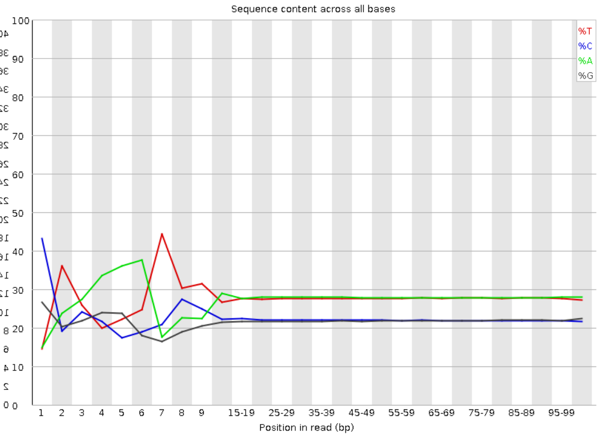

Per sequence GC content

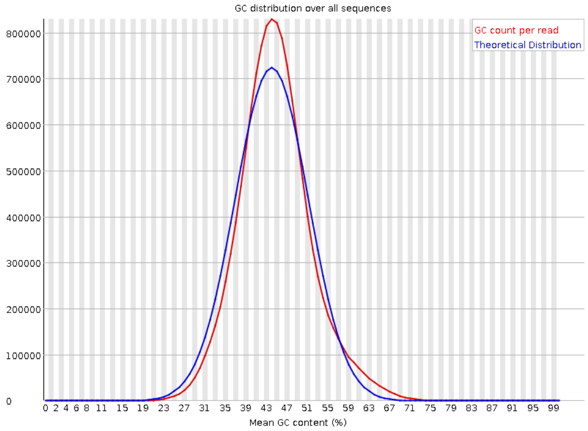

Per sequence quality

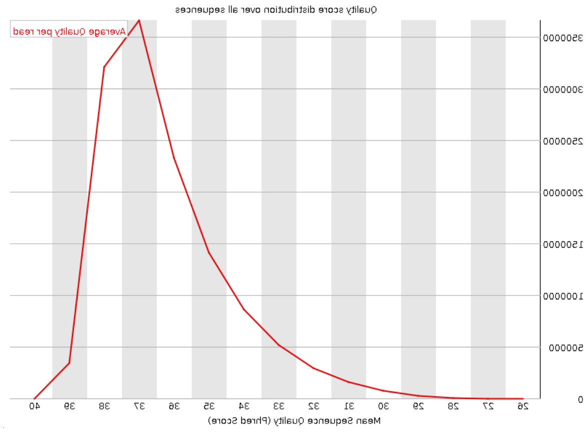

Duplication levels

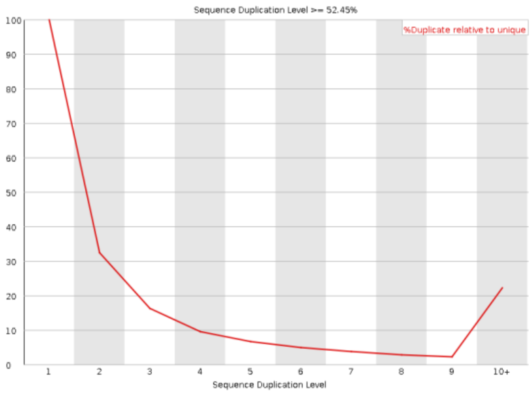

Sequence length distribution

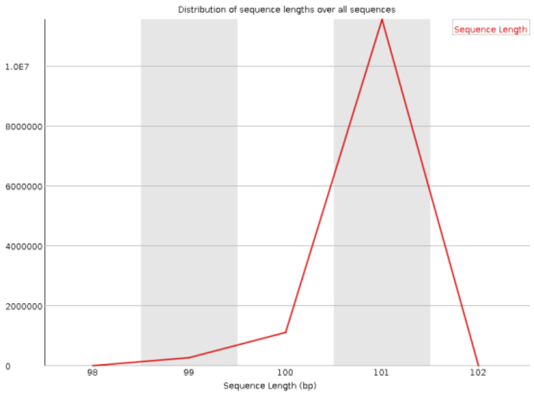

Root

Per base quality

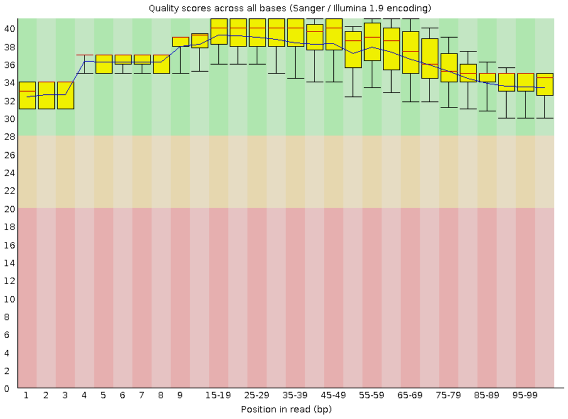

Per base sequence content

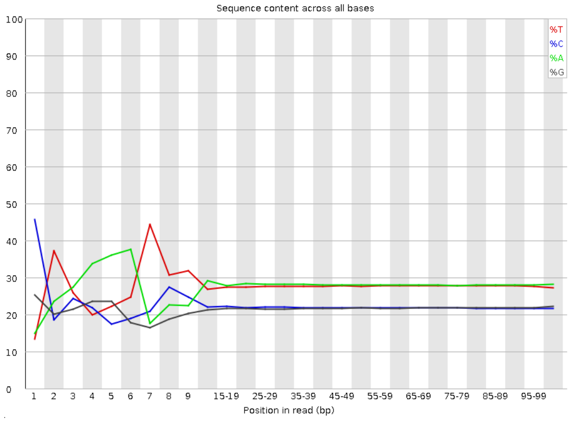

Per sequence GC content

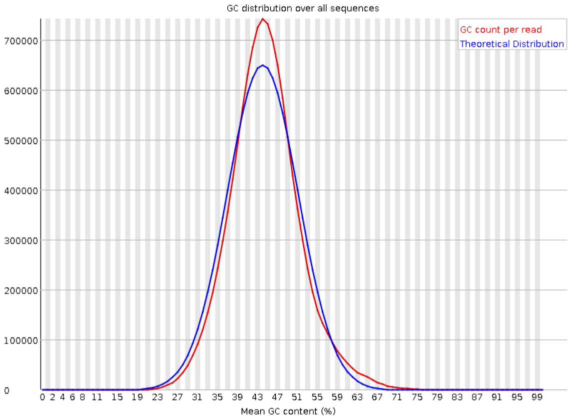

Per sequence quality

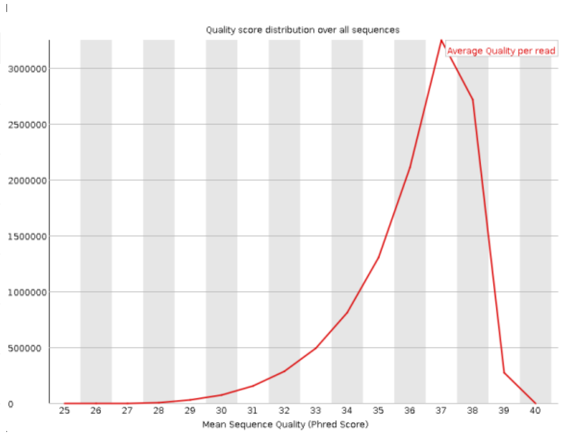

Duplication levels

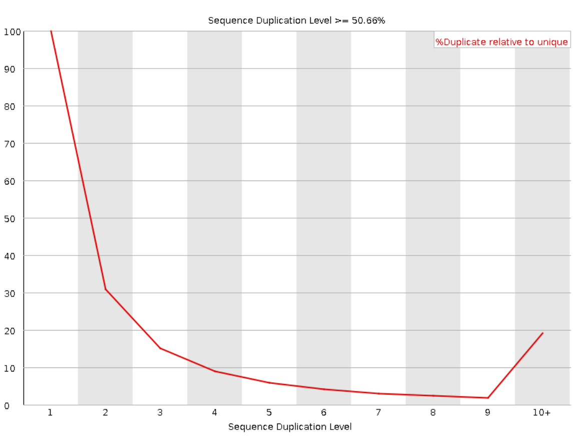

Sequence length distribution

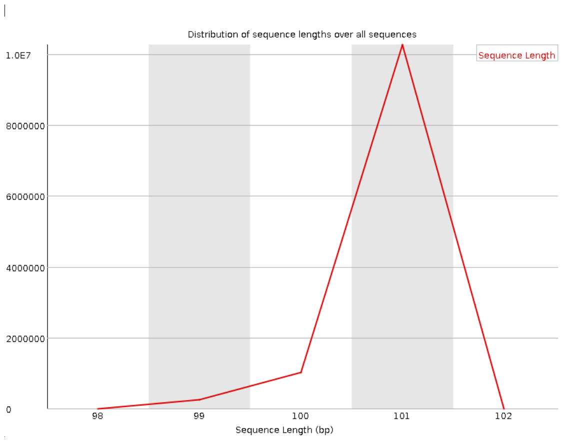

Seedling

Per base quality

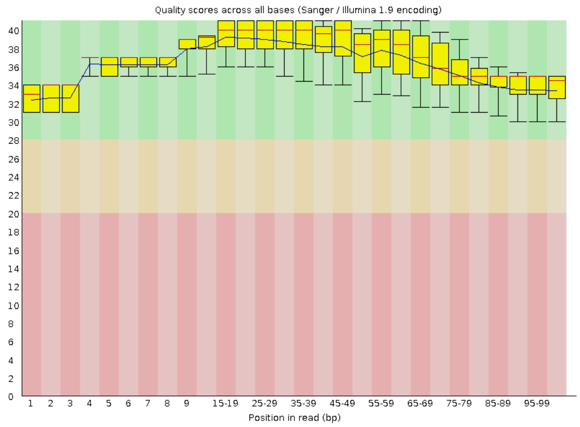

Per base sequence content

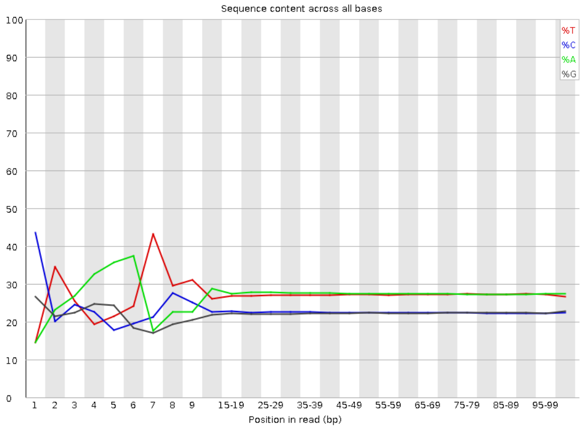

Per sequence GC content

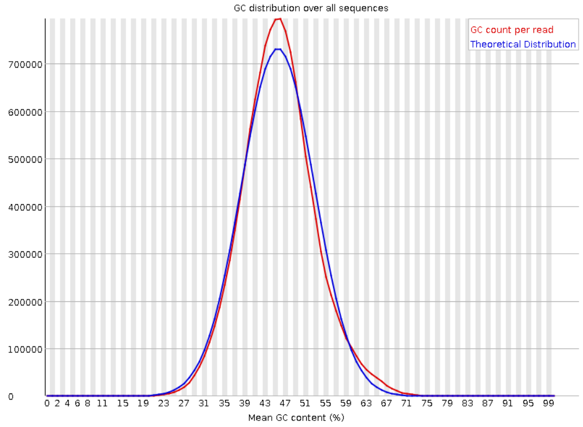

Per sequence quality

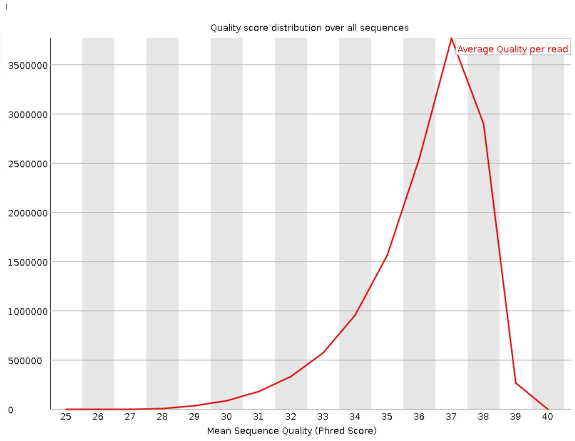

Duplication levels

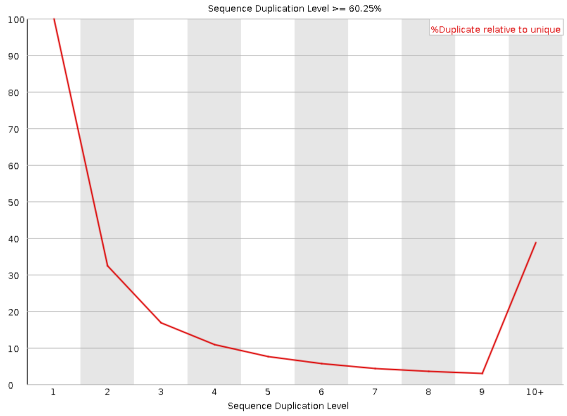

Sequence length distribution

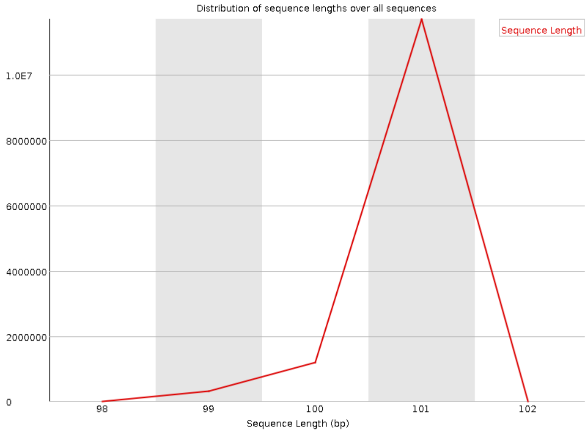

Supplement: Additional file 2: — FASTQC reports of each sample from the RNAseq of Tectona grandis. (PDF 2000 kb) [file 12870_2015_599_MOESM2_ESM.pdf]
