# Supplementary material for: Large-scale transcriptional profiling of lignified tissues in Tectona grandis
Source: BMC Plant Biol. 2015 Sep 15;15:221. doi: 10.1186/s12870-015-0599-x (PMC4570228; doi:10.1186/s12870-015-0599-x)
Supplement: Additional file 3: — Raw data, cleaning data and assembly. (PDF 93 kb) [file 12870_2015_599_MOESM3_ESM.pdf]

Additional File 3. Raw data, cleaning data and assembly.

Tissues	Raw data	Clean data*	% Errased reads	Total trinity transcripts	Total trinity components	Contig N50
Stem secondary xylem 12yoR1	14168695	12640036	10.79	112850	48633	2291
Stem secondary xylem 12yoR2	16166720	14439726	10.68			
Stem secondary xylem 60yoR1	16412620	14618307	10.93			
Stem secondary xylem 60yoR2	16207144	14402146	11.14			
Branch secondary xylem 12yoR1	14185715	12838285	9.5	139535	59771	2365
Branch secondary xylem 12yoR2	14133086	12698822	10.15			
Branch secondary xylem 60yoR1	18783055	18081842	3.73			
Branch secondary xylem 60yoR2	15990913	15384693	3.79			
Flower	13725131	12348918	10.03	129126	65592	2178
Leaf	17947895	16010790	10.79			
Root	16248866	14411320	11.3			
Seedling	18871794	16653783	11.75			
Unpaired				80749	53522	1725
TOTAL	192841634	174528668		462260	227518	
Media			9.55			2140

*Includes unpaired data   R1= replicate 1   R2= replicate 2
