# Supplementary material for: Large-scale transcriptional profiling of lignified tissues in Tectona grandis
Source: BMC Plant Biol. 2015 Sep 15;15:221. doi: 10.1186/s12870-015-0599-x (PMC4570228; doi:10.1186/s12870-015-0599-x)

**Additional File 4. Differential expression of log2 ratio (fold change) versus mean between different conditions with DESeq program.** a) Dispersion plot for branch secondary xylem transcripts. b) Significantly differentially expressed transcripts scatterplot for branch secondary xylem transcripts. c) Dispersion plot for stem secondary xylem transcripts. d) Significantly differentially expressed transcripts scatterplot for stem secondary xylem transcripts. e) Significantly differentially expressed transcripts scatterplot for branch secondary xylem against flower, seedling, leaf and root. f) Significantly differentially expressed transcripts scatterplot for stem secondary xylem against flower, seedling, leaf and root. Fitted curve of the spots is in red. Red dots indicate transcripts differentially expressed at 10% false discovery rate and black spots transcripts are expressed in common [63].

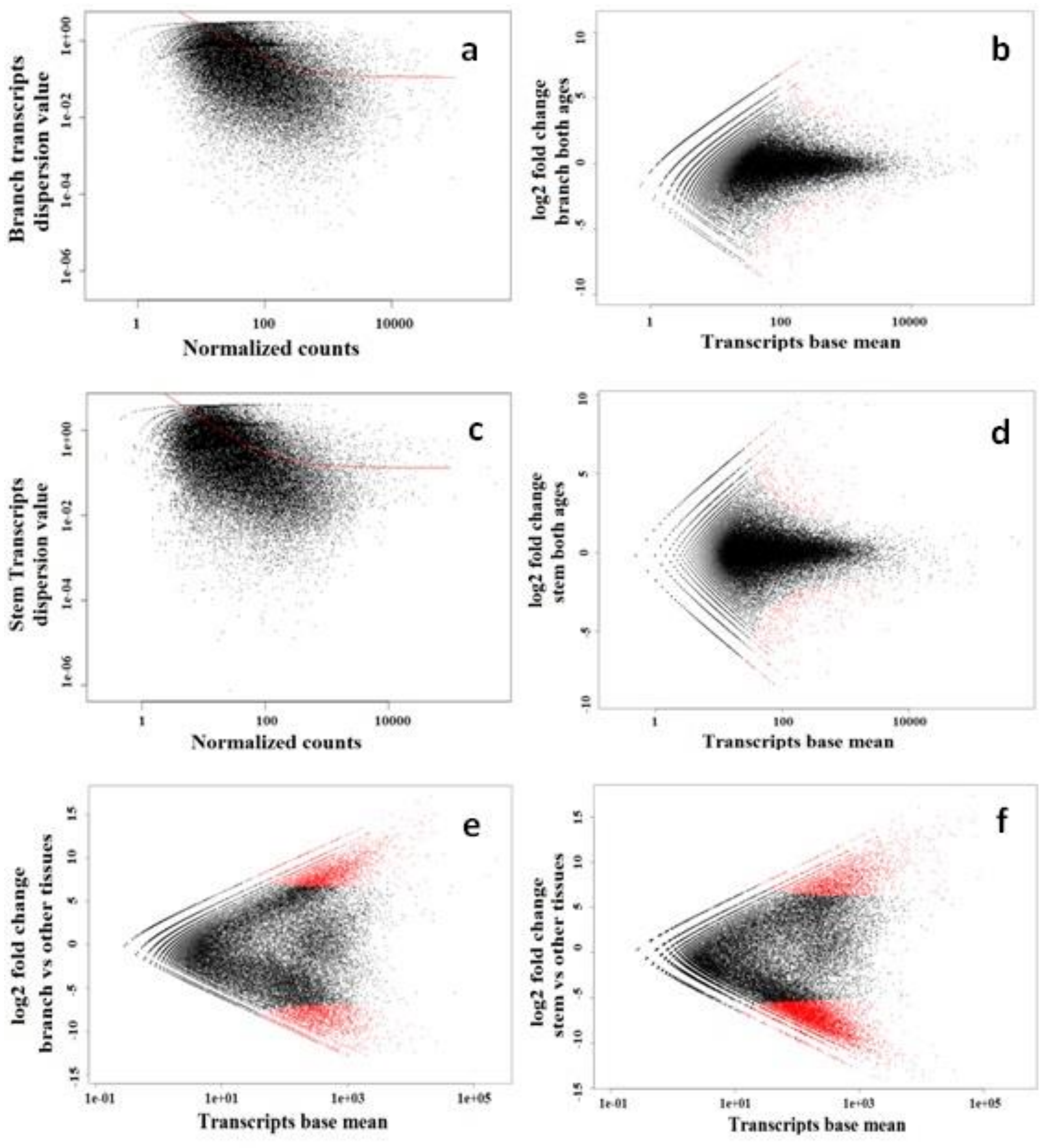

Supplement: Additional file 4: — Differential expression of log2 ratio (fold change) versus mean between different conditions with DESeq program. a) Dispersion plot for branch secondary xylem transcripts. b) Significantly differentially expressed transcripts scatterplot for branch secondary xylem transcripts. c) Dispersion plot for stem secondary xylem transcripts. d) Significantly differentially expressed transcripts scatterplot for stem secondary xylem transcripts. e) Significantly differentially expressed transcripts scatterplot for branch secondary xylem against flower, seedling, leaf and root. f) Significantly differentially expressed transcripts scatterplot for stem secondary xylem against flower, seedling, leaf and root. Fitted curve of the spots is in red. Red dots indicate transcripts differentially expressed at 10 % false discovery rate and black spots transcripts are expressed in common [61]. (PDF 132 kb) [file 12870_2015_599_MOESM4_ESM.pdf]
