# Supplementary material for: Large-scale transcriptional profiling of lignified tissues in Tectona grandis
Source: BMC Plant Biol. 2015 Sep 15;15:221. doi: 10.1186/s12870-015-0599-x (PMC4570228; doi:10.1186/s12870-015-0599-x)

Additional File 5. Significantly differentially expressed transcripts plot for stem-branch genes. Red plots indicate transcripts differentially expressed and black spots transcripts expressed in common.

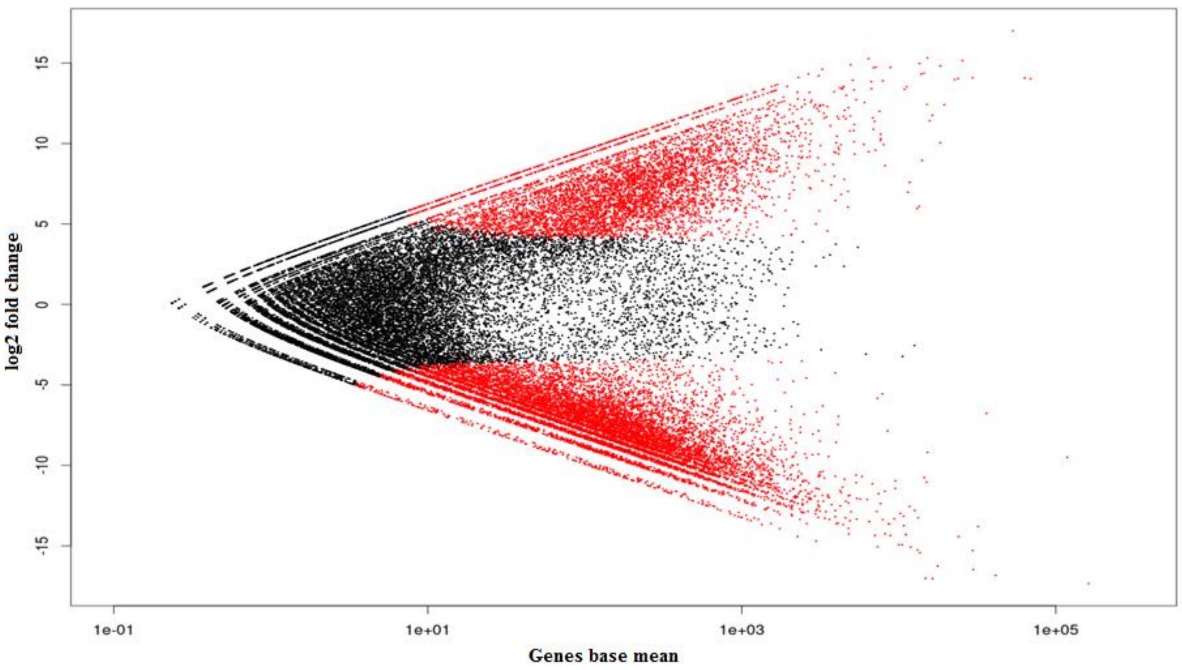

Supplement: Additional file 5: — Significantly differentially expressed transcripts plot for stem-branch genes. Red plots indicate transcripts differentially expressed and black spots transcripts expressed in common. (PDF 144 kb) [file 12870_2015_599_MOESM5_ESM.pdf]
