# Supplementary material for: Large-scale transcriptional profiling of lignified tissues in Tectona grandis
Source: BMC Plant Biol. 2015 Sep 15;15:221. doi: 10.1186/s12870-015-0599-x (PMC4570228; doi:10.1186/s12870-015-0599-x)

Additional File 8. Gene ontology (GO) assignment for the unigenes differentially expressed of *T. grandis* branch secondary xylem. GO assignments (multilevel pie chart with term filter value 5) as predicted for (a) biological process, (b) molecular function and (c) cellular components. The number of unigenes assigned to each GO term is shown behind semicolon.

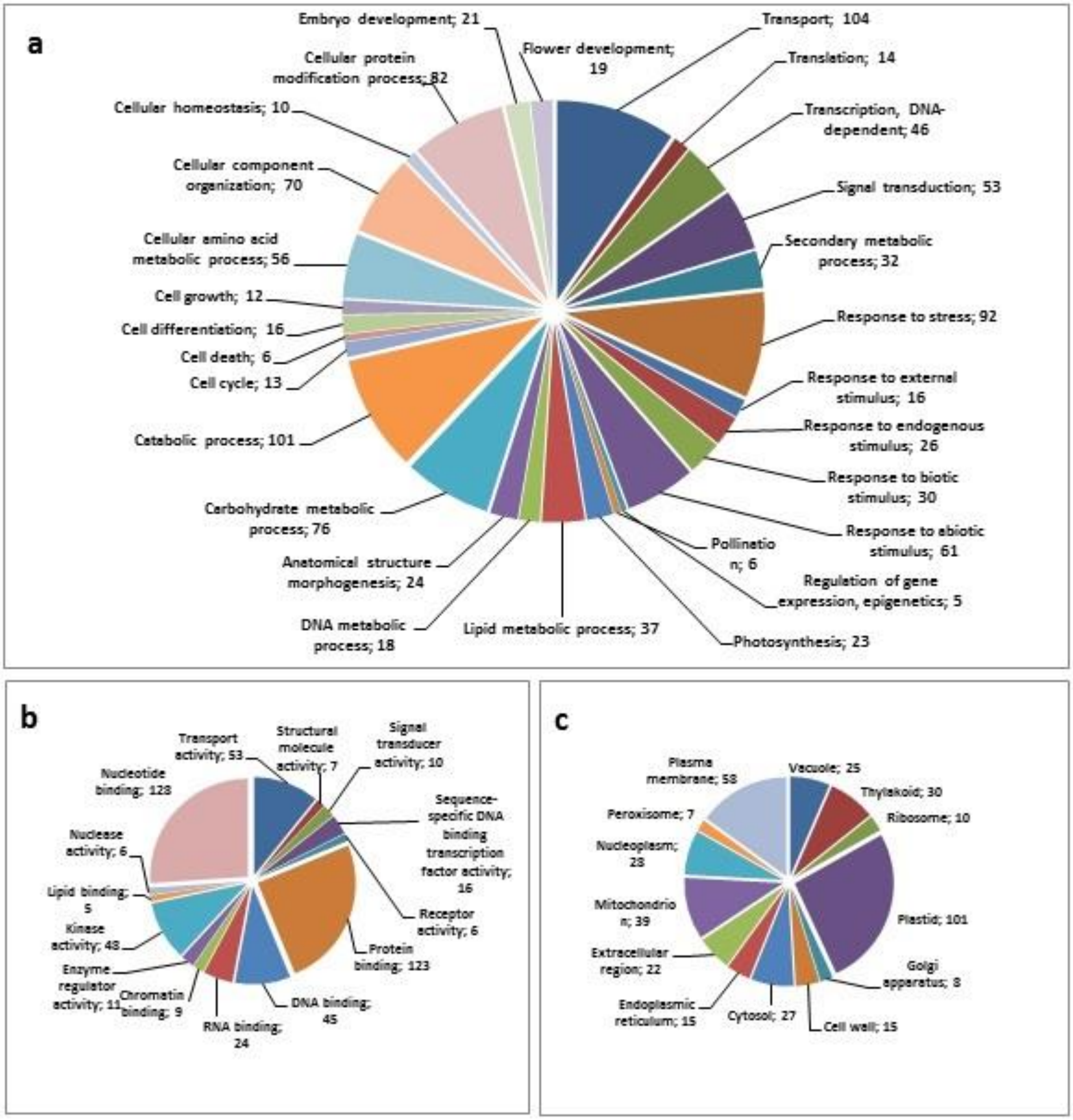

Supplement: Additional file 8: — Gene ontology (GO) assignment for the unigenes differentially expressed of T. grandis branch secondary xylem. GO assignments (multilevel pie chart with term filter value 5) as predicted for (a) biological process, (b) molecular function and (c) cellular components. The number of unigenes assigned to each GO term is shown behind semicolon. (PDF 143 kb) [file 12870_2015_599_MOESM8_ESM.pdf]
