# Supplementary material for: Large-scale transcriptional profiling of lignified tissues in Tectona grandis
Source: BMC Plant Biol. 2015 Sep 15;15:221. doi: 10.1186/s12870-015-0599-x (PMC4570228; doi:10.1186/s12870-015-0599-x)
Supplement: Additional file 9: — GO frequencies for differentially expressed (DE) transcripts. Stem and Branch secondary xylem were the tissues presented in the table. DE transcripts were obtained when comparing 12- and 60-year-old teak trees in both tissues. The first column for GO frequencies is organized from lowest to highest. (PDF 119 kb) [file 12870_2015_599_MOESM9_ESM.pdf]

Additional File 9. GO frequencies for differentially expressed (DE) transcripts. Stem and Branch secondary xylem were the tissues presented in the table. DE transcripts were obtained when comparing 12- and 60-year-old teak trees in both tissues. The first column for GO frequencies is organized from lowest to highest.

**GO Category: BIOLOGICAL PROCESS**	**GO Frequencies**	
	**Stem Secondary xylem**	**Branch Secondary xylem**
Catabolic Process	134	101
Cellular protein modification process	118	10
Response to stress	117	92
Cellular amino acid metabolic process	104	56
Carbohydrate metabolic process	99	76
Response to abiotic stimulus	93	61
Signal transduction	79	53
Lipid metabolic process	73	37
Transcription, DNA-dependent	63	46
Reproduction	61	0
Secondary metabolic process	52	32
Generation of precursor metabolites and energy	45	30
Anatomical structure morphogenesis	42	24
Embryo development	34	21
Response to endogenous stimulus	33	26
Ion transport	33	0
Response to biotic stimulus	32	30
Protein transport	32	0
DNA metabolic process	31	18
Cell cycle	28	13
Cell differentiation	26	16
Response to external stimulus	23	16
Translation	21	14
Cytoskeleton organization	17	0
Cell growth	14	12
Cellular homeostasis	13	10
Cell death	9	6
Mitochondrion organization	7	0
Transport	0	104
Regulation of gene expression, epigenetics	0	5
Pollination	0	6
Photosynthesis	0	23
Cellular component organization	0	70
Flower development	0	19
**TOTAL**	**1433**	**1027**

GO Category: MOLECULAR FUNCTION	GO Frequencies	
	**Stem Secondary xylem**	**Branch Secondary xylem**
Nucleotide binding	194	128
Protein binding	159	123
Protein kinase activity	60	0
DNA binding	45	45
RNA binding	35	24
Peptidase activity	32	0
Sequence-specific DNA binding transcription factor activity	25	16
Nuclease activity	18	6
Calcium ion binding	18	0
Electron carrier activity	15	0
Enzyme regulator activity	14	11
Structural molecule activity	12	7
Ion channel activity	11	0
Phosphoprotein phosphatase activity	9	0
Carbohydrate binding	9	0
Signal transducer activity	8	10
Lipid binding	5	5
Transport activity	0	53
Receptor activity	0	6
Chromatin binding	0	9
Kinase activity	0	48
**TOTAL**	**669**	**491**

GO Category: CELLULAR COMPONENT	GO Frequencies	
	**Stem Secondary xylem**	**Branch Secondary xylem**
Plastid	138	101
Protein complex	94	0
Plasma membrane	87	58
Cytosol	61	27
Mitochondrion	55	39
Vacuole	37	25
Nucleoplasm	34	28
Endoplasmic reticulum	33	15
Thylakoid	30	30
Extracellular region	30	22
Ribosome	16	10
Golgi apparatus	15	8
Cell wall	11	15
Nucleolus	9	0
Endosome	7	0
Chromosome	6	0
Peroxisome	5	7
Cytoskeleton	5	0
**TOTAL**	**673**	**385**
