# Supplementary material for: Large-scale transcriptional profiling of lignified tissues in Tectona grandis
Source: BMC Plant Biol. 2015 Sep 15;15:221. doi: 10.1186/s12870-015-0599-x (PMC4570228; doi:10.1186/s12870-015-0599-x)
Supplement: Additional file 10: — 43 genes highly differentially expressed between stem secondary xylem from 12- and 60-year-old trees. (PDF 106 kb) [file 12870_2015_599_MOESM10_ESM.pdf]

Additional File 10. 43 genes highly differentially expressed between stem secondary xylem from 12- and 60-year-old trees.

Gene	baseMean Stem secondary xylem 12yo	baseMean Stem secondary xylem 60yo	p value
transmembrane bax inhibitor motif-containing	6097.3	868	0
NTGP3 putative rac protein	2858.4	482.2	0.00186308
kda class i heat shock	5769.4	898.1	0.00065701
splicing factor U2af small subunit A-like	2229.9	266.8	0.0001194
heat shock 70 kda	1147.2	244.6	0.02134347
voltage-gated potassium channel subunit beta-like	1591.5	122.3	3.39E-06
atp binding cassette subfamily b4 isoform 2	2252.4	148.1	3.63E-07
galactinol--sucrose galactosyltransferase 2-like isoform X1	1299	15568.5	9.87E-07
carboxylesterase 8-like	6.8	4352.9	3.17E-25
chaperone	1790.9	289.7	0.00191109
chlorophyll a b binding	4640.4	676.8	0.00041139
protein mizu-kussei 1-like	1629.2	318.2	0.00859593
oligopeptide transporter 1	3507	658.8	0.00399448
glucose-6-phosphate phosphate translocator chloroplastic-like	163	1158.9	0.00115311
oxygen-evolving enhancer protein ,. chloroplastic-like	1696.1	393.8	0.02730737
desumoylating isopeptidase 1-like	4061.1	782.2	0.00456294
nuclear pore complex protein nup98-nup96-like isoform x2	1849.9	70.4	2.04E-09
kda class i heat shock protein	146647	38425.2	0.03359285
heat shock protein 83-like	3620.1	376.1	2.02E-05
ribulose bisphosphate carboxylase oxygenase activase chloroplastic-like	1890.4	186.3	2.60E-05
large proline-rich protein bag6-like isoform x1	141.1	1527.4	2.13E-05
f-box protein skip27-like	1201.4	269.8	0.02854693
kda heat shock peroxisomal-like	22363	3960.7	0.00159461
heat shock protein 83-like	90973.5	22710	0.02386294
heat shock protein 83-like	11170	1237.1	2.12E-05
pre-mrna-splicing factor cef1-like	1674.8	359.6	0.01647392
Inositol-3-phosphate synthase	3625	458.8	0.00012983
nuclear pore complex protein nup98-nup96-like isoformx1	1682.8	72.6	9.07E-09
bi1-like	13792.6	1735.6	7.21E-05
peptidyl-prolyl cis-trans isomerase FKBP65-like	1870	259.3	0.00053044
kda class i heat shock	31868.3	8141.1	0.02882191
nadh dehydrogenase	1759.6	427.8	0.03588352
nucleoporin	8824.3	292.8	2.71E-11
diacylglycerol kinase 1-like isoform X1	1724	252.6	0.00089485
protein vip1-like isoform X2	1444.5	234	0.00244917
protein dj-1 homolog b-like	2427.3	564.1	0.02270032
glucosidase 2 subunit beta-like	1862.5	148.9	3.66E-06
kda class i heat shock	1588.1	87.8	1.41E-07
trans-alpha-bergamotene synthase	197.5	1281.1	0.00191109
glutathione s-	3156.2	423	0.00024414
60s ribosomal protein l10-like	4243	1091.4	0.03743841
luminal binding protein	4809.8	1082.3	0.01417015
