# Supplementary material for: Large-scale transcriptional profiling of lignified tissues in Tectona grandis
Source: BMC Plant Biol. 2015 Sep 15;15:221. doi: 10.1186/s12870-015-0599-x (PMC4570228; doi:10.1186/s12870-015-0599-x)

Additional File 11. Other relevant differentially expressed genes from secondary xylem. We chose other genes with the highest expression between young (12-years-old) and mature (60-years-old) trees, and performed a transformation of root square in order to visualize their values.

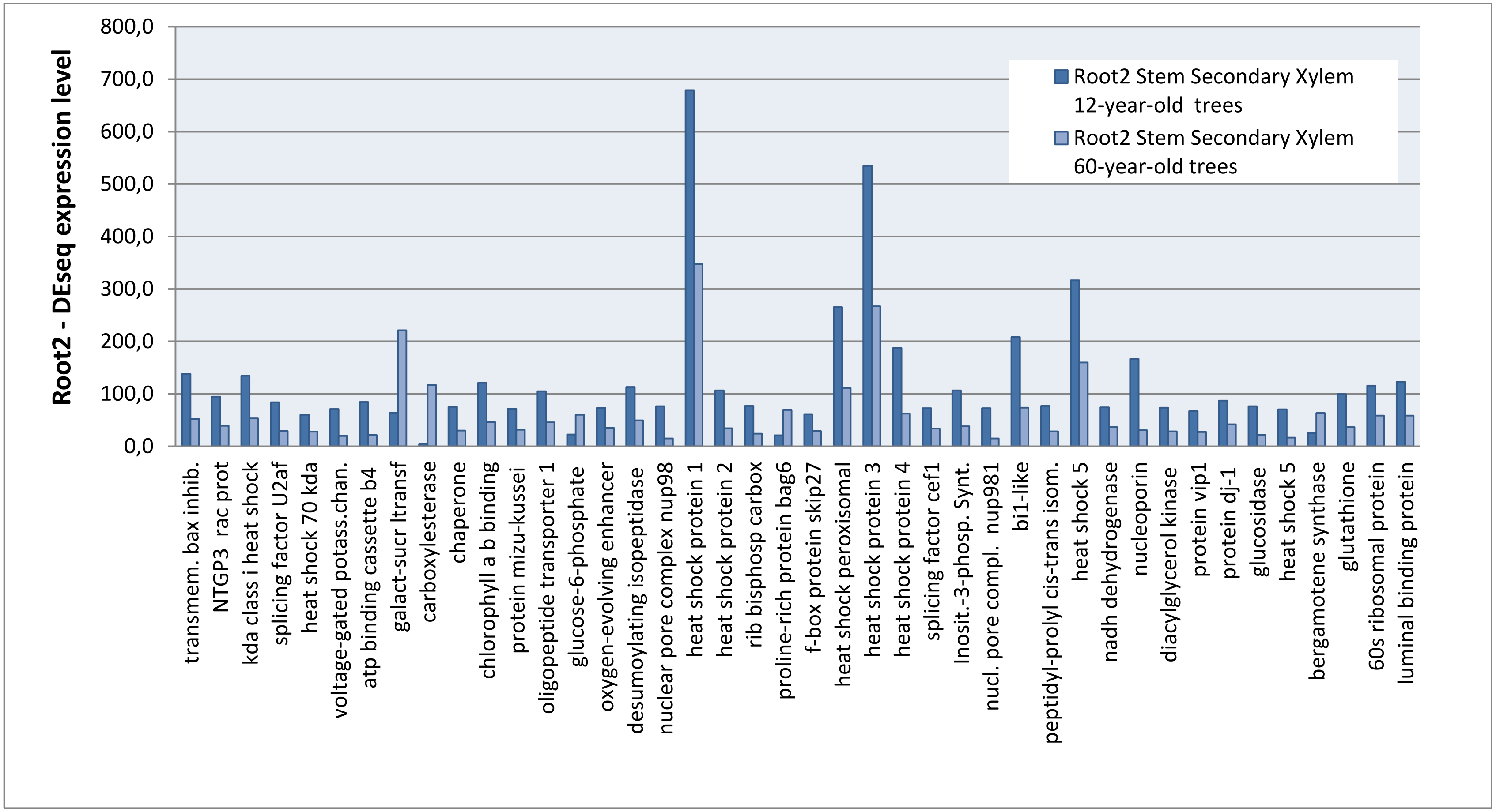

Supplement: Additional file 11: — Other relevant differentially expressed genes from secondary xylem. We chose other genes with the highest expression between young (12-years-old) and mature (60-years-old) trees, and performed a transformation of root square in order to visualize their values. (PDF 178 kb) [file 12870_2015_599_MOESM11_ESM.pdf]
