# Supplementary material for: Large-scale transcriptional profiling of lignified tissues in Tectona grandis
Source: BMC Plant Biol. 2015 Sep 15;15:221. doi: 10.1186/s12870-015-0599-x (PMC4570228; doi:10.1186/s12870-015-0599-x)
Supplement: Additional file 12: — Branch secondary xylem pathways found by Kegg. (PDF 116 kb) [file 12870_2015_599_MOESM12_ESM.pdf]

Additional File 12. Branch secondary xylem pathways found by Kegg.

	Pathways	Number of Sequences	Number of enzymes
1.	Starch and sucrose metabolism	16	10
2.	Amino sugar and nucleotide sugar metabolism	6	3
3.	Purine metabolism	6	2
4.	Methane metabolism	4	3
5.	Porphyrin and chlorophyll metabolism	4	2
6.	Thiamine metabolism	4	2
7.	Glyoxylate and dicarboxylate metabolism	4	2
8.	Galactose metabolism	4	4
9.	Aminobenzoate degradation	3	2
10.	Glycolysis / Gluconeogenesis	3	2
11.	Glutathione metabolism	3	2
12.	Oxidative phosphorylation	3	3
13.	Terpenoid backbone biosynthesis	3	2
14.	T cell receptor signaling pathway	3	1
15.	Pentose phosphate pathway	3	2
16.	Phosphatidylinositol signaling system	3	2
17.	Fructose and mannose metabolism	3	3
18.	Glycerolipid metabolism	3	2
19.	Aminoacyl-tRNA biosynthesis	2	2
20.	Pentose and glucuronate interconversions	2	2
21.	Carbon fixation in photosynthetic organisms	2	1
22.	Riboflavin metabolism	2	1
23.	Carotenoid biosynthesis	2	1
24.	Pantothenate and CoA biosynthesis	2	1
25.	Glycosphingolipid biosynthesis - globo series	2	1
26.	Sphingolipid metabolism	2	1
27.	Sulfur metabolism	2	1
28.	Biosynthesis of terpenoids and steroids	2	1
29.	Inositol phosphate metabolism	2	1
30.	Phenylalanine, tyrosine and tryptophan biosynthesis	2	2
31.	Carbon fixation pathways in prokaryotes	2	2
32.	Biotin metabolism	1	1
33.	Toluene degradation	1	1
34.	Pyruvate metabolism	1	1
35.	Phenylpropanoid biosynthesis	1	1
36.	Butanoate metabolism	1	1
37.	Arginine and proline metabolism	1	2
38.	Phenylalanine metabolism	1	1
39.	Fatty acid degradation	1	1
40.	Glycine, serine and threonine metabolism	1	1
41.	Caprolactam degradation	1	1
42.	Propanoate metabolism	1	1
43.	Fatty acid elongation	1	1
44.	Fatty acid biosynthesis	1	1
45.	Tryptophan metabolism	1	1
46.	N-Glycan biosynthesis	1	1
47.	Geraniol degradation	1	1
48.	Valine, leucine and isoleucine degradation	1	1
49.	Nicotinate and nicotinamide metabolism	1	1
50.	Primary bile acid biosynthesis	1	1
51.	Lysine degradation	1	1
52.	Ascorbate and aldarate metabolism	1	1
53.	Glycosaminoglycan biosynthesis - heparan sulfate / heparin	1	1
54.	Glycerophospholipid metabolism	1	1
55.	alpha-Linolenic acid metabolism	1	1
56.	Linoleic acid metabolism	1	1
57.	Cysteine and methionine metabolism	1	1
