# Supplementary material for: Large-scale transcriptional profiling of lignified tissues in Tectona grandis
Source: BMC Plant Biol. 2015 Sep 15;15:221. doi: 10.1186/s12870-015-0599-x (PMC4570228; doi:10.1186/s12870-015-0599-x)
Supplement: Additional file 13: — Stem secondary xylem pathways found by Kegg. (PDF 129 kb) [file 12870_2015_599_MOESM13_ESM.pdf]

Additional File 13. Stem secondary xylem pathways found by Kegg.

	Pathways	Number of Sequences	Number of enzymes
1	Starch and sucrose metabolism	17	10
2	Glycerolipid metabolism	16	4
3	Purine metabolism	15	8
4	Glycerophospholipid metabolism	15	5
5	Phosphatidylinositol signaling system	15	4
6	Glycolysis / Gluconeogenesis	11	5
7	Carbon fixation in photosynthetic organisms	10	4
8	Fructose and mannose metabolism	10	3
9	Pyruvate metabolism	9	7
10	Pentose phosphate pathway	9	3
11	Nicotinate and nicotinamide metabolism	9	1
12	Methane metabolism	8	2
13	Selenocompound metabolism	8	4
14	Galactose metabolism	8	7
15	Aminoacyl-tRNA biosynthesis	7	4
16	Cysteine and methionine metabolism	7	6
17	Valine, leucine and isoleucine degradation	6	5
18	Terpenoid backbone biosynthesis	6	3
19	Propanoate metabolism	6	4
20	Pyrimidine metabolism	6	4
21	Carbon fixation pathways in prokaryotes	5	4
22	Inositol phosphate metabolism	5	4
23	Thiamine metabolism	5	2
24	Pentose and glucuronate interconversions	5	2
25	Oxidative phosphorylation	4	3
26	Streptomycin biosynthesis	4	3
27	Glutathione metabolism	4	3
28	Porphyrin and chlorophyll metabolism	4	4
29	Sphingolipid metabolism	4	2
30	Glyoxylate and dicarboxylate metabolism	4	4
31	Drug metabolism - other enzymes	3	2
32	Arginine and proline metabolism	3	3
33	Amino sugar and nucleotide sugar metabolism	3	3
34	Other glycan degradation	3	2
35	Citrate cycle (TCA cycle)	3	3
36	Lysine degradation	3	3
37	Glycine, serine and threonine metabolism	3	2
38	Fatty acid biosynthesis	3	2
39	alpha-Linolenic acid metabolism	2	2
40	Sulfur metabolism	2	1
41	Drug metabolism - cytochrome P450	2	1
42	Metabolism of xenobiotics by cytochrome P450	2	1
43	Aminobenzoate degradation	2	1
44	Tryptophan metabolism	2	2
45	Pantothenate and CoA biosynthesis	2	2
46	Sesquiterpenoid and triterpenoid biosynthesis	2	2
47	Carotenoid biosynthesis	2	1
48	Monoterpenoid biosynthesis	2	2
49	Penicillin and cephalosporin biosynthesis	2	2
50	Glycosphingolipid biosynthesis - ganglio series	2	1
51	Glycosphingolipid biosynthesis - globo series	2	1
52	Fatty acid degradation	2	2
53	T cell receptor signaling pathway	2	2
54	Biosynthesis of terpenoids and steroids	2	1
55	Flavonoid biosynthesis	2	2
56	Phenylpropanoid biosynthesis	2	2
57	Aflatoxin biosynthesis	2	1
58	Tetracycline biosynthesis	2	1
59	Alanine, aspartate and glutamate metabolism	2	2
60	Riboflavin metabolism	2	1
61	Ascorbate and aldarate metabolism	2	2

62	Glycosaminoglycan degradation	2	1
63	Linoleic acid metabolism	1	1
64	Biosynthesis of unsaturated fatty acids	1	2
65	Arachidonic acid metabolism	1	1
66	Taurine and hypotaurine metabolism	1	1
67	Insect hormone biosynthesis	1	1
68	Chloroalkane and chloroalkene degradation	1	1
69	Butirosin and neomycin biosynthesis	1	1
70	Zeatin biosynthesis	1	2
71	Limonene and pinene degradation	1	1
72	beta-Alanine metabolism	1	1
73	Indole alkaloid biosynthesis	1	1
74	D-Glutamine and D-glutamate metabolism	1	1
75	beta-Lactam resistance	1	1
76	Synthesis and degradation of ketone bodies	1	1
77	Novobiocin biosynthesis	1	1
78	Phenylalanine, tyrosine and tryptophan biosynthesis	1	2
79	Cyanoamino acid metabolism	1	1
80	Benzoate degradation	1	1
81	Phenylalanine metabolism	1	1
82	Steroid biosynthesis	1	1
83	Butanoate metabolism	1	1
84	Peptidoglycan biosynthesis	1	1
85	Flavone and flavonol biosynthesis	1	1
86	Histidine metabolism	1	1
87	Glycosaminoglycan biosynthesis - heparan sulfate / heparin	1	1
88	Glycosaminoglycan biosynthesis - chondroitin sulfate / dermatan sulfate	1	1
