# Supplementary material for: Large-scale transcriptional profiling of lignified tissues in Tectona grandis
Source: BMC Plant Biol. 2015 Sep 15;15:221. doi: 10.1186/s12870-015-0599-x (PMC4570228; doi:10.1186/s12870-015-0599-x)
Supplement: Additional file 14: — Relevant enzymes found for differentially expressed genes in stem. In Blue, sequences higher than 3000 bp. (PDF 114 kb) [file 12870_2015_599_MOESM14_ESM.pdf]

Additional File 14. Relevant enzymes found for differentially expressed genes in stem. In Blue, sequences higher than 3000 bp.

Metabolism	Enzyme code	Teak sequence	Size	Seq description	% Similarity
Selenocompound metabolism	ec:2.7.7.4 - adenylyltransferase	comp22952_c0_seq14	2193	eukaryotic peptide chain release factor GTP-binding subunit ERF3A-like	81% [Solanum tuberosum]
		comp22952_c0_seq19	2505	Eukaryotic peptide chain release factor GTP-binding subunit ERF3A-like isoform X1	79% [Glycine max]
	ec:6.1.1.10 - ligase	comp23531_c0_seq7	2959	methionine--tRNA ligase-like	93% [vitis vinifera]
		comp23531_c0_seq26	3183	methionine--tRNA ligase-like	92% [Cucumis sativus]
		comp23531_c0_seq16	3070	methionine--tRNA synthetase-like	93% [vitis vinifera]
		comp23531_c0_seq22	1719	methionine--tRNA synthetase-like	86% vitis vinifera]
		comp20538_c0_seq1	1939	isopenicillin N epimerase-like	90% [Solanum lycopersicum]
	ec:4.4.1.1 - gamma-lyase	comp23104_c0_seq3	1104	heme oxygenase 1, chloroplastic-like	85% Solanum lycopersicum]
Porphyrin and chlorophyll metabolism	ec:1.14.99.3 - oxygenase (biliverdin-producing)	comp23104_c0_seq3	1104	heme oxygenase 1, chloroplastic-like	92% [Citrus sinensis]
		comp15255_c0_seq1	1418	protochlorophyllide reductase, chloroplastic-like	89% [Solanum lycopersicum]
	ec:1.3.1.33 - reductase	comp24236_c0_seq1	1452	chlorophyllide a oxygenase, chloroplastic-like	98% [Citrus sinensis]
	ec:1.14.13.122 - oxygenase	comp24729_c1_seq15	1981	Hypoxanthine-guanine phosphoribosyltransferase isoform 2	80% [Theobroma cacao]
Drug metabolism - other enzymes	ec:2.4.2.8 - phosphoribosyltransferase	comp24729_c1_seq8	1480	hypoxanthine-guanine phosphoribosyltransferase-like	89% [Solanum tuberosum]
		comp19743_c0_seq12	3050	hypothetical protein POPTR_0006s26730g	89% [Populus trichocarpa]
	ec:3.1.1.1 - ali-esterase	comp23081_c0_seq6	3578	lysosomal alpha-mannosidase-like	80% [Vitis vinifera
Other glycan degradation	ec:3.2.1.24 - alpha-D-mannosidase	comp23446_c0_seq76	4591	beta-galactosidase 17-like	82% [Solanum lycopersicum]
	ec:3.2.1.23 - lactase (ambiguous)	comp22867_c0_seq18	1503	beta-galactosidase isoform 1	80% [Vitis vinifera]
		comp5905_c0_seq2	1157	glutathione S-transferase parA-like	90% [Solanum lycopersicum]
Sesquiterpenoid and triterpenoid biosynthesis	ec:2.5.1.18 - transferase	comp17040_c0_seq1	744	squalene synthase 2	90% [Salvia miltiorrhiza]
	ec:2.5.1.21 - synthase	comp24494_c1_seq20	1008	dihydroflavonol-4-reductase-like	87% [Solanum lycopersicum]
	ec:1.1.1.216 - dehydrogenase (NADP+)	comp18980_c0_seq15	1459	phytoene synthase	96% [Osmanthus fragrans]
Carotenoid biosynthesis	ec:2.5.1.32 - synthase	comp18980_c0_seq8	1794	phytoene synthase	92% [Nicotiana tabacum]
		comp24674_c0_seq2	1656	isopentenyltransferase	79% [Solanum lycopersicum]
Zeatin	ec:2.5.1.27 -	comp24941_c0_seq2	2908	Adipocyte plasma membrane-associated	81%

biosynthesis	dimethylallyltransferase			protein-like	[Solanum lycopersicum]
Drug Metabolism- Other enzymes	ec:3.1.1.1 - ali-esterase	comp19743_c0_seq12	3050	hypothetical protein POPTR_0006s26730g	87% [Populus trichocarpa]
Drug Metabolism- Other enzymes	ec:2.4.2.8 - phosphoribosyltransferase	comp24729_c1_seq15	1981	Hypoxanthine-guanine phosphoribosyltransferase isoform 2	80% [Theobroma cacao]
