# Supplementary material for: Large-scale transcriptional profiling of lignified tissues in Tectona grandis
Source: BMC Plant Biol. 2015 Sep 15;15:221. doi: 10.1186/s12870-015-0599-x (PMC4570228; doi:10.1186/s12870-015-0599-x)
Supplement: Additional file 15: — Predicted MYB domain protein sequences from Tectona grandis . Amino acid sequences of the four MYB transcription factors were obtained with ExPASy Translate tool (http://web.expasy.org/translate/). Grey shading indicates identical amino acid residues that agree with the motifs referenced by Bedon et al. (2007). MYB-CC type transfactor domain (TgMYB1) and R2R-MYB DNA-binding domains (MYBR2R3-DBDs) (TgMYB2, TgMYB3, TgMYB4) are indicated. bHLH motif ([DE]L × 2 [RK] × 3 L × 6 L × 3R) is indicated in TgMYB3. (PDF 131 kb) [file 12870_2015_599_MOESM15_ESM.pdf]

Additional File 15. Predicted *MYB* domain protein sequences from *Tectona grandis*. Amino acid sequences of the four *MYB* transcription factors were obtained with EXPASY tool (http://web.expasy.org/). Grey shading indicates identical amino acid residues that agree with the motifs referenced by Bedon et al. (2007). *MYB-CC* type transfactor domain (*TgMYB1*) and *R2R-MYB* DNA-binding domains (*MYBR2R3*-DBDs) (*TgMYB2*, *TgMYB3*, *TgMYB4*) are indicated. bHLH motif ([DE]L × 2 [RK] × 3L × 6L × 3R) is indicated in *TgMYB3*.

>TgMYB1

Motif of the MYB-CC Type transfactor

MKVLYLYSGMQITEALKLQMEVQKRLHEQLEVQRQLQLRIDAQGKYLKKIIEEQQHLSGVLSEMPGSGVSVSGTDDICPDSSNKTDPATPAATSEPPFLDKPGKEHAPAKSLSVDESHSSHHEPQTPDSDCRVAP
SVVSPNERPEKKQRGNNVVTCTKSEMVLNNSILESSFSPPYHLPHSIFLTSEHFDHSSVGSENQLERVSGGNP

>TgMYB2

R2 region (DNA-binding domain)

WT.eEDE.L...V....G..W RcgKSCRLRW.NyLrp...RG

MLAGLRFSFSFFLFNFRELLLNTFSHAIYSYYLSYCLCRRTSGPRFCSPRQWTAEEDETLRMAVQCFEGRKWKKIAECLNDRTVLQCLLRWKRVLHPDLVKGPWSKEEDGVLIELVNKYGLKRWSTIASNLPGRR

R3 region (DNA-binding domain)

t..EE..Li.Lh..LGNKWs PGRTDN.IKN.WN

GMQCQARWYNHLKPNIEKGAWTEAEELALIRAHQSYGNKWAELTKFFPGRDENAIKTHWNSSVEEKLDMYLASGLLPKFQGLSLLSCPSHPAASSSSKAQQSSADNSVVKGGIEVEEAFECSQGLNIASSDAWTL
QK

>TgMYB3

R2 region (DNA-binding domain) R3 region (DNA-binding domain)

WT.eEDE.L...V....G..W RcgKSCRLRW.NyLrp...RG DL..R...L......L...R PGRTDN.IKN.WN

MKEKQRPSKRELNRGPWTAEEDRKLAKAVDIHGAKQWTTIAAKAGLARCAKSCRLRWMNYLRPNIKRGNISDQEEDLIIRLHKLLGNRWSLIAGRLPGRTDNEIKNYWNYHLSKKILDKGVLVAGISTKDMGSKS
DQQTVEEKTQSVTSSGAEDSKAKVDDDDADFFDFSNESPSTLEWVTKFLEFSNS

bHLH protein-binding motif

>TgMYB4

R2 region (DNA-binding domain) R3 region (DNA-binding domain)

WT.eEDE.L...V....G..W RcgKSCRLRW.NyLrp...RG t..EE..Li.Lh..LGNKWs PGRTDN.IKN.WN

MSVTSESNEKMMPKNCIDSPAADDANSGRNVGGNDRLKKGPWTSVEDAILVEYVTKHGEGNWNAVQRHSGLARCGKSCRLRWANHLRPDLKKGAFSPEEEYLIIELHAKMGNKWARMAAELPGRTDNEIKNYWNT
RIKRRQRAGLPVYPPDICLQASNENQQKGNISTFSCGDPHYLDFMPVNNFEIPAVEFKNLEVDKQVYPPAFLDIPGSSLLPQGFHSSYPDKSFISTTHPSRRLRGSEPLLHGVSATMSNTIPGGSQYRNVSYVQN
AQSFIYSSAYYHNLTFDHASSSSVLSGSHADLNGNPSSSEPTWAMKLELPSLQTQMGNWGSPSFPLPPLESVDTLIQTPPTEHTLSCHLSPQNSGLLDAVLHESETIKNSRDSSHWQSSHASSMAVNVMDASSQV
IHETGWESHGELTSPLGHSSLFSEGTPTSGDSFDEPESVEAIPGFRVKEEATFRGSMQSDNKVETTNQMFSRPDLLLALLF
