# Supplementary material for: Large-scale transcriptional profiling of lignified tissues in Tectona grandis
Source: BMC Plant Biol. 2015 Sep 15;15:221. doi: 10.1186/s12870-015-0599-x (PMC4570228; doi:10.1186/s12870-015-0599-x)
Supplement: Additional file 16: — Primers for quantitative real-time PCR. (PDF 90 kb) [file 12870_2015_599_MOESM16_ESM.pdf]

Additional File 16. Primers for quantitative real-time PCR.

Gene	Sequence	Primer size	Amplicon size
TgMYB1	5´ GCTACAGTTGCGGATAGATG 3´	20 bp	145 bp
	5´ ATTACTGGAGTCAGGGCAAATG 3´	22 bp	
TgMYB2	5´ TCCAAAATTCCAAGGTCTGTCT 3´	22 bp	128 bp
	5´ AAGCCTCCTCCACTTCTATTCC 3´	22 bp	
TgMYB3	5´ CGGAAACAGATGGTCACTGATA 3´	22 bp	239 bp
	5´ CAGCATCATCATCATCAACCTT 3´	22 bp	
TgMYB4	5´ GGATCAGAACCTTTGTTACATGG 3´	23 bp	175 bp
	5´ TGCCAGAAAGTACACTTGAGGA 3´	22 bp	
TgHsp1	5´ CAGGAGGAGAAGAACGACAAGT 3´	22 bp	134 bp
	5´ GTCACTGTCAGCACACCATTCT 3´	22 bp	
TgHsp2	5´ ATGACGAGTCAGAGGAAGAAAA 3´	22 bp	171 bp
	5´ CTGTCCAACCATATTCTCCAGT 3´	22 bp	
TgHsp3	5´ TTAACGCCGGAAATTGTAGC 3´	20 bp	100 bp
	5´ ACCGTCACTACCAGCACTCC 3´	20 bp	
TgBi	5´ TGTCGTTCTTATGCTGTTTGCT 3´	22 bp	149 bp
	5´ CGTCGTATGTGTATCGCTTGAT 3´	22 bp	
TgCES	5´ TTTCTCCAGGGTTTTCTTGCTA 3´	22 bp	149 bp
	5´ GAGTCCTTTTCAATCCTCCAAA 3´	22 bp	
