# Supplementary material for: Large-scale transcriptional profiling of lignified tissues in Tectona grandis
Source: BMC Plant Biol. 2015 Sep 15;15:221. doi: 10.1186/s12870-015-0599-x (PMC4570228; doi:10.1186/s12870-015-0599-x)
Supplement: Additional file 18: — Some transcripts obtained from RNA-seq in Tectona grandis and used for subsequent analysis. Four MYB transcription factors: TgMYB1 (NCBI Accession number KR092428), TgMYB2 (NCBI Accession number KR092429), TgMYB3 (NCBI Accession number KR092430), TgMYB4 (NCBI Accession number KR092431), three heat-shock proteins: TgHsp1 (NCBI Accession number KR092432), TgHsp2 (NCBI Accession number KR092433), TgHsp3 (NCBI Accession number KR092434), carboxylesterase: TgCES (NCBI Accession number KR092436), bax inhibitor: TgBi (NCBI Accession number KR092435). In yellow, the methionine. In green, the stop codon. In grey, the coding sequence. In blue, the real-time PCR primers. (PDF 125 kb) [file 12870_2015_599_MOESM18_ESM.pdf]

Additional File 18. Some transcripts obtained from RNA-seq in *Tectona grandis* and used for subsequent analysis. Four *MYB* transcription factors: *TgMYB1* (NCBI Accession number KR092428), *TgMYB2* (NCBI Accession number KR092429), *TgMYB3* (NCBI Accession number KR092430), *TgMYB4* (NCBI Accession number KR092431), three *heat-shock proteins: TgHsp1* (NCBI Accession number KR092432), *TgHsp2* (NCBI Accession number KR092433), *TgHsp3* (NCBI Accession number KR092434), *carboxylesterase*: *TgCES* (NCBI Accession number KR092436), *bax inhibitor*: *TgBi* (NCBI Accession number KR092435). In yellow, the methionine. In green, the stop codon. In grey, the coding sequence. In blue, the real-time PCR primers.

```
>TgMYB1
GATCAGGGAAGGCCAAAAAAGAAATGATGGGTTAGCTTTGGTGTTTTATTCCATCTATATGAAGAAATCTTTTGGAGCTTG
TAATAGATATAATCCTCTCTGCTGAGTAATTGAGAAAGGAAAAGATATGGAACCTGTTTAAACACAATATGCTGCAAGAAA
TCGTAATGTGCATTAATGTGTGAATTACACTAAGGCAACAAGGTTTGTTGGCAATAACTGTAATTTCTCTCACTGAAAGGG
CCCTGGTCGGTGTTGTTCTAAATATTCTGAAAGAAAGAATCTTGGCTTGGTTTCTGGTGTCTGAAAAGACGAAAACACCTA
ATATTTGCATCCAATATTGAGAAAATGCCAAATTGTTTAATATTTTCTGTAATTATATTTTTAGGTGATGGATTTCAATGG
ATAAAGTCTAAAGTCATTTGCACAAGAACATTTTTCTGATACTTATTACTGTATCTCAGGGGCTACTCCTAAAGGTGTTCT
TAGAGTTATGGGAGTTCAAGGGCTAACAATTTACCATGTAAAAAGCCACTTACAGAAATACAGGCTCGCCAAGTATCTTCC
GGAATCTTCTTCTGATGGGAAAAAGGCTGAAAAGAAAGAATCTGGAGACATGCTTTCCAGTTTCGATGGTTCATCGTAAGT
GGAAACTCCTTACTGATTTTTGTCCTCCATTCCTTCGAATTACCAACAGATTTGCATGGCGGTTCTATAATTTTTCCAACG
CAGTCACTTGGCAACTATTCATGAGATCATTTTTTCCTGTTCTTATCTCTTCATGTGTAATTTGTTCTATGCTGGCGTTTC
TTTTGTTATTAGTCTATCATCCTAAGGCTCCACTGCCATGTATAATAATTATTATGACAGGACATGTATTATAATTATTAT
GACAGGACATTTCTAACATGAAAGTTCTTTATCTTTATAGCGGAATGCAAATAACTGAAGCGCTCAAGCTGCAGATGGAGG
TTCAAAAGCGATTGCATGAGCAATTGGAGGTGCAAAGACAGCTACAGTTGCGGATAGATGCCCAAGGGAAGTATTTAAAAA
AGATAATTGAAGAACAACAACATTTAAGTGGAGTTCTTTCAGAAATGCCTGGCTCAGGGGTTTCTGTATCTGGAACAGATG
ACATTTGCCCTGACTCCAGTAATAAAACTGACCCAGCAACCCCTGCTGCAACATCAGAGCCACCTTTTCTAGACAAGCCTG
GCAAAGAACATGCTCCAGCCAAGAGTCTTTCTGTTGATGAATCCCACTCCTCACACCATGAGCCACAAACCCCTGATTCTG
ATTGTCGTGTGGCTCCATCAGTTGTGAGCCCAAATGAGAGACCAGAGAAAAAGCAGCGTGGAAACAATGTTGTCACATGCA
CTAAATCAGAAATGGTCCTGAACAACTCAATACTGGAGTCAAGCTTTAGTCCTCCTTACCATCTGCCGCATTCAATTTTCT
TGACAAGCGAGCACTTTGATCATTCATCTGTTGGCAGCGAAAATCAGTTAGAAAGAGTCTCTGGTGGCAATCCGTAACTCA
TTCTGGTCTATGTTGCTTGCTTCTTGGGAGCTGCTCAATGGTCACCTTGCACGTCTATTTTCTGAAACACATAAATACATT
AGGTCAAGAAACCTACTGGTATTTAGGTTTTATGAGAAGAATATTAGTTTTAGAAGCTTTTGTTGAGGATTTTCCTGAAGC
TTTAATTCTGAGCAGGTGAATACTCAGATATTGCCACTATTGCCGCTCCATGACCGCTTGTACACATGTTTCACCACTTGG
GCCTTCGAATGGGATGGTTCGTTCATCTGGGGGCTTGAATCCGTTCTCCTGTACCTGGCCTTTTACATAAATGACTCTTCT
GATCTGCACTTTTCGTTGTTTGTCTTTCCTTTTTCCTAATGATCCTCCACCAGACACAGAATGTCATTTGCATTTTCTTAC
TGAGTTGAAGTGGTGAGGCGTCGGATTCACTCTAATCCAACGTACAGGAAAATAGTTCAATCAATGGGAATTGCCGTCCAG
CAACTGTTTTTGCAGACTCCAGTTTGTAGAGTGAAGGCAAAAGCATTCTGATGGGTCAAAAATGTAATGGGGAAACGGCAC
TGACAAAAATTCTATATATAATTTTGTGTTTAGGTATTCTGGTATTTTGT
```

```
>TgMYB2
TTCGCACCTTTTGCCCTCGGCGCGCACCAACTCTCTATTTCTCACTCGCTTTTCAAATTTCACCGAGCTTTTTATTTCATC
CTGGATTCAGACAGTGCCTACCTTGTTGTAAACGATCCTGGAATTGCCGACATGTCGGAGGTGGCGTATTCATATACTTGT
CAACTAAATACGGAAATTGATAGGGCAGACAACAATAAGGCATCAGATATTGACAGCGATGGTCTACAAAGAGCTCGACAT
TTATATCGGTGACTTTTTCTCTATGAAGCACATTGAACGCTGATTCTTCGTATTGTGTATCTTTTTGTTCTTTTATCTGCA
TCCTGCTGTAATGTATGACATGTGTATGATGAAATTTAGGAGATGAAGCAGTTTTCCTAAATTTCAGTGGTACAATACATA
AGTTTTGAGGGGCTTGTGCATTGTACTTTAATACTACTAGTGTGTGTTAACTGGTTGACATTATTGGAGTTGTATAATTTC
TGAACATATGCTGGCTGGCCTTAGATTTTCATTTTCATTTTTTCTTTTTAATTTTAGAGAACTTTTACTCAACACATTTAG
TCATGCTATATACTCTTACTACCTTTCGTATTGTTTATGCAGGAGGACGAGTGGCCCAAGATTTTGTTCCCCTAGACAATG
GACTGCAGAAGAGGATGAGACATTGAGAATGGCTGTTCAATGCTTCGAAGGGAGAAAATGGAAAAAGATAGCGGAGTGTCT
CAATGATCGAACAGTTCTTCAGTGCCTGCTTAGGTGGAAGAGAGTTCTTCATCCGGATCTTGTGAAAGGGCCATGGTCGAA
AGAGGAGGATGGAGTATTAATTGAATTGGTCAACAAATATGGTCTAAAAAGGTGGTCTACCATAGCATCAAATCTTCCTGG
ACGCAGGGGAATGCAATGCCAAGCAAGGTGGTACAATCATCTTAAGCCCAACATAGAAAAAGGAGCTTGGACAGAGGCTGA
GGAATTGGCTTTGATCCGCGCCCATCAGAGTTATGGAAACAAATGGGCAGAGTTAACTAAGTTCTTCCCTGGGAGAGATGA
GAACGCCATTAAAACCCACTGGAATAGCTCCGTTGAGGAGAAATTGGACATGTATTTGGCATCAGGATTACTTCCAAAATT
CCAAGGTCTGTCTCTTCTGAGCTGCCCTAGTCACCCTGCAGCTTCCTCTTCTTCCAAGGCACAGCAAAGTAGTGCGGATAA
TAGTGTTGTTAAAGGTGGAATAGAAGTGGAGGAGGCTTTTGAGTGCAGTCAAGGTTTGAACATTGCCAGCTCTGATGCTTG
GACACTTCAGAAATGAAGGATCACCCATGTCTTTGGGTAATGACACTGGTACTGGAGTAGCTTCTGTATAGGATCCTTTAA
CAAAGTAGTAGACGCTTCATACTTTTTAAAAATAAAAAAGAAAAGAAAATGACACTTCATACCTTATACTCTTTTCTTCTA
```

AAGTTTTTGCTGAACTCTTTATGTGATGTCACTTTGTCATATGTGTTGGTGGTTTGGATTTTTTCACTTGTAGTTTGAAAT
GTGGTACTTTGAACTAAAGTGAAATGAAGA

>TgMYB3
CTGGCTCAACAGCCGGCCCCTCAATGAAAGAGAAGCAGCGGCCATCAAAGAGGGAACTCAACCGAGGGCCATGGACGGCGG
AGGAGGATCGGAAACTAGCCAAAGCCGTCGACATCCACGGCGCTAAGCAGTGGACCACCATTGCTGCAAAAGCAGGGCTAG
CGCGTTGCGCCAAGAGTTGCAGACTAAGATGGATGAATTATCTGAGGCCGAACATCAAGAGAGGCAATATATCTGATCAAG
AAGAGGACTTGATCATCCGGCTCCATAAACTCCTCGGAAACAGATGGTCACTGATAGCAGGAAGATTGCCGGGTCGAACAG
ACAATGAGATCAAGAACTACTGGAACTATCATTTGAGCAAGAAGATATTGGACAAAGGGGTATTAGTTGCAGGAATTTCGA
CGAAAGACATGGGCTCCAAAAGTGATCAGCAAACTGTAGAAGAGAAGACACAAAGTGTTACTAGCAGTGGTGCAGAGGATT
CAAAAGCAAAGGTTGATGATGATGATGCTGATTTCTTTGATTTCTCCAATGAGAGCCCTTCAACTTTGGAGTGGGTCACCA
AATTTCTTGAATTTAGTAATAGTTGAATTTGTGTTTTAGCACTTTCCACGCTTGAATTATGTCAAGCTTGGAGACCAAGTT
AAATTTGGTGGTGACTTACTATTTTAAATTATCTTATGCTATGTTTCAATACCCACGCACACACATGGGCGTGTGCGGTTG
TAATGTAAATGGAAGGTGTGATATCGAATGAACATACTTGCTTTAGTCAAAGTTCTCTCAAGAAGAGTTCAATCTTTTCAG
AAGAAATGACAAAAGGGGAGAACATAGCAGAAGTTTACCATTTCTTTCATAGCACAAGTTTCAATCAGCAAGGAATCTGTT
TATGGGATGAAAGTAACTCTATACAAAGAATCTGAAGAAATGACCAAGGGGAGAAAATAACACAAGTTTCCAATTTCTTTC
ATAGCACGAGTTTTGAACAGCAGAGAATCTGTTTGTGGAATGAAAGTAACTCTATGCAATTCTTTGAATGCAAGGCACCAT
TGAGGATCCAGCAGTTTCACCAACAGCACCTCCAAACTGTCCATCTACCACCGCTGTCATAATACAGTAGAACTTGATTAT
TGAAAATAGAATGGCATTAGTTGAAACACAGTTTTGAAAAAGAAAAACTAGACGTGTTAACATGCTACTGCTGCTAAGCCA
TCCTGGAAATTGCACATCAATATTTGGAACAGTTGAAGGCGAACTGGGATCTGAACGTGTGCCATCAAAGTGGCGAATATA
ATTAGGAATTTGGGATCTTTTCAGACACTAGTGCTGCCAGGACATTCAAAGGCTCAGTGCTTGTAAAAAATTTAATACGAA
TGCAAACTCAGTCATGGATAGAATAAAATGTATATTCCCTATGTTATGTAACACAAAGCAGGCAAGGAACATAATATTCAG
CAGCCATGTGAGACAAGATTCACCACAGGTTCATCAAAACTTGTAACAAACCACCAAACAGGCTGAAAACAGCTCAAATAT
GTTCAAAACATGCATTGTTAGGTTTATCTACAATTTACTCAGTCATCAACATCAAAATCAAACTACTCAAAATAGCTAAAG
AAAAAGGTCAAATTGAGTATATTCAAATATATATTATGCGAAGAAAAGAATAGAAGAAACAAAAGTAGTATATAGAAGAAA
CAAAACTAGTATCAATACAACCTATATTCACTTTTATGAATGTAAAGAACCAACTCTTGCTGGTAATTAACCCCATGCCAT
GGAACACGCATAGTTGACCACCAATTTGCCATCAATGCCGAAGTTCTGCAATGCAATGAACAAATAGTTAACAATACACAC
AAAAATCAGCGAAATAACTGTGTCTCTCGGAAGAAGGTTATCATGCCATATTTCAGGAGAACCAAGAGAATGTAAAGCAAA
TGAATACAAAATCTTATTCTATTACCATGAGAATTCAAATAACATTACAGTGACCAACAAAAGAAAATCAATTATTTGATA
GATGCCAAAAAAGAGAACTGAAAAATAAGCATATCTGGTTATCACTACCAACACACTCCATAATCCTCATATGCATTGGTA
TTTTATGTGCTTGGAGAGCTGAGTCTGGAGCATAAACAATACACTGGCAAATTTGTATACAGTGACACTCCACTAAAGCAA
AACCTGAAATAATTGCATCAGCAAATGGAATGTTCTTTTTGACAACACGGCAAGAAGATGGGTCCTTTTAACATAATCCTA
GAAACCAGAAAGAAGTACTGATTATAAATTGTATGGCTAATACACAAATTTATTTGCCTAAGAACAAAAATGACAGTTCAC
ACCTCATCCTCCCATATTCAAAAAGATATCATGAAAACAACAACTCACATTATACAGGTCTAAGACCAACTCATCAGGATT
CGGTGAGAAGGCACTGTTTACATATACGAACAATGTCTCCCTGTGAAGCTGACGACGAAGAAAATCTATTACTTTCACAAA
CTTGTCAGTCCCAGCAATCTTGAATTTGGCTTGTTTGAGAATCGAAGCGTCGCCGGTGGCTCGCAGATGAACTACCACTTT
TCGAGTTTCCGAAGCCATCCGAAAGAAACTGCGGTATACCTTTTTTTT

>TgMYB4
TTTGTTTTTTGGGTTAAAGAAAACAAATATTTAAGCTTTATTGGGCTTTCTGATTCACATTGTTCAGATTTTGATATCGTG
TGTGCCTTTAAATAAAATTGCTGCTGGTTTTGTTTAGTGGTGTTTCCCAGAATAATTTTGGGTTTGGAAATTTTTCCACCA
TTTCTTAGGTTCTTCTACCTATTCTCCCAAGAAAAGGAATCTGAGTTCTTGAAAATTATCATATGCTTTTCGGGGACTGAT
CATCTGCTGCAGAAATATGTTGCATTTTATTCTACAGTCTTATGAGATGGGATCTCTGTTATGAACTGAGTAATGCTGTTT
TGGACTTTATATTTTGGGCATGACTGATCTACAGCTTGGAAGTGGGTGGAATGTTGTTTACTCTATATCTGCCCATACTGC
GTGTGAGATTTAGAGAATTTCCATTGCAGGTCCCAAGCCATTTGAAAATGTTTGGTGTTTTTTGAGAACAAAGAAAACAAA
GATGAAGCTTTGGTTCTGCATGGTTTTATTTTTAAGAATTCTTCTGCAGGGTGCATAAATGGAAAAATGTGTTTTCTTGTA
TAGTTTTTTGTCAAATCTTGATGTATATTCCACATTTAGTGTTCGCTGTAGCGGTAATTCAAAGTCTTTTTGCCAAATCAT
CATGCTTGTTGTATCAGAAGCTGTCACTCGTATAATTGGTCATATAATTGCTTTGAATAGTTTGATAGTCACATTCTCCTG
TGGAATGTACCTGTTAATCATTTTTGGCATTGATCCCACCTATACTTTTTAGCTTGTCTTCGTACGTGTTTAATAAGTTTT
GACTCATAAGCATTAGTTGGTTTGGTGACTAAAAAGAGGTTGAGTGGAACTTTAGCTTGCTTCCTTCATATGACTTTGATG
ACATCAGAAATGAAGTGGTTTCAGTTTTATAGGATCTCGGGGAACACCGTTGGTCTGTTAGAGAATTCAGAATTGTTGTGT
GCTGTCTCCTTTAGCTTGTCAAGCCTTGATGCTACATTAGAATACACATGCATATTCCTTGTCATTTTAGCTCCATATTTG
ATGAAAGCTCCTTTCTTTTTTAAGTGAGATTTCGTATTTTTCATTGGACTTCTTGTCTGTTGGTACGATTATGCATTTTGT
TCTCTTTGGTATCTTATGTACTATTATTCTTATTGCCTGGTCTATTAACAGACTGGGAGATGAGTGTGACAAGTGAAAGCA
ATGAAAAGATGATGCCTAAGAATTGCATAGACTCACCAGCTGCAGACGATGCTAACAGTGGAAGAAATGTTGGAGGGAACG
ATCGACTGAAAAAGGGTCCTTGGACTTCTGTGGAAGATGCAATTTTAGTTGAATATGTTACCAAACACGGAGAGGGGAACT
GGAATGCTGTTCAGAGACACTCGGGGCTCGCCCGTTGTGGCAAAAGTTGTCGTTTGAGGTGGGCAAATCACCTGAGACCTG
ATCTAAAGAAAGGTGCATTTAGTCCAGAGGAAGAGTATCTTATCATTGAACTTCATGCCAAGATGGGAAATAAATGGGCTC
GAATGGCTGCTGAGTTACCTGGCCGCACAGATAATGAGATAAAAAACTACTGGAACACTAGAATCAAGAGAAGACAACGGG
CGGGCTTACCAGTCTATCCACCTGATATCTGTTTACAAGCATCAAATGAGAACCAACAAAAAGGCAATATAAGCACTTTCT
CTTGCGGGGATCCACATTATCTAGACTTCATGCCAGTTAACAACTTTGAGATTCCAGCTGTGGAGTTCAAAAACTTGGAAG
TGGATAAGCAGGTATACCCACCAGCATTTCTTGATATCCCTGGTAGTAGCTTGCTGCCACAAGGTTTTCACTCTTCTTACC
CAGACAAGTCTTTTATCTCAACAACTCATCCATCCAGGCGCCTTCGAGGATCAGAACCTTTGTTACATGGTGTAAGTGCCA

CAATGAGCAACACTATTCCGGGAGGAAGTCAATATCGAAATGTTAGTTATGTGCAGAATGCTCAATCTTTTATATACTCTT
CTGCATATTATCATAATTTAACTTTTGATCATGCATCATCCTCAAGTGTACTTTCTGGCAGCCATGCTGATTTAAATGGCA
ATCCTTCTTCTTCAGAGCCCACTTGGGCAATGAAGTTGGAGCTCCCTTCACTCCAAACTCAAATGGGCAATTGGGGCTCAC
CCTCCTTCCCATTGCCTCCCCTTGAATCTGTTGATACTTTGATCCAAACCCCTCCAACTGAACACACTCTATCATGTCACC
TTTCACCCCAAAACAGTGGCCTATTGGATGCAGTACTGCATGAGTCAGAAACCATAAAAAATTCAAGGGACAGCTCTCACT
GGCAAAGTTCACATGCTTCCAGTATGGCCGTGAATGTGATGGATGCTTCATCTCAAGTTATCCATGAGACGGGATGGGAAT
CACATGGGGAACTAACCTCCCCTTTGGGTCATTCTTCTTTGTTCAGTGAAGGCACCCCTACCAGTGGGGATTCATTTGATG
AACCCGAATCTGTAGAGGCAATACCAGGATTTAGAGTTAAAGAAGAAGCAACCTTCCGGGGTTCAATGCAATCCGACAACA
AGGTTGAGACGACAAACCAGATGTTCAGCAGGCCAGATTTGTTGCTTGCCTTACTGTTCTAAGAAAGACCACACAGGAACT
ATTCCATGCTCAAAGATGCCACAGGGCAGTTTTGATTTGAGCAGGGATTGCAAGCAAATGGACACATCTGCTACATTCATC
AGCTTACCATGCGAATGATTCTAGTGCTTGGAATGCTACATCTACTGTCTAATCACTTTTATCTCAGTCGATGAAATGATC
GGACTTCAAAAGGCTCTGGATACCTTTTCCATCTTCAATGGTTATTTTTTCTGTGGACTGAAGTTCTCTCAGCTCTGTCTA
GAATACCAGGCAAGAAATGGCTACATAAACCAGAAGATGTCTAGGCAGGCTGGAAAGCAGAAACTATGTACTTTTGCACAT
TTTGTATGACTTATGAGAGTGTTGCATACTACATTGTGTTGCCAATTCCGATTATAATCATTATCTGATACAGAAGTTGCC
TTGGTGATTTAATACTCATTTTGGCAAAAATTAATAAAAAA

>TgHsp1
GGGATGTGGAAAGAGCAAGTATAATCTAGAAGACAATAATGCTGACCTAACAGTGGAATTTGCAAGAAACTGCGAGAATTC
TCCATTCTCCATTCATCCTATAAATTCTCCTCCTCCTTAACTCATATTCCCCACGAAAATTTTCGATCATTTATTGAGAAA
GCTTAGCAAGAAGACAACCCCTCAATTCTTTGTGTAGAAAGCTAAAATGTCCCTGATTCCAAGCTTCTTTGGCAACCGCCG
CAGCAACATCTTCGATCCATTTTCCTTGGATATTTGGGATCCCTTTGAGGGCTTCCCTTGGCCCACCGCCGTCGCTAACCT
CCCGTCCTCCGCCAGGGAAACCACCGCAGTGGCAAATGCTCGCATCGACTGGAAAGAGACGCCGGAGGCTCATGTGTTCAA
GGTGGATGTTCCGGGACTGAAGAAAGAGGAAGTTAAGGTTGAGATTGAGGATAATAATATTCTCCAGATCAGTGGAGAGAG
AAGCAAGGAGCAGGAGGAGAAGAACGACAAGTGGCACCGCGTCGAGAGGAGCAGCGGCAAGTTCCTCCGCCGATTCAGGCT
GCCGGAGAATGCCAAGTTGGATCAGGTGAAGGCGGCGATGGAGAATGGTGTGCTGACAGTGACGGTGCCGAAGGAAGAGGT
GAAGAAGCCTGAGGTGAAGGCCATTGACATCTCTGGTTAAGCAAGTCTTGTCTCTGAGTTTCATTTAATGGAAATTGCTGT
CGGGTCATTTTCGTTGTTTTTGTTATTGTTGTCGTTTTTTTGTTTCTTTGGTTTTGGTGTGGAAATGATGTTGAATTCGTG
TAATGTGTATCAGCAAAGTGTCATCTCAAAGTGCTAAATAAAGAAACAGAGTGTTTCTTCCAGCAATGTGGATTTCTTGTG
TATTGTCTCATCGGTGTGCATTTTGTGTACAATGTATTACCATCTTTATATAATATATGTTGATGAAATAAAATAAAAAAC
GAAAGCAGTTGTTAATAAAAAAAAAAA

>TgHsp2
CAACTTCGAACGGCTCCCGATCACTCCCGAACAACTACACCGCCAAAACCCTCATCAAAGGCCGATGCTGCATATATAAAT
CCGTTCTCCCCGTCTTCTCTCTGCTTCTCAGGGTAACACGGTTTTTTGCAATCGATATCTCTCCCGGATTTCTCTGCAATC
GATATCTGATCGATCAATTGGCTTTTCAATTTTGAGCGTGGAATATACAAATCAGAAATGGCGGAAGTTCAGATGGGTGAA
ACTGAGACTTTCGCTTTCCAGGCGGAAATCAACCAGCTTCTCAGCCTTATCATCAATACTTTTTACAGTAACAAAGAAATC
TTCCTCCGTGAACTCATCAGCAACTCCTCTGATGCGTTGGATAAGATCAGATTTGAGAGTTTAACGGACAAGAGCAAGCTG
GACGCACAGCCGGAGCTTTTCATCAGGATTGTTCCGGACAAGGTTAACAAAACTCTCTCGATTATTGACAGCGGTTGTGGC
ATGACCAAAGCCGAGCTGGTGAATAATTTAGGTACAATTGCAAGGTCTGGGACGAAAGAATTCATGGAAGCACTGCAGGCT
GGGGCTGATGTCAGTATGATTGGCCAGTTTGGTGTTGGTTTTTACTCTGCTTACCTGGTGGCAGAGAAGGTAGTTGTGACA
ACTAAGCACAATGACGATGAACAATATGTCTGGGAGTCGCAGGCTGGTGGTTCTTTCACTGTGACCCGAGATGTTACTGGT
GAACCATTGGGCAGGGGTACTAAAATCACCCTCTTTCTCAAGGATGACCAGCTGGAGTACTTGGAAGAGAGGAGGATAAAA
GACTTGGTTAAGAAGCATTCAGAGTTTATAAGCTACCCAATATACCTCTGGGTTGAAAAGACTACTGAGAAAGAGGTTAGC
GATGAGGAAGATGAGGAGTCAAAAAAGGAAGAGGAGGGTGATGTCGAGGAAGTTGATGAGGACAAAGACAAGGATAAGAAG
AAGAAGAAGAAAATAAAGGAAGTGACCCATGAGTGGCAGCAAATTAATAAACAGAAGCCTATTTGGCTCCGCAAACCTGAG
GAGATTTCGAGGGAGGAGTATGCTTCATTCTATAAGAGTTTAACTAATGACTGGGAGGACCACCTTGCTGTGAAGCACTTC
TCTGTTGAAGGGCAGCTCGAATTTAAGGCTATTCTCTTTGTCCCGAGGAGGGCTCCATTTGATCTGTTTGACACAAGGAAG
AAGATGAACAACATTAAACTGTACGTCAGGAGAGTTTTTATCATGGACAACTGTGAAGAGCTCATCCCCGAGTACCTTGGT
TTTGTGAAGGGGGTAGTGGATTCTGATGATCTGCCGCTCAATATATCTCGTGAAACGCTTCAACAGAACAAGATTCTGAAA
GTCATCAGAAAGAATTTGGTGAAGAAGTGCATTGAGATGTTCTTTGAGATTGCTGAAAACAAAGATGACTATAACAAATTT
TATGATGCGTTCTCTAAGAACATCAAATTGGGAATCCATGAAGATAGCCAGAACAGGTCTAAACTTGCTGATCTGTTGAGG
TACTATTCGACAAAAAGTGGTGATGAGATGACCAGCTTGAAGGACTATGTTACAAGGATGAAGGAGGGGCAGAAAGACATT
TACTATATCACTGGTGAGAGCAAAAAAGCTGTTGAGAACTCCCCATTTCTGGAGAGGCTTAAGAGGAAAGGATATGAAGTG
CTCTTCATGGTTGATGCCATCGATGAATATGCTGTTGGTCAATTGAAAGAATATGATGGGAAGAAGCTTGTCTCTGCCACC
AAGGAAGGTTTGAAGCTTGATGACGAGTCAGAGGAAGAAAAGAGGAGAAAGGAAGAGAAGAAGAAATCATTTGAGAACTTG
TGCAAGGTAATTAAAGACATTCTTGGTGACAGAGTTGAAAAGGTCGTAGTGTCTGATAGAATTGTTGACTCGCCTTGCTGC
TTGGTTACTGGAGAATATGGTTGGACAGCAAACATGGAGAGAATCATGAAAGCTCAGGCCCTCAGAGATAGCAGCATGAGT
GCTTACATGTCCAGCAAGAAGACAATGGAGATCAATCCGGACAATGGAATCATGGAGGAGCTGAGGAAAAGGGCTGAGGCC
GACAAAAATGATAAGTCGGTTAAAGATTTGGTATTGCTGCTCTTTGAAACTGCCCTGTTGACATCCGGTTTCAGCCTTGAT
GATCCGAACATGTTTGCTTCGAGGATCCATAGAATGCTGAAGTTGGGTCTCAGCATTGATGAGGATGAGACTGGTGGTGAG
GATGCTGACATGCCTTCACTGGAAGAGGTTAATGAGGAAAGCAAGATGGAGGAAGTTGACTAAGTAAATTCTCTTTTGCGC
GAGTTTTAACATTTTTGCCGCTCGATGTGCCTGGTCTTGGATTGTTCTCTTTGAAACTTCGTATGCTGTCTCTGCTGTCAT
TCATGTCTTTGGGTGTGTACTACAAATATCGGTTGTAGCTAGTAAATAAGGAACAGACTTTGCAGTGTTGTATATGCGGAC

TTGAGTTACGTATTAATATCAATAATGCACGTAATGCTCCTAGATGGTCTTCTTCCGTGGTTGTCATTTCCTTTCCCTCCC
TGAAGGGTCACATTTCGTCTGAAATTAGTGGATTAACCCCTAAACTTCTTATCTGGAGAAAAATTAATGACATGATTTTAG
AAGATTTAATGTTGAGCAAGTATTTAAGAAATGGCTCAAGATGAAAAATGAAAGTTTTAATCTGTCAAAATATG

>TgHsp3
GCAACATTCTATCATCTTCCACAAGAAACCCAAAAAAAGATAGAAAATCAACTATGTAGAAAAACATTCTGCGCTCCTTCT
CGAAAACCTAGTCCAGAATTTTCAAGAAATTGTTCACTGTCCAAATCTAGCACACCCCAAAACCTGTTATAAATATCAACC
AAACCTGGGATCCATCCCACACGAAGAATCAGCTGCCAATATTCCACATTTCTTACGTTTCAAAGTCCTCAGAAATTGATC
AAGAATAAAATCTATCATGTCAGTCTTCCCACTGCGATCACTGCTCTCCGATCCTTTCTTTTCAGACGTGTTCGGGCTCGG
CCCTTCGAAAGCCGGGTTGTCCATGGACTGGAAGGAGACCTCAGAAGCCCATATCTTCAAATTCGACCTTCCAGGCCTTAC
AAAAGAAGACGTGAAACTGCAAGTTCACGACGACAGAGTGCTGCACGTAAGCGCTGAGAGTAAAGAAGATGAAGATTATGG
GGAAAAGAACAATTATAAATGGCATTGTAAAGAGCGCGTTAACGCCGGAAATTGTAGCCGGGAATTTAGGTTGCCGGAGAA
TGCATTGGTGGATCAGATAAAAGCTTCGATGAGCGACGGAGTGCTGGTAGTGACGGTGCCTAAAGATCATAGTAAGAAGAA
GAAGAAGCATGGTAAGAAACATGCTGTTGAGATTGGTGGTGAAGGAGAGGATTCGGGCTCTGCTAAAGGGATTGGGCGATT
TGTTTGCTGCAAAGCCTGATTGATTGCTATTACAAATGTTTCTGTTAGAAAAGATGCGATTTTGGTTCTTGTTTTTTCCCC
TCAAAAGTGTGTGTCTAAGTTGTTTAAATCGGTCGAGTGTAATGGTTATGGGGTATAACGGAAATGGCTGTAGCTTAAATA
GGTGGCGTGTGATTCTGTGTGGGATAAGTTTCAGTTGGGATTTGTTTTGTGAAATTCGTCTTCTGATTGTAGATATGAAAC
TTAAATGATTGTATAGTGGCAATGTCAAAAGTTTTCCAAACGCGATCCCCTATTTTGAAGTAGCGTTTCATGTTCTGTTCC
GGTGGTAGGATTAAATTTCATTTAATTTGCATAAATAAGAGTTTCTAAAAATACACAATAATTGCAATAA

>TgBi
TGCACGCGACCCATCAAGAATTTTCCAACTCGACCAATCCTCACAATATATAAACTGCCGTCGCCGTGAAGCAATTCGAAA
CACCCTAAATCGGCAACCGTTTCTAGCAGAGAACGAAGTCAGCAACAAAAGACCAGGTTTTTCGTGAGTCAATAATATTGA
GACTTGTTGGAGTTGTTTGGAAAAATGTGGGCTCAGCCTCACCGGAAAGATGACGTGGAAGCCGGCGGCAGGCCGCTGTAT
CCGATGATGGTGGAAAGCCCGGAATTGCGGTGGTCGTTTATTCGGAAAATATATTCGATAATCAGTATTCAGTTGCTTCTC
ACAATTGCTGTTGCTGCTCTTGTTGTTTCCGTTCACCCAATATCGCGCTTCTTTGCCACAACTGGGGCTGGTTTGGCGCTT
TACATTGTTCTCATCATCACCCCTTTTATTGTTTTGTGCCCGTTGTATTACTATTACCAGAAGCACCCTGTGAATTATTTC
CTTTTAGGGATATTCACTGTGTCGCTGGCATTTGTAGTGGGCTTGACCTGTGCGTTCACCAGTGGAAAAGTTATTCTGGAG
TCTGTTATCTTAACTGCTGTCGTGGTCGTGAGCCTCACTTTGTATACATTTTGGGCGGCAAAGAGGGGCCATGATTTCAAT
TTCCTGGGACCTTTTCTGTTCGGTGCCGTTGTCGTTCTTATGCTGTTTGCTCTGATTCAGATTTTATTCCCTCTGGGTAGG
ATAAGCGTGATGATCTATGGCTGTCTGGCATCAATAATCTTCTGTGGTTACATTATTTACGACACCGATAATCTGATCAAG
CGATACACATACGACGAGTACATTTGGGCTGCTGTTGCTCTGTATTTGGATGTCATCAACCTGTTCCTGTCTTTGTTGACA
GTATTCAGAGCTGCTGAGAGTTAAAGGCAGTAGGAAAGCTACTGGGTCACATTTGATATTGAAAAATAGTGTGTAAATAAT
GAGTTTCTGTTACATTTGGTCCTAATTCAGTAATTTGATATGAACTGGGGAAAGTAATGAGAAATGTTCAGAGCTCATTTT
CTTGTCAAATTGATTAAAAAATGTCATCTGGAGTTGATCTGTTTACGCTTCCTATGTGGAGAGTTGAAATATACATCAAAT
TTAGCGTTCACAATTGTAACTGCCAAAAAAAAGAAAAAAAGGAAATGGGTAGTTACAAAATGTTCAGTCACAAGTGCTAAA
CTAAATGTTGGTACAAATCTTGCAGCCATTACTATATTTTCTTTCATGCTGTAAGAGAAATCAGACAAATGAAAGTTGCTC
TGATGAATTCCCTTATTCTTTTAGGCGATGGAAGTAATATTGACTTACATTACTATCATTAAACCTGAAGTTATTGGGATG
TCATTTTGGCCCGACCAGGACTTTCTAAGAAAATTTCAGGAAATTCAGACACAACGTGAAATTAAAGAGATTCA

>TgCES
ATCCAAGCAATCATCCCCTTAGCCCTCTATATAAAACAGGCAAGGGCAACTGGTTTCACTCAACACTCTCACTCTTCTTCT
TCCTCCTCAATTCAACTCTGCAAATGCAGAAGCCCAGTCGCGCTCTTCTTGTGCTCTTCCTTGTTTGCCTTTTATGGCGAA
CAATCGAGAGCCGACATACTTGGCAAGAAGCATTTAAAGTCCTCGGAATTTCATGGAATAAAGACGGCACCTTAAACAGAA
GTATTCTAATACCGATGGAGGATCCATCTCCATATGCAGACTCCGATCCCCCACCCAAAGCCCTTTCACAGGACATTTATC
TCAGTGCAAACAGTAAAGCCTATGTCCGTCTCTATATCCCCGTCGACCCTCCCAAAAACCGTAAGCTCCCTCTAATAATCT
ATCTCCACGGCGGCGATTTCGTCTTGTACAGCGCTTCCACTGTCATCTTCCACAACTTCTGCAACGATATCGCCTCTGACT
TCCCTGCCGTTGTCGTCTCCGTCGAATACCGTCTTGCTCCGGAGAACCGCCTCCCCGCCGCCTACGACGACGCCCTCAACG
CCATCTTCTGGGTGCAAAACCAGGCCCTGGGCGTCGGCGGGCGGACCCCTGGCTGGAATACGCTGATTTCTCCAGGGTTT
TCTTGCTAGGCAGCAGCGCCGGAGCCAACATAGTGTACCATGCAGCGCTGCGGGCTCTGGATTTCGATCTGCAGCCATTGA
AGATCAGGGGGTTGCTTCTGAATCAAGCTTATTTTGGAGGATTGAAAAGGACTCCGTCAGAAATCAGGCTGATTGATGATC
AATATGTTCCTCTGTACGTGAACGATGTGCTGTGGAGCCTGGCATTGCCGAAGAAAGCTAACCGTGATCACGAGTTCTGCA
ATCCGATCTCCGGCGGGACTTATTTGGGTCGGGTTTTCCGGTTGCCGAAGGTGTATATAAAAGGTGATTTTGGGGACCCGT
TGGTTGACAGGTCCATACTCTTGGCGCAATTCTTGGCTTCTTGCGGGGTGCCCGTTTATTATCGGTTCAACCCGGGAGGAT
TCCATGGAATTGAACTCCAAAACACGACGGCGGCTCAGCAGCTGTACAATGACATAAAGTGGTTCATCAACGATAACTATG
GACTTGAGATGCCTCAGGGAATCACTTCTCTTCATGCTTCCATGTAATAAGTGGTACGTGTATAGTTCATGCTTCCATGTA
ATAAGTTGTACGTGTATAGTTACTTTAGATATGAATAAAGATGCACCTACCGCTGTTTAGT
